# Biomarker Concentrations in White and British Indian Vegetarians and Nonvegetarians in the UK Biobank

**DOI:** 10.1093/jn/nxab192

**Published:** 2021-06-16

**Authors:** Tammy Y N Tong, Aurora Perez-Cornago, Kathryn E Bradbury, Timothy J Key

**Affiliations:** Cancer Epidemiology Unit, Nuffield Department of Population Health, University of Oxford, Oxford, UK; Cancer Epidemiology Unit, Nuffield Department of Population Health, University of Oxford, Oxford, UK; National Institute for Health Innovation, School of Population Health, Faculty of Medical and Health Sciences, University of Auckland, Auckland, New Zealand; Cancer Epidemiology Unit, Nuffield Department of Population Health, University of Oxford, Oxford, UK

**Keywords:** biomarkers, vegetarians, vegans, lipids, glucose, hormones, vitamin D, renal, liver, UK Biobank

## Abstract

**Background:**

Prospective studies have shown differences in some disease risks between vegetarians and nonvegetarians, but the potential biological pathways are not well understood.

**Objectives:**

We aimed to assess differences in concentrations of biomarkers related to disease pathways in people with varying degrees of animal foods exclusion.

**Methods:**

The UK Biobank recruited 500,000 participants aged 40–69 y (54.4% women) throughout the United Kingdom in 2006–2010. Blood and urine were collected at recruitment and assayed for more than 30 biomarkers related to cardiovascular diseases, bone and joint health, cancer, diabetes, renal disease, and liver health. In cross-sectional analyses, we estimated adjusted geometric means of these biomarkers by 6 diet groups (regular meat eaters, low meat eaters, poultry eaters, fish eaters, vegetarians, vegans) in 466,058 white British participants and 2 diet groups (meat eaters, vegetarians) in 5535 British Indian participants.

**Results:**

We observed differences in the concentrations of most biomarkers, with many biomarkers showing a gradient effect from meat eaters to vegetarians/vegans. Of the largest differences, compared with white British regular meat eaters, white British vegans had lower C-reactive protein [adjusted geometric mean (95% CI): 1.13 (1.03, 1.25) compared with 1.43 (1.42, 1.43) mg/L], lower low-density lipoprotein cholesterol [3.13 (3.07, 3.20) compared with 3.65 (3.65, 3.65) mmol/L], lower vitamin D [34.4 (33.1, 35.9) compared with 44.5 (44.4, 44.5) nmol/L], lower serum urea [4.21 (4.11, 4.30) compared with 5.36 (5.36, 5.37) mmol/L], lower urinary creatinine [5440 (5120, 5770) compared with 7280 (7260, 7300) μmol/L], and lower γ-glutamyltransferase [23.5 (22.2, 24.8) compared with 29.6 (29.6, 29.7) U/L]. Patterns were mostly similar in British Indians, and results were consistent between women and men.

**Conclusions:**

The observed differences in biomarker concentrations, including lower C-reactive protein, lower LDL cholesterol, lower vitamin D, lower creatinine, and lower γ-glutamyltransferase, in vegetarians and vegans may relate to differences in future disease risk.

## Introduction

Previous epidemiologic studies have reported differences in disease risks between vegetarians and nonvegetarians. Compared with meat eaters, vegetarians have been shown to have a lower risk of ischemic heart disease (1), diabetes (2), and possibly some cancers (3) but may have higher risks of fractures (4) and stroke (1). However, there is currently insufficient evidence for many other health outcomes. The development of many long-term diseases is often preceded by a change in the concentrations of relevant biological markers in the human body, which underlie the pathologic mechanisms. The comparison of established disease biomarker concentrations would therefore be helpful in predicting disease risk between vegetarians and nonvegetarians for outcomes that have not been well investigated. Moreover, for diseases where a difference in risk was previously observed, investigation of biomarker concentrations may also help to improve or confirm our understanding of underlying pathways. However, detailed characterization of disease biomarker concentrations between vegetarians and nonvegetarians is lacking, possibly due to absence of relevant data.

The aim of this study is to provide a detailed description of biomarker concentrations relevant for 6 groups of disease outcomes (cardiovascular, bone and joint, cancer, diabetes, renal, and liver) across white British and British Indian participants with varying degrees of animal-sourced food exclusion, using data from a large population-based cohort in the United Kingdom.

## Methods

### Study design and participants

The UK Biobank is a prospective cohort of 500,000 people aged 40–69 y, recruited from across the United Kingdom between 2006 and 2010 (5). The scientific rationale and design of the UK Biobank study have been described in detail elsewhere (6). In brief, people who lived within traveling distance (∼25 km) of one of the 22 assessment centers across England, Wales, and Scotland were identified from National Health Service registers and invited to participate in the study. Overall, ∼5.5% of the invitees attended a baseline visit, during which they completed a touchscreen questionnaire that asked about sociodemographic characteristics, lifestyle exposures (including diet, alcohol consumption, smoking status, and physical activity levels), and general health and medical history (7).

At recruitment, all participants also completed a computer-assisted personal interview and had physical measurements and blood samples taken. Permission for access to patient records for recruitment was approved by the Patient Information Advisory Group (now the National Information Governance Board for Health and Social Care) in England and Wales and the Community Health Index Advisory Group in Scotland, and all participants gave informed consent to participate in UK Biobank using a signature capture device at the baseline visit (8).

### Inclusion and exclusion criteria

On the touchscreen questionnaire, participants were asked to self-identify their ethnicity from options of “white,” “mixed,” “Asian or Asian British,” “Black or Black British,” “Chinese,” “Other ethnic group,” “Do not know,” or “Prefer not to answer,” with further subcategories under each option. Participants were included for our analyses if they self-identified as “white” or as “Asian or Asian British” and subsequently as “Indian,” hereafter referred to as “white British” and “British Indian.” The white British population was included as it made up the majority of the UK Biobank population (∼94%), and the British Indian population was included due to the large proportion of vegetarians in this population group (24.6% compared with 1.7% in the overall cohort). The number of vegetarians in the other ethnic groups was small and did not allow comparisons by diet groups, and therefore other ethnic groups were excluded from these analyses (*n* = 23,858). Participants were also excluded if they did not provide the relevant information to be classified into one of the prespecified diet groups (*n* = 3591), had no data on biomarkers (*n* = 3416), or had missing information on fasting time (defined as time since last meal or drink, except plain water) (*n* = 30). A participant flowchart of the inclusion and exclusion criteria of this study is shown in **Supplemental Figure 1**.

### Diet group classification

Participants were classified into diet groups by degree of animal-sourced food exclusion based on self-reported dietary data from a touchscreen questionnaire as previously described (9, 10). Briefly, on the touchscreen questionnaire, participants were asked their frequency of consumption of processed meat, beef, lamb or mutton, pork, poultry (such as chicken or turkey), oily fish, and other types of fish in 6 categories of frequency ranging from “never” to “once or more daily.” Participants were also asked whether they never consume eggs or foods containing eggs and dairy products. Reproducibility of this touchscreen questionnaire has been assessed previously, and good agreement was found for most food groups, particularly meat and fish (11). Based on these questions, 6 diet groups were defined for the white British population: regular meat eaters (red and processed meat consumption >3 times/wk), low meat eaters (red and processed meat consumption ≤3 times/wk), poultry eaters (participants who ate poultry but no red or processed meat, regardless of whether they ate fish, dairy products, or eggs), fish eaters (participants who ate fish but no red or processed meat or poultry), vegetarians (participants who did not eat meat, poultry, or fish), and vegans (participants who further excluded dairy products and eggs). Two diet groups were defined for the British Indian population: meat eaters (ate any combination of red or processed meat or poultry) and vegetarians (excluding vegans).

### Blood and urine sampling and biomarker assays

Blood sampling in UK Biobank was performed by either a phlebotomist or a nurse in all participants except for a small proportion (0.3%) who declined, were deemed unable to, or where the attempt was abandoned for either technical or health reasons. Nonfasting blood samples were taken from a vein in the inner elbow using a 18-gauge vacutainer needle and barrel or, if that appeared unsuitable, from a vein on the back of the hand using a 21-gauge Safety Lok (BD Vacutainer) butterfly needle connected to a vacutainer barrel (6). In total, 40–50 mL of blood was collected from each participant into various tubes. For most blood biomarkers, the assays were performed on serum samples, with the exception of the glycated hemoglobin (HbA1c) test, which was performed on packed RBC samples. A random urine sample was also collected from most participants except for a small proportion (0.7%) who declined, were deemed unable to for health reasons, or where technical issues occurred. Further details of the blood and urine sample collection and biomarker assay procedures can be found in the **Supplemental Methods**.

In total, 34 biomarkers were assayed, which were grouped as cardiovascular related [total cholesterol, LDL cholesterol, HDL cholesterol, triglycerides, apolipoprotein A1 (ApoA1), apolipoprotein B (ApoB), C-reactive protein, lipoprotein(a)], bone and joint related [vitamin D (25-hydroxyvitamin D), rheumatoid factor, alkaline phosphatase (ALP), calcium], cancer related [sex hormone binding globulin (SHBG), testosterone, estradiol, insulin-like growth factor I (IGF-I)], diabetes related (HbA1c, glucose), renal related (cystatin C, serum creatinine, total protein, urea, phosphate, urate, urinary creatinine, urinary sodium, urinary microalbumin, urinary potassium), and liver related [albumin, direct bilirubin, total bilirubin, γ-glutamyltransferase (GGT), alanine aminotransferase (ALT), aspartate aminotransferase (AST)] biomarkers. Although these biomarkers may not be associated with all disease subtypes within the outcome group and may also be associated with outcomes other than the one labeled, they were grouped as such to allow broad interpretation in relation to risks of the several major chronic diseases by diet group.

All measured biomarkers, with the exception of lipoprotein(a), rheumatoid factor, estradiol, and urinary microalbumin, were assessed here as potential biomarkers of interest. Lipoprotein(a) was excluded because concentrations are almost entirely genetically determined and therefore unlikely to be influenced by diet or lifestyle (12). Rheumatoid factor, estradiol, and urinary microalbumin were excluded due to the large proportion of missing data from estimates outside the reportable range (92% missing for rheumatoid factor, 85% missing for estradiol, 70% missing for microalbumin). For testosterone, in women only, missing data due to values below the reportable range were replaced with a value that was three-quarters of the lowest reportable value (0.35 nmol/L × ¾ = 0.2625 nnmol/L). We also derived additional measures of ratios of total to HDL cholesterol and ApoB to ApoA1, whereas urinary sodium and potassium were expressed as a ratio to urinary creatinine to account for dilution (13). In addition, we further examined values of serum calcium with correction for serum albumin (details in Supplemental Methods).

### Statistical analyses

Baseline characteristics of UK Biobank participants were tabulated by 6 diet groups in the white British population and by 2 diet groups in the British Indian population, as described above. The primary outcomes were 32 biomarker measures, including ratios (total to HDL cholesterol, ApoB to ApoA1, urinary sodium to creatinine, urinary potassium to creatinine). All biomarker variables, with exception of the ratios, were log-transformed to approximate a normal distribution. Adjusted geometric (or arithmetic for the ratios) mean concentrations (95% CIs) of each biomarker of interest were calculated using predictive values from linear regressions adjusted for the relevant covariates. For cardiovascular biomarkers, the analyses were restricted to participants not taking lipid-lowering medications; for diabetes biomarkers, the analyses were restricted to participants not taking diabetes medications.

For all biomarkers of interest, 4 regression models were carried out. Model 1 was adjusted for sex, age (5-y categories), fasting status (0–1, 2, 3, 4, 5, 6–7, ≥8 h), and, in the case of vitamin D, for month of recruitment; model 2 was also adjusted for BMI (in kg/m^2^; <20,  20.0–22.4, 22.5–24.9, 25.0–27.4, 27.5–29.9, 30.0–32.4, 32.5–34.9, ≥35, unknown); and model 3 was also adjusted for alcohol consumption (<1, 1–7, 8–15, ≥16 g/d, unknown) and smoking status (never, previous, current <15 cigarettes/d, current ≥15 cigarettes/d, unknown). Owing to the large proportion of missing data (22% missing overall) for the physical activity variable, physical activity [low <10,  moderate 10–49.9, high ≥50 excess metabolic equivalent of task, h/wk, unknown] (14) was also adjusted for as a fourth model. For vitamin D only, a fifth model was included further excluding participants who self-reported vitamin D supplement use. For the figures, relative geometric or arithmetic means were used by expressing the geometric or arithmetic mean biomarker concentrations in the other diet groups as a proportion of that in the regular meat eaters in the white British participants and as a proportion of that in the meat eaters in the British Indian participants.

In addition, we also estimated the adjusted arithmetic mean (i.e., based on the non-log-transformed values) of all biomarkers, based on model 3. All analyses were repeated separately for women and men in both ethnicities (i.e., white British and British Indian). For each baseline characteristic and each biomarker of interest, Wald tests were used to assess heterogeneity between the 6 diet groups in the white population and the 2 diet groups in the Indian population. For the white British population, Bonferroni-corrected pairwise comparisons between the 6 diet groups were also reported. All statistical analyses were performed using Stata release 15.1 (StataCorp), and 2-sided *P* values < 0.05 were considered significant. All figures were generated using R (R Foundation for Statistical Computing).

## Results

### Participant characteristics

After the prespecified exclusions (Supplemental Figure 1), the current analyses included 466,058 white British participants of 6 diet groups (regular meat eaters, low meat eaters, poultry eaters, fish eaters, vegetarians, vegans) and 5535 British Indian participants of 2 diet groups (meat eaters, vegetarians). Characteristics of UK Biobank white British and British Indian participants are shown in **Table 1** and separately for women and men in **Supplemental Tables 1–3**. On average, compared with meat eaters in the same ethnic group, white British low-meat, poultry, or non–meat eaters (hereafter defined as fish eaters, vegetarians, vegans) were slightly younger, whereas British Indian vegetarians were slightly older. Generally, low-meat, poultry, or non–meat eaters also had lower BMI (not in British Indians) and were less likely to report heavy drinking (≥16 g alcohol per day), heavy smoking (≥15 cigarettes per day), and lipid-lowering or diabetes medication use.

**TABLE 1:** Baseline characteristics of UK Biobank participants by diet group and ethnicity^1^

	White British participants						British Indian participants	
	Regular meat eaters	Low meat eaters						
Characteristic	(>3 times/wk)^2^	(≤3 times/wk)^2^	Poultry eaters	Fish eaters	Vegetarians	Vegans	Meat eaters	Vegetarians
Participants, *n*	221,288	222,028	5053	10,469	6804	416	4091	1444
Age, y	56.8 ± 8.1	56.9 ± 7.9	56.7 ± 8.0	54.2 ± 8.0	52.8 ± 7.9	54.1 ± 7.9	53.8 ± 8.4	55.0 ± 8.0
Sex, *n* (%)								
Women	95,613 (43.2)	141,827 (63.9)	3933 (77.8)	7587 (72.5)	4598 (67.6)	244 (58.7)	1743 (42.6)	936 (64.8)
Men	125,675 (56.8)	80,201 (36.1)	1120 (22.2)	2882 (27.5)	2206 (32.4)	172 (41.3)	2348 (57.4)	508 (35.2)
Fasting time, h	3.8 ± 2.5	3.7 ± 2.3	3.8 ± 2.4	3.7 ± 2.4	3.7 ± 2.5	3.9 ± 2.6	4.1 ± 2.4	4.1 ± 2.2
BMI, *n* (%)								
<20 kg/m^2^	3559 (1.6)	5348 (2.4)	327 (6.5)	672 (6.4)	459 (6.7)	45 (10.8)	79 (1.9)	47 (3.3)
20–24.9 kg/m^2^	57,258 (25.9)	73,626 (33.2)	2312 (45.8)	4967 (47.4)	3122 (45.9)	202 (48.6)	1286 (31.4)	442 (30.6)
25–29.9 kg/m^2^	97,483 (44.1)	93,677 (42.2)	1688 (33.4)	3539 (33.8)	2266 (33.3)	124 (29.8)	1850 (45.2)	643 (44.5)
≥30 kg/m^2^	62,276 (28.1)	48,748 (22.0)	703 (13.9)	1257 (12.0)	933 (13.7)	45 (10.8)	850 (20.8)	300 (20.8)
Unknown	712 (0.3)	629 (0.3)	23 (0.5)	34 (0.3)	24 (0.4)	0 (0.0)	26 (0.6)	12 (0.8)
Alcohol consumption, *n* (%)								
<1 g/d	33,536 (15.2)	42,857 (19.3)	1620 (32.1)	2275 (21.7)	1891 (27.8)	180 (43.3)	1702 (41.6)	1209 (83.7)
1–7 g/d	49,426 (22.3)	63,440 (28.6)	1459 (28.9)	2937 (28.1)	1886 (27.7)	100 (24.0)	964 (23.6)	146 (10.1)
8–15 g/d	47,382 (21.4)	53,521 (24.1)	1016 (20.1)	2628 (25.1)	1459 (21.4)	69 (16.6)	601 (14.7)	55 (3.8)
≥16 g/d	90,790 (41.0)	62,094 (28.0)	956 (18.9)	2626 (25.1)	1563 (23.0)	67 (16.1)	807 (19.7)	26 (1.8)
Unknown	154 (0.1)	116 (0.1)	2 (0.04)	3 (0.03)	5 (0.1)	0 (0.0)	17 (0.4)	8 (0.6)
Smoking, *n* (%)								
Never	114,120 (51.6)	123,779 (55.7)	2898 (57.4)	5828 (55.7)	3953 (58.1)	220 (52.9)	3130 (76.5)	1303 (90.2)
Previous	79,368 (35.9)	77,786 (35.0)	1756 (34.8)	3863 (36.9)	2294 (33.7)	162 (38.9)	553 (13.5)	91 (6.3)
Current <15 cigarettes/d	6755 (3.1)	6209 (2.8)	136 (2.7)	279 (2.7)	189 (2.8)	10 (2.4)	152 (3.7)	10 (0.7)
Current ≥15 cigarettes/d	20,147 (9.1)	13,396 (6.0)	244 (4.8)	467 (4.5)	345 (5.1)	23 (5.5)	213 (5.2)	23 (1.6)
Unknown	898 (0.4)	858 (0.4)	19 (0.4)	32 (0.3)	23 (0.3)	1 (0.2)	43 (1.1)	17 (1.2)
Physical activity, *n* (%)								
Low (<10 excess MET h/wk)	44,347 (20.0)	41,505 (18.7)	722 (14.3)	1634 (15.6)	1225 (18.0)	67 (16.1)	887 (21.7)	327 (22.6)
Moderate (10–49 excess MET h/wk)	88,365 (39.9)	93,296 (42.0)	2139 (42.3)	4906 (46.9)	3095 (45.5)	192 (46.2)	1493 (36.5)	491 (34.0)
High (≥50 excess MET h/wk)	39,698 (17.9)	38,797 (17.5)	1165 (23.1)	2097 (20.0)	1340 (19.7)	98 (23.6)	552 (13.5)	136 (9.4)
Unknown	48,878 (22.1)	48,430 (21.8)	1027 (20.3)	1832 (17.5)	1144 (16.8)	59 (14.2)	1159 (28.3)	490 (33.9)
Lipid medication use, *n* (%)								
No	180,613 (81.6)	187,117 (84.3)	4475 (88.6)	9672 (92.4)	6358 (93.4)	392 (94.2)	3028 (74.0)	1130 (78.3)
Yes	40,675 (18.4)	34,911 (15.7)	578 (11.4)	797 (7.6)	446 (6.6)	24 (5.8)	1063 (26.0)	314 (21.7)
Diabetes medication use, *n* (%)								
No	211,981 (95.8)	215,661 (97.1)	4986 (98.7)	10,342 (98.8)	6701 (98.5)	407 (97.8)	3559 (87.0)	1260 (87.3)
Yes	9307 (4.2)	6367 (2.9)	67 (1.3)	127 (1.2)	103 (1.5)	9 (2.2)	532 (3.0)	184 (2.7)

1 Values are means ± SDs unless otherwise indicated. MET, metabolic equivalent of task.

2 Includes participants who consume any red or processed meat, regardless of whether they consume poultry, fish, or dairy. Cutoffs of regular and low consumption determined based on consumption of red and processed meat (beef, lamb, pork, and processed meat) as reported on the touchscreen questionnaire.

### Differences in biomarker concentrations by diet groups

**FIGURE 1:**
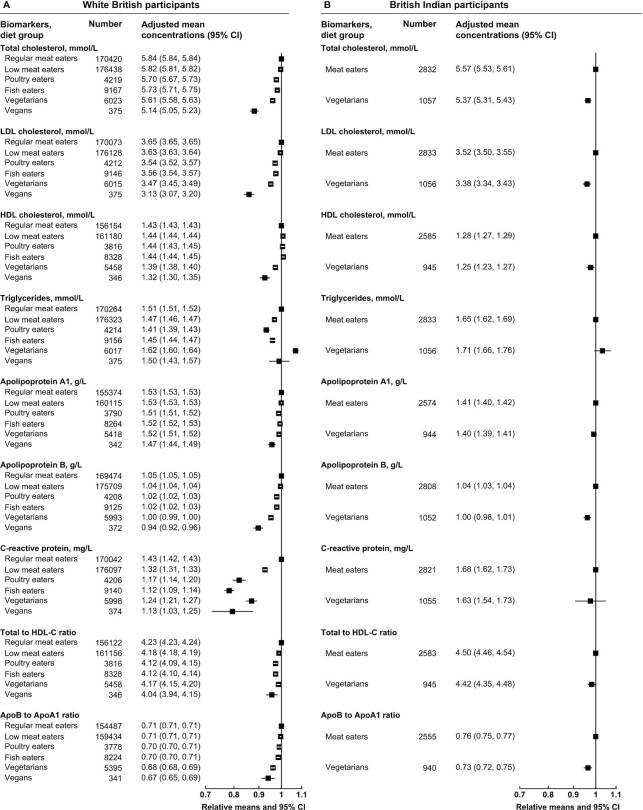
Cardiovascular-related serum biomarker concentrations by diet group and ethnicity in UK Biobank. Point estimates are relative geometric or arithmetic (ratios) means ± 95% CIs in serum biomarker concentrations compared with regular meat eaters in white British participants or compared with meat eaters in British Indian participants. Adjusted mean concentrations are adjusted geometric or arithmetic (ratios) means ± 95% CIs based on linear regression models. Total to HDL cholesterol ratio was expressed as mmol/L of total cholesterol to mmol/L of HDL cholesterol, and ApoB to ApoA1 ratio was expressed as g/L of ApoB to g/L of ApoA1. All estimates were adjusted for sex, age at recruitment (5-y categories), fasting status (0–1, 2, 3, 4, 5, 6–7, ≥8 h), BMI (<20, 20.0–22.4, 22.5–24.9, 25.0–27.4, 27.5–29.9, 30.0–32.4, 32.5–34.9, ≥35.0 kg/m^2^, unknown), alcohol consumption (<1, 1–7, 8–15, ≥16 g/d, unknown), and smoking status (never, previous, current <15 cigarettes/d, current ≥15 cigarettes/d, unknown). ApoA1, apolipoprotein A1; ApoB, apolipoprotein B.

**FIGURE 2:**
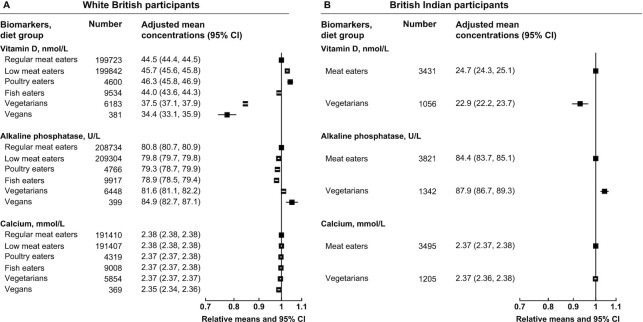
Bone and joint–related serum biomarker concentrations by diet group and ethnicity in UK Biobank. Point estimates are relative geometric means ± 95% CIs in serum biomarker concentrations compared with regular meat eaters in white British participants or compared with meat eaters in British Indian participants. Adjusted mean concentrations are adjusted geometric means ± 95% CIs based on linear regression models. All estimates were adjusted for sex, age at recruitment (5-y categories), fasting status (0–1, 2, 3, 4, 5, 6–7, ≥8 h), BMI (<20, 20.0–22.4, 22.5–24.9, 25.0–27.4, 27.5–29.9, 30.0–32.4, 32.5–34.9, ≥35.0 kg/m^2^, unknown), alcohol consumption (<1, 1–7, 8–15, ≥16 g/d, unknown), and smoking status (never, previous, current <15 cigarettes/d, current ≥15 cigarettes/d, unknown). The model for vitamin D was also adjusted for month of recruitment.

**FIGURE 3:**
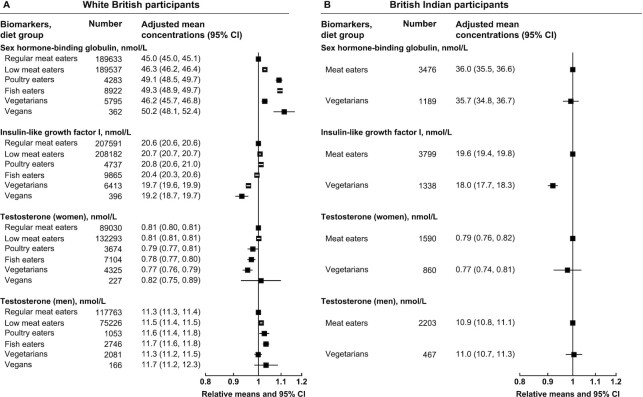
Cancer-related serum biomarker concentrations by diet group and ethnicity in UK Biobank. Point estimates are relative geometric means ± 95% CIs in serum biomarker concentrations compared with regular meat eaters in white British participants or compared with meat eaters in British Indian participants. Adjusted mean concentrations are adjusted geometric means ± 95% CIs based on linear regression models. All estimates were adjusted for sex (with exception of testosterone), age at recruitment (5-y categories), fasting status (0–1, 2, 3, 4, 5, 6–7, ≥8 h), BMI (<20, 20.0–22.4, 22.5–24.9, 25.0–27.4, 27.5–29.9, 30.0–32.4, 32.5–34.9, ≥35.0 kg/m^2^, unknown), alcohol consumption (<1, 1–7, 8–15, ≥16 g/d, unknown), and smoking status (never, previous, current <15 cigarettes/d, current ≥15 cigarettes/d, unknown).

**FIGURE 4:**
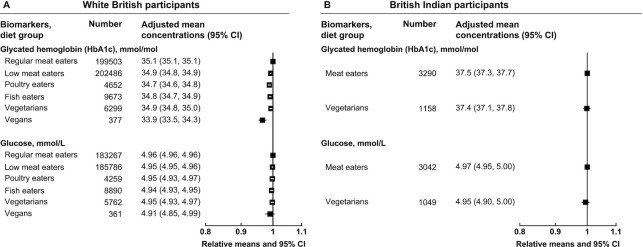
Diabetes-related serum and packed RBC biomarker concentrations by diet group and ethnicity in UK Biobank. Point estimates are relative geometric means ± 95% CIs in serum or packed RBC biomarker concentrations compared with regular meat eaters in white British participants or compared with meat eaters in British Indian participants. Adjusted mean concentrations are adjusted geometric means ± 95% CIs based on linear regression models. All estimates were adjusted for sex, age at recruitment (5-y categories), fasting status (0–1, 2, 3, 4, 5, 6–7, ≥8 h), BMI (<20, 20.0–22.4, 22.5–24.9, 25.0–27.4, 27.5–29.9, 30.0–32.4, 32.5–34.9, ≥35.0 kg/m^2^, unknown), alcohol consumption (<1, 1–7, 8–15, ≥16 g/d, unknown), and smoking status (never, previous, current <15 cigarettes/d, current ≥15 cigarettes/d, unknown). HbA1c, glycated hemoglobin.

**FIGURE 5:**
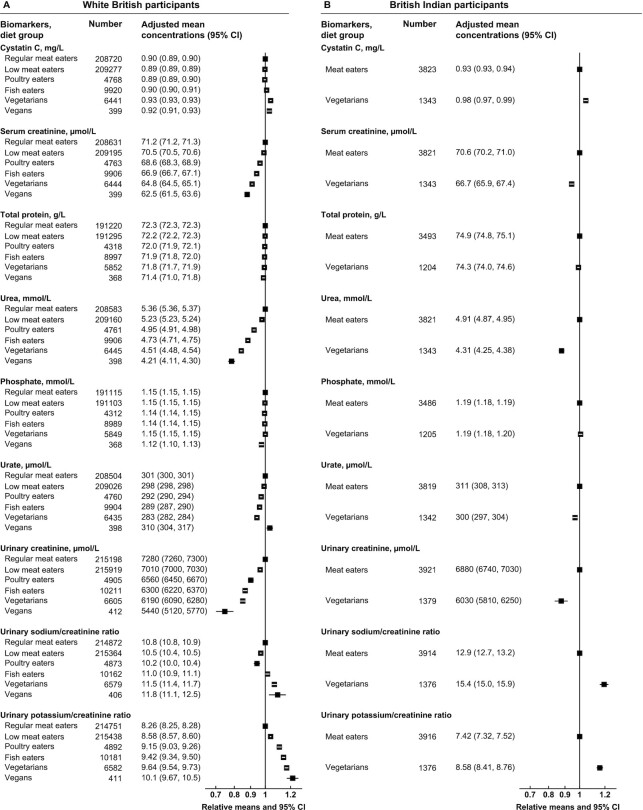
Renal-related serum and urinary biomarker concentrations by diet group and ethnicity in UK Biobank. Point estimates are relative geometric or arithmetic (ratios) means ± 95% CIs in serum or urinary biomarker concentrations compared with regular meat eaters in white British participants or compared with meat eaters in British Indian participants. Adjusted mean concentrations are adjusted geometric or arithmetic (ratios) means ± 95% CIs based on linear regression models. The 2 ratio measures were expressed as per mmol/L of urinary sodium or potassium to per mmol/L of urinary creatinine. All estimates were adjusted for sex, age at recruitment (5-y categories), fasting status (0–1, 2, 3, 4, 5, 6–7, ≥8 h), BMI (<20, 20.0–22.4, 22.5–24.9, 25.0–27.4, 27.5–29.9, 30.0–32.4, 32.5–34.9, ≥35.0 kg/m^2^, unknown), alcohol consumption (<1, 1–7, 8–15, ≥16 g/d, unknown), and smoking status (never, previous, current <15 cigarettes/d, current ≥15 cigarettes/d, unknown).

**FIGURE 6:**
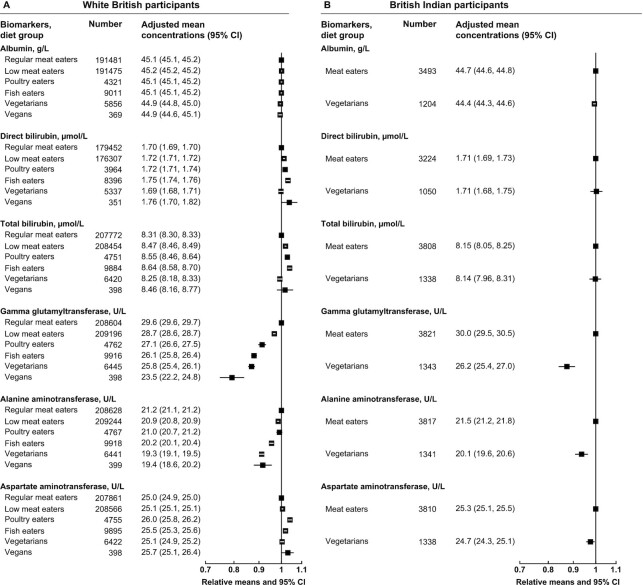
Liver-related serum biomarker concentrations by diet group and ethnicity in UK Biobank. Point estimates are relative geometric means ± 95% CIs in serum biomarker concentrations compared with regular meat eaters in white British participants or compared with meat eaters in British Indian participants. Adjusted mean concentrations are adjusted geometric means ± 95% CIs based on linear regression models. All estimates were adjusted for sex, age at recruitment (5-y categories), fasting status (0–1, 2, 3, 4, 5, 6–7, ≥8 h), BMI (<20, 20.0–22.4, 22.5–24.9, 25.0–27.4, 27.5–29.9, 30.0–32.4, 32.5–34.9, ≥35.0 kg/m^2^, unknown), alcohol consumption (<1, 1–7, 8–15, ≥16 g/d, unknown), and smoking status (never, previous, current <15 cigarettes/d, current ≥15 cigarettes/d, unknown).

Geometric mean biomarker concentrations or arithmetic mean of biomarker ratios by diet groups and ethnicities based on model 3 are shown as **Figures 1**–**6** and also in **Supplemental Tables 4–27**. For the white British population, all results described below represent significant differences after Bonferroni correction for pairwise comparisons across the 6 diet groups and by using regular meat eaters as the reference group, unless otherwise stated.

For cardiovascular-related biomarkers, compared with regular meat eaters, white British low-meat, poultry, and non–meat eaters generally had lower serum concentrations of total cholesterol, LDL cholesterol, ApoB, C-reactive protein, total to HDL cholesterol ratio, and ApoB to ApoA1 ratio (Figure 1A and Supplemental Tables 4–6). The vegetarians had the highest serum triglyceride concentrations, whereas vegans had the lowest ApoA1 concentrations, and both vegetarians and vegans had lower HDL cholesterol than regular meat eaters. In British Indians, vegetarians had significantly lower concentrations of serum total cholesterol, LDL cholesterol, HDL cholesterol, ApoB, total to HDL cholesterol ratio, and ApoB to ApoA1 ratio than meat eaters, but differences in the other cardiovascular-related biomarkers were not statistically significant (Figure 1B and Supplemental Table 7).

For bone and joint–related biomarkers, both white British vegetarians and vegans (Figure 2A and Supplemental Tables 8–10) and British Indian vegetarians (Figure 2B and Supplemental Table 11) had lower serum vitamin D (mean concentrations were slightly lower but patterns were similar in nonsupplement users) and higher ALP concentrations than meat eaters in the same ethnic group. White British vegans also had marginally lower serum calcium concentrations (results were consistent with or without correction for albumin) than meat eaters, but no difference in serum calcium was observed between British Indian meat eaters and vegetarians.

For cancer-related biomarkers, in white British participants, all other diet groups had higher serum SHBG than regular meat eaters, whereas vegetarians and vegans had lower IGF-I concentrations (Figure 3A and Supplemental Tables 12–14). In men, low meat eaters, poultry eaters, and fish eaters but not vegetarians or vegans had significantly higher serum testosterone concentrations than regular meat eaters, although the magnitudes of differences were small. In British Indians, vegetarians had lower serum IGF-I concentrations than meat eaters, but no significant differences were observed in SHBG or testosterone concentrations (Figure 3B and Supplemental Table 15).

For diabetes-related biomarkers, in the white British population, there were minimal differences in HbA1c concentrations and no significant difference in glucose concentrations (Figure 4A and Supplemental Tables 16–18); no significant differences were observed in the British Indians (Figure 4B and Supplemental Table 19).

For renal-related biomarkers, compared with the respective meat eaters in both ethnicities, the other diet groups had lower concentrations of serum and urinary creatinine, lower urea, but higher urinary potassium to creatinine ratio, whereas the vegetarians had higher cystatin C concentrations and higher urinary sodium to creatinine ratio (Figure 5A, 5B, and Supplemental Tables 20–23). In the white British population, the vegans had the highest urate concentrations, but all other diet groups (including the vegetarians) had lower concentrations than the regular meat eaters; similarly, British Indian vegetarians had lower urate concentrations than meat eaters.

For liver-related biomarkers, the low-meat, poultry, and non–meat eaters in both ethnic groups had lower GGT and ALT concentrations than meat eaters (Figure 6A, 6B, and Supplemental Tables 24–27). In white British participants, the low-meat, poultry, and fish eaters had higher concentrations of direct and total bilirubin and AST, whereas vegetarians in both ethnicities had lower concentrations of albumin, but the magnitudes of these differences were small.

For most biomarkers, differences between diet groups were similar across all levels of covariate adjustment (Supplemental Tables 4–27). Of the covariates included in the main model (model 3), in the white British population, BMI adjustment partly attenuated the magnitude in the differences of most cardiovascular-related (all except HDL cholesterol; Supplemental Tables 4–6) and cancer-related (SHBG, testosterone in men; Supplemental Tables 12–14) biomarkers. However, the patterns across diet groups remained similar, except for triglyceride concentrations, which were lower in vegetarians than regular meat eaters before BMI adjustment but higher after. For the diabetes biomarkers, BMI adjustment completely attenuated any difference in glucose concentrations and partially attenuated the differences in HbA1c concentrations. Adjustment for BMI had little influence on the same biomarkers in the British Indian population, possibly due to the more similar BMI distribution between the meat eaters and vegetarians in this population (Table 1). The additional adjustment for alcohol consumption, smoking status, and physical activity had minimal influence on the estimates; the arithmetic mean estimates were also largely consistent, and the patterns were also mostly similar when women and men were examined separately (Supplemental Tables 4–27).

## Discussion

In this large British cohort, we observed differences in the concentrations of many biomarkers by diet group. Of the largest differences, vegetarians and vegans had lower average concentrations of blood cholesterol, C-reactive protein, vitamin D, urea, creatinine (in both blood and urine), and some liver enzymes (GGT and ALT) but higher potassium to creatinine ratios compared with meat eaters. The differences in these disease-related biomarkers may reflect physiologic differences and/or differences in underlying disease pathology and therefore future risk of the relevant health outcomes.

### Cardiovascular-related biomarkers

We saw clear differences in the concentrations of many blood lipids by diet group, consistent with previous smaller observational studies and small randomized trials that also reported lower total and LDL cholesterol (or non-HDL cholesterol) (15–18) or lower total to HDL cholesterol ratio and lower ApoB to ApoA-1 ratio (15) in vegetarians than meat eaters. These differences might be explained by several characteristics typical of vegetarian and vegan diets, including lower saturated fat intake (19), higher fiber content (20), or the substitution of animal protein with plant protein (21). Lipid concentrations, especially low LDL cholesterol, have been causally linked to lower risk of ischemic heart disease and ischemic stroke but possibly higher risk of hemorrhagic stroke (22, 23), and therefore, the differences in lipid concentrations are consistent with previous observations of lower ischemic heart disease but higher hemorrhagic stroke risk in vegetarians than meat eaters in the United Kingdom (1). Although we also observed lower concentrations of C-reactive protein in low to non–meat eaters, which may suggest a role of red and processed meat in low-grade inflammation (24, 25), these differences greatly attenuated with BMI adjustment, suggesting that residual confounding due to differences in adiposity may be present.

### Bone and joint–related biomarkers

Previous studies have suggested that vegetarians or vegans might have poorer bone health than meat eaters (4, 10, 26). Similar to our study, several (27–29) but not all (30) prior studies have reported lower circulating vitamin D concentrations in vegetarians or vegans than meat eaters, suggesting that although the amount of sun exposure and skin coloration/ethnicity have a large role in determining vitamin D concentrations (29, 30), dietary habits are also important. Vitamin D has a role in skeletal health via promoting calcium absorption and maintaining muscle function (31), and recent meta-analyses have reported that daily supplementation with both vitamin D and calcium may be effective in reducing fracture risks, although vitamin D supplementation alone may not (32, 33). Similar to our findings, a small Finnish study also observed the highest ALP concentrations in vegetarians and vegans (29). This might be an indicator of increased bone turnover in these diet groups (29, 34), as although ALP could be increased due to liver abnormalities, in another study, vegetarians were not known to have a higher rate of liver diseases (35). In contrast, serum calcium concentrations are tightly controlled via homeostatic mechanisms in the body (36), and although we did observe slightly lower calcium concentrations in the white British vegans in our large study, the magnitude of the difference was very small.

### Cancer-related biomarkers

Our observation of higher SHBG concentrations in the white British low to non–meat eaters was consistent with some previous studies that reported slightly higher concentrations in vegans (37–39), although other studies have reported no significant differences (40, 41). For IGF-I, partly similar to our findings, previous much smaller studies have reported lower concentrations in vegans (but not in vegetarians) than meat eaters (39, 41). Among men, although 1 previous study reported higher testosterone concentrations in vegans than meat eaters or vegetarians (39), we only found significantly higher concentrations in low-meat, poultry, and fish eaters. Previous prospective studies have shown that higher SHBG and lower IGF-I concentrations are associated with lower risks of breast and prostate cancer (42–45), and thus the differences in these biomarker concentrations by diet group might suggest a lower risk of these cancers in vegetarians and vegans. A lower risk of prostate cancer in white vegans than nonvegetarians has been reported in the Adventist Health Study 2 (46); further research is needed to confirm any associations between vegetarian diets and other hormone-related cancers (3, 47).

### Diabetes-related biomarkers

Prior epidemiologic studies have reported a lower risk of diabetes in vegetarians than nonvegetarians, at least partly explained by differences in BMI (2, 48, 49). In our current study, differences in glucose concentrations by diet group disappeared upon adjustment for BMI, whereas differences in HbA1c concentrations were attenuated but remained statistically significant. In contrast to our findings based on nonfasting blood samples but adjusted for fasting time, some previous studies have reported lower fasting glucose in vegetarians/vegans compared with nonvegetarians, even after adjustment for BMI (17, 50). The role of BMI or adiposity as well as fasting status in explaining any differences in diabetes-related biomarkers by diet groups should therefore be further investigated.

### Renal-related biomarkers

There has been limited evidence on comparisons of renal biomarkers between vegetarians and nonvegetarians, although several small studies have reported lower serum (51) or urinary creatinine (52) or lower blood urea nitrogen (a correlate of serum urea) (51) in vegetarians or vegans, for which large differences were found in our study. Similar to current findings, previous analyses in the UK EPIC-Oxford cohort have found that compared with meat eaters, fish eaters and vegetarians had lower concentrations of serum uric acid (also known as urate), whereas vegans had the highest concentrations (53), possibly due to their exclusion of dairy products and subsequently reduced excretion of uric acid (54). Some epidemiologic studies have reported inverse associations between vegetarian or plant-based diets and chronic kidney disease (55, 56), but further data are needed to confirm possible differences in renal biomarker concentrations and risk of kidney disease by diet group.

### Liver-related biomarkers

Of the very few studies that reported on liver-related biomarkers associated with vegetarian diets, 1 Chinese study reported lower GGT concentrations in vegetarians than omnivores (57), which was consistent with our findings. Another study compared vegetarian Buddhist priests with the general population (58), and so their findings could not be readily compared with our current results since those 2 population groups were taken from different settings. In terms of long-term health outcomes, 1 previous study has reported that vegetarians had lower odds of nonalcoholic fatty liver disease than nonvegetarians (35). Overall, existing data on both the concentrations of liver-related biomarkers and difference in risk of liver disease between vegetarians and nonvegetarians are scarce, and thus further research is needed.

### Strengths and limitations

The current study included close to 500,000 white British and British Indian participants, as well as examined >30 biomarker measures, and is therefore the largest comprehensive study on biomarker concentrations by diet groups (up to 6 groups in the white British population), as well as the first report, to our knowledge, on differences in several biomarkers by diet group. We also presented results with varying levels of adjustment; although BMI was shown to be an important factor for explaining at least partly the differences in several biomarker concentrations by diet group, BMI was derived based on objectively measured height and weight in the current study, and therefore residual confounding by BMI should be minimal, although residual confounding from other measures of adiposity cannot be ruled out. Of the limitations, some self-selection bias might be present in the cohort, which limits generalizability of the findings to the wider population. Because the study is cross-sectional, causality cannot be determined. As with any studies involving biomarker assays, some degree of laboratory drift might also be present, although this is expected to be nondifferential by diet group. Finally, our analyses were focused on broad classifications of habitual diet groups with varying degrees of animal-sourced food exclusion; the extent to which any differences might be driven by individual foods therefore requires further examination.

## Conclusions

We observed differences in the concentrations of many biomarkers by vegetarian diet group in both white British and British Indian participants in this large UK population. Our study confirmed previous observations of lower blood lipids and, in some settings, lower serum vitamin D in the vegetarians and vegans, but it also provided the first comprehensive data, to our knowledge, on differences in many other biomarkers, including lower concentrations of C-reactive protein (in the white British population only), urea, blood and urinary creatinine, GGT, and ALT but higher potassium to creatinine ratio, among other differences, in the vegetarians and vegans compared with meat eaters. It is likely that many of these differences in biomarkers are due to differences in dietary composition and nutritional status between the diet groups, but it is also possible that some of the differences are due to residual confounding by adiposity and other aspects of lifestyle. Because these biomarkers are known to be associated with disease risk, the observed differences might be indicative of differences in physiology and/or underlying disease pathology and, in turn, future risks of diabetes and cardiovascular, bone and joint, cancer, renal, and liver diseases. Future work should aim to replicate these findings in other settings, and prospective studies should investigate whether possible differences in disease risk are present by diet group.

## Supplementary Material

nxab192_Supplemental_File

## Data Availability

Data-sharing statement: UK Biobank is an open access resource. Bona fide researchers can apply to use the UK Biobank dataset by registering and applying at http://ukbiobank.ac.uk/register-apply/.

## Notes

Supported by the UK Medical Research Council (MR/M012190/1), Wellcome Trust Our Planet Our Health (Livestock, Environment and People, LEAP 205212/Z/16/Z), and Cancer Research UK (C8221/A29017). TYNT is supported by a Nuffield Departmental of Population Health Intermediate Fellowship. AP-C is supported by a Cancer Research UK Population Research Fellowship (C60192/A28516) and by the World Cancer Research Fund (WCRF UK), as part of the WCRF International grant program (2019/1953). KEB is supported by a Sir Charles Hercus Health Research Fellowship from the Health Research Council of New Zealand. This research has been conducted using UK Biobank Resource under application 24494.

Author disclosures: The authors report no conflicts of interest.

Supplemental Figure 1, Supplemental Methods, Supplemental Tables 1–27, and Supplemental References are available from the “Supplementary data” link in the online posting of the article and from the same link in the online table of contents at https://academic.oup.com/jn/.

Abbreviations used: ALP, alkaline phosphatase; ALT, alanine aminotransferase, ApoA1, apolipoprotein A1; ApoB, apolipoprotein B; AST, aspartate aminotransferase; GGT, γ-glutamyltransferase; HbA1C, glycated hemoglobin; IGF-I, insulin-like growth factor I; SHBG, sex hormone binding globulin.
