# Supplementary material for: Biomarker Concentrations in White and British Indian Vegetarians and Nonvegetarians in the UK Biobank
Source: J Nutr. 2021 Jun 16;151(10):3168–79. doi: 10.1093/jn/nxab192 (PMC8485916; doi:10.1093/jn/nxab192)
Supplement: nxab192_Supplemental_File [file nxab192_supplemental_file.docx]

Table of Contents

[Supplemental Figure 1: Participant flow chart of the study. 3](#_Toc71816583)

[Supplemental Methods 4](#_Toc71816584)

[Supplemental Table 1: Baseline characteristics^1^ of white British women by diet group in the UK Biobank. 5](#_Toc71816585)

[Supplemental Table 2: Baseline characteristics^1^ of white British men by diet group in the UK Biobank. 7](#_Toc71816586)

[Supplemental Table 3: Baseline characteristics^1^ of British Indian women and men by diet group in the UK Biobank. 9](#_Toc71816587)

[Supplemental Table 4: Cardiovascular-related serum biomarker concentrations (numbers, adjusted means and 95% CIs)^1^ in white British participants by diet group in the UK Biobank. 11](#_Toc71816588)

[Supplemental Table 5: Cardiovascular-related serum biomarker concentrations (numbers, adjusted means and 95% CIs)^1^ in white British women by diet group in the UK Biobank. 14](#_Toc71816589)

[Supplemental Table 6: Cardiovascular-related serum biomarker concentrations (numbers, adjusted means and 95% CIs)^1^ in white British men by diet group in the UK Biobank. 17](#_Toc71816590)

[Supplemental Table 7: Cardiovascular-related serum biomarker concentrations (numbers, adjusted means and 95% CIs)^1^ in British Indian participants by diet group in the UK Biobank. 20](#_Toc71816591)

[Supplemental Table 8: Bone and joint-related serum biomarker concentrations (numbers, adjusted means and 95% CIs)^1^ in white British participants by diet group in the UK Biobank. 23](#_Toc71816592)

[Supplemental Table 9: Bone and joint-related serum biomarker concentrations (numbers, adjusted means and 95% CIs)^1^ in white British women by diet group in the UK Biobank. 25](#_Toc71816593)

[Supplemental Table 10: Bone and joint-related serum biomarker concentrations (numbers, adjusted means and 95% CIs)^1^ in white British men by diet group in the UK Biobank. 27](#_Toc71816594)

[Supplemental Table 11: Bone and joint-related serum biomarker concentrations (numbers, adjusted means and 95% CIs)^1^ in British Indian participants by diet group in the UK Biobank. 29](#_Toc71816595)

[Supplemental Table 12: Cancer-related serum biomarker concentrations (numbers, adjusted means and 95% CIs)^1^ in white British participants by diet group in the UK Biobank. 31](#_Toc71816596)

[Supplemental Table 13: Cancer-related serum biomarker concentrations (numbers, adjusted means and 95% CIs)^1^ in white British women by diet group in the UK Biobank. 33](#_Toc71816597)

[Supplemental Table 14: Cancer-related serum biomarker concentrations (numbers, adjusted means and 95% CIs)^1^ in white British men by diet group in the UK Biobank. 35](#_Toc71816598)

[Supplemental Table 15: Cancer-related serum biomarker concentrations (numbers, adjusted means and 95% CIs)^1^ in British Indian participants by diet group in the UK Biobank. 37](#_Toc71816599)

[Supplemental Table 16: Diabetes-related serum and packed red blood cell biomarker concentrations (numbers, adjusted means and 95% CIs)^1^ in white British participants by diet group in the UK Biobank. 38](#_Toc71816600)

[Supplemental Table 17: Diabetes-related serum and packed red blood cell biomarker concentrations (numbers, adjusted means and 95% CIs)^1^ in white British women by diet group in the UK Biobank. 39](#_Toc71816601)

[Supplemental Table 18: Diabetes-related serum and packed red blood cell biomarker concentrations (numbers, adjusted means and 95% CIs)^1^ in white British men by diet group in the UK Biobank. 40](#_Toc71816602)

[Supplemental Table 19: Diabetes-related serum and packed red blood cell biomarker concentrations (numbers, adjusted means and 95% CIs)^1^ in British Indian participants by diet group in the UK Biobank. 41](#_Toc71816603)

[Supplemental Table 20: Renal-related serum and urinary biomarker concentrations (numbers, adjusted means and 95% CIs)^1^ in white British participants by diet group in the UK Biobank. 42](#_Toc71816604)

[Supplemental Table 21: Renal-related serum and urinary biomarker concentrations (numbers, adjusted means and 95% CIs)^1^ in white British women by diet group in the UK Biobank. 45](#_Toc71816605)

[Supplemental Table 22: Renal-related serum and urinary biomarker concentrations (numbers, adjusted means and 95% CIs)^1^ in white British men by diet group in the UK Biobank. 48](#_Toc71816606)

[Supplemental Table 23: Renal-related serum and urinary biomarker concentrations (numbers, adjusted means and 95% CIs)^1^ in British Indian participants by diet group in the UK Biobank. 51](#_Toc71816607)

[Supplemental Table 24: Liver-related serum biomarker concentrations (numbers, adjusted means and 95% CIs)^1^ in white British participants by diet group in the UK Biobank. 54](#_Toc71816608)

[Supplemental Table 25: Liver-related serum biomarker concentrations (numbers, adjusted means and 95% CIs)^1^ in white British women by diet group in the UK Biobank. 56](#_Toc71816609)

[Supplemental Table 26: Liver-related serum biomarker concentrations (numbers, adjusted means and 95% CIs)^1^ in white British men by diet group in the UK Biobank. 58](#_Toc71816610)

[Supplemental Table 27: Liver-related serum biomarker concentrations (numbers, adjusted means and 95% CIs)^1^ in British Indian participants by diet group in the UK Biobank. 60](#_Toc71816611)

[Supplemental References 62](#_Toc71816612)

502,488 participants recruited, provided informed consent, and did not subsequently withdraw

Excluded 23,858 participants of other (i.e. not white British or British Indian) or unknown ethnicities

472,679 white participants and 5,951 Indian participants

Excluded 3,591 participants who could not be classified into one of the prespecified diet groups

469,445 white participants and 5,594 Indian participants

Excluded 3,416 participants with no biomarker data and 30 participants with missing fasting status

466,058 white participants and 5,535 Indian participants

**Supplemental Figure 1**: Participant flow chart of the study.

**Supplemental Methods**

*Blood and urine sampling and biomarker assays*

After sample collection, the vacutainers (both blood and urine) were held at 4°C at the assessment centre until the end of the day when they were packed and dispatched to the central processing laboratory in temperature-controlled shipping boxes, where they were processed, aliquoted, and transferred to long-term ultra-low temperature archives (-80°C), usually less than 24 hours after collection (1,2).

Serum samples (used for assays of all blood biomarkers except glycated haemoglobin (HbA1c)) were collected in plastic serum separator vacutainers containing a silica clot accelerator, in which the blood samples were allowed to clot for 25-30 minutes at room temperature before centrifugation at 2500g for 10 minutes at 4°C (1). Packed red blood cells (for assay of HbA1c) were collected as whole blood into 9ml EDTA vacutainers, which were later separated by centrifugation at 2500g for 10 minutes at 4°C into plasma, buffy coat, and red cells (1).

For the assays, only the required tubes were extracted and thawed. Analyses of the serum biomarkers involved the use of 10 immunoassay analysers (6 x DiaSorin Liaison XL & 4x Beckman Coulter DXI 800) and 4 clinical analysers (2x Beckman Coulter AU5800 & 2x Siemens Advia 1800). The HbA1c assay was performed using five Bio-Rad Variate II Turbo analysers. The urinary assays were done on a single Beckman Coulter AU5400 clinical chemical analyser. Further details of the assays, reagents and calibrators used can be found in the relevant UK Biobank companion documents for serum biomarkers (3), HbA1c (4), and urinary biomarkers (5).

*Albumin-corrected calcium*

Total serum calcium includes its physiologically active form of ionized calcium, calcium bound to albumin, and a small proportion of calcium in a complex with various anions (6). In people with low albumin levels, total serum calcium levels may therefore appear artefactually low despite normal ionised calcium levels. To account for this, we additionally examined serum calcium levels corrected for serum albumin, using the modified Orrell method (6), calculated as:

Albumin-corrected calcium = serum calcium (mg/dL) + 0.8*(4-albumin [g/dL])

UK Biobank reports levels of calcium in mmol/L, and levels of albumin in g/L. Conversion factors used were 1 mmol/L=4.01 mg/dL for calcium and 10 g/L = 1g/dL for albumin.

**Supplemental Table 1**: Baseline characteristics^1^ of **white British women** by diet group in the UK Biobank.

| **Characteristics** | **Regular meat eaters (>3 times/week)**^2^ | **Low meat eaters (>3 times/week)**^2^ | **Poultry eaters** | **Fish eaters** | **Vegetarians** | **Vegans** |
| --- | --- | --- | --- | --- | --- | --- |
| Participants, n | 95,613 | 141,827 | 3,933 | 7,587 | 4,598 | 244 |
| Age, y | 56.7 ± 8.0 | 56.7 ± 7.9 | 56.6 ± 8.0 | 54.1 ± 8.0 | 52.9 ± 7.9 | 54.2 ± 8.1 |
| Fasting time, h | 3.7 ± 2.2 | 3.7 ± 2.3 | 3.7 ± 2.3 | 3.7 ± 2.3 | 3.8 ± 2.5 | 3.7 ± 2.2 |
| Body mass index, n (%) |  |  |  |  |  |  |
| <20 kg/m^2^ | 2,408 (2.5%) | 4,635 (3.3%) | 300 (7.6%) | 592 (7.8%) | 393 (8.5%) | 31 (12.7%) |
| 20-24.9 kg/m^2^ | 30,332 (31.7%) | 53,457 (37.7%) | 1,861 (47.3%) | 3,749 (49.4%) | 2,170 (47.2%) | 122 (50.0%) |
| 25-29.9 kg/m^2^ | 36,260 (37.9%) | 52,715 (37.2%) | 1,194 (30.4%) | 2,287 (30.1%) | 1,362 (29.6%) | 59 (24.2%) |
| ≥30 kg/m^2^ | 26,349 (27.6%) | 30,619 (21.6%) | 559 (14.2%) | 933 (12.3%) | 652 (14.2%) | 32 (13.1%) |
| Unknown | 264 (0.3%) | 401 (0.3%) | 19 (0.5%) | 26 (0.3%) | 21 (0.5%) | 0 (0.0%) |
| Alcohol consumption, n (%) |  |  |  |  |  |  |
| <1 g/day | 20,597 (21.5%) | 32,931 (23.2%) | 1,324 (33.7%) | 1,792 (23.6%) | 1,430 (31.1%) | 111 (45.5%) |
| 1-7 g/day | 29,506 (30.9%) | 47,549 (33.5%) | 1,220 (31.0%) | 2,392 (31.5%) | 1,416 (30.8%) | 70 (28.7%) |
| 8-15 g/day | 24,169 (25.3%) | 35,616 (25.1%) | 792 (20.1%) | 1,965 (25.9%) | 1,016 (22.1%) | 37 (15.2%) |
| ≥16 g/day | 21,288 (22.3%) | 25,654 (18.1%) | 595 (15.1%) | 1,435 (18.9%) | 733 (15.9%) | 26 (10.7%) |
| Unknown | 53 (0.1%) | 77 (0.1%) | 2 (0.1%) | 3 (0.04%) | 3 (0.1%) | 0 (0.0%) |
| Smoking, n (%) |  |  |  |  |  |  |
| Never | 55,778 (58.3%) | 83,071 (58.6%) | 2,263 (57.5%) | 4,277 (56.4%) | 2,763 (60.1%) | 143 (58.6%) |
| Previous | 30,003 (31.4%) | 46,336 (32.7%) | 1,360 (34.6%) | 2,773 (36.5%) | 1,503 (32.7%) | 85 (34.8%) |
| Current <15 cigarettes/day | 3,194 (3.3%) | 4,464 (3.1%) | 114 (2.9%) | 211 (2.8%) | 123 (2.7%) | 7 (2.9%) |
| Current ≥15 cigarettes/day | 6,281 (6.6%) | 7,435 (5.2%) | 179 (4.6%) | 300 (4.0%) | 195 (4.2%) | 9 (3.7%) |
| Unknown | 357 (0.4%) | 521 (0.4%) | 17 (0.4%) | 26 (0.3%) | 14 (0.3%) | 0 (0.0%) |
| Physical activity, n (%) |  |  |  |  |  |  |
| Low (<10 excess MET hours/week) | 18,253 (19.1%) | 25,909 (18.3%) | 566 (14.4%) | 1,200 (15.8%) | 797 (17.3%) | 42 (17.2%) |
| Moderate (10-49 excess MET hours/week) | 36,878 (38.6%) | 58,367 (41.2%) | 1,669 (42.4%) | 3,498 (46.1%) | 2,070 (45.0%) | 98 (40.2%) |
| High (≥50 excess MET hours/week) | 14,300 (15.0%) | 22,756 (16.0%) | 862 (21.9%) | 1,452 (19.1%) | 878 (19.1%) | 62 (25.4%) |
| Unknown | 26,182 (27.4%) | 34,795 (24.5%) | 836 (21.3%) | 1,437 (18.9%) | 853 (18.6%) | 42 (17.2%) |
| Lipid medication use, n (%) |  |  |  |  |  |  |
| No | 83,112 (86.9%) | 125,028 (88.2%) | 3,580 (91.0%) | 7,103 (93.6%) | 4,357 (94.8%) | 236 (96.7%) |
| Yes | 12,501 (13.1%) | 16,799 (11.8%) | 353 (9.0%) | 484 (6.4%) | 241 (5.2%) | 8 (3.3%) |
| Diabetes medication use, n (%) |  |  |  |  |  |  |
| No | 92,882 (97.1%) | 138,802 (97.9%) | 3,894 (99.0%) | 7,516 (99.1%) | 4,544 (98.8%) | 239 (98.0%) |
| Yes | 2,731 (2.9%) | 3,025 (2.1%) | 39 (1.0%) | 71 (0.9%) | 54 (1.2%) | 5 (2.0%) |

1. Values are means ± SD or frequency (percent).
2. Includes participants who consume any red or processed meat, regardless of whether they consume poultry, fish, or dairy. Cut-offs of regular and low consumption determined based on consumption of red and processed meat (beef, lamb, pork, processed meat) as reported on the touchscreen questionnaire.

**Supplemental Table 2**: Baseline characteristics^1^ of **white British men** by diet group in the UK Biobank.

| **Characteristics** | **Regular meat eaters (>3 times/week)**^2^ | **Low meat eaters (>3 times/week)**^2^ | **Poultry eaters** | **Fish eaters** | **Vegetarians** | **Vegans** |
| --- | --- | --- | --- | --- | --- | --- |
| Participants, n | 125,675 | 80,201 | 1,120 | 2,882 | 2,206 | 172 |
| Age, y | 56.9 ± 8.1 | 57.3 ± 8.0 | 57.1 ± 8.2 | 54.3 ± 8.0 | 52.7 ± 7.9 | 54.0 ± 7.7 |
| Fasting time, h | 3.9 ± 2.6 | 3.8 ± 2.4 | 4.0 ± 2.7 | 3.8 ± 2.5 | 3.6 ± 2.4 | 4.1 ± 3.0 |
| Body mass index, n (%) |  |  |  |  |  |  |
| <20 kg/m^2^ | 1,151 (0.9%) | 713 (0.9%) | 27 (2.4%) | 80 (2.8%) | 66 (3.0%) | 14 (8.1%) |
| 20-24.9 kg/m^2^ | 26,926 (21.4%) | 20,169 (25.1%) | 451 (40.3%) | 1,218 (42.3%) | 952 (43.2%) | 80 (46.5%) |
| 25-29.9 kg/m^2^ | 61,223 (48.7%) | 40,962 (51.1%) | 494 (44.1%) | 1,252 (43.4%) | 904 (41.0%) | 65 (37.8%) |
| ≥30 kg/m^2^ | 35,927 (28.6%) | 18,129 (22.6%) | 144 (12.9%) | 324 (11.2%) | 281 (12.7%) | 13 (7.6%) |
| Unknown | 448 (0.4%) | 228 (0.3%) | 4 (0.4%) | 8 (0.3%) | 3 (0.1%) | 0 (0.0%) |
| Alcohol consumption, n (%) |  |  |  |  |  |  |
| <1 g/day | 12,939 (10.3%) | 9,926 (12.4%) | 296 (26.4%) | 483 (16.8%) | 461 (20.9%) | 69 (40.1%) |
| 1-7 g/day | 19,920 (15.9%) | 15,891 (19.8%) | 239 (21.3%) | 545 (18.9%) | 470 (21.3%) | 30 (17.4%) |
| 8-15 g/day | 23,213 (18.5%) | 17,905 (22.3%) | 224 (20.0%) | 663 (23.0%) | 443 (20.1%) | 32 (18.6%) |
| ≥16 g/day | 69,502 (55.3%) | 36,440 (45.4%) | 361 (32.2%) | 1,191 (41.3%) | 830 (37.6%) | 41 (23.8%) |
| Unknown | 101 (0.1%) | 39 (0.05%) | 0 (0.0%) | 0 (0.0%) | 2 (0.1%) | 0 (0.0%) |
| Smoking, n (%) |  |  |  |  |  |  |
| Never | 58,342 (46.4%) | 40,708 (50.8%) | 635 (56.7%) | 1,551 (53.8%) | 1,190 (53.9%) | 77 (44.8%) |
| Previous | 49,365 (39.3%) | 31,450 (39.2%) | 396 (35.4%) | 1,090 (37.8%) | 791 (35.9%) | 77 (44.8%) |
| Current <15 cigarettes/day | 3,561 (2.8%) | 1,745 (2.2%) | 22 (2.0%) | 68 (2.4%) | 66 (3.0%) | 3 (1.7%) |
| Current ≥15 cigarettes/day | 13,866 (11.0%) | 5,961 (7.4%) | 65 (5.8%) | 167 (5.8%) | 150 (6.8%) | 14 (8.1%) |
| Unknown | 541 (0.4%) | 337 (0.4%) | 2 (0.2%) | 6 (0.2%) | 9 (0.4%) | 1 (0.6%) |
| Physical activity, n (%) |  |  |  |  |  |  |
| Low (<10 excess MET hours/week) | 26,094 (20.8%) | 15,596 (19.4%) | 156 (13.9%) | 434 (15.1%) | 428 (19.4%) | 25 (14.5%) |
| Moderate (10-49 excess MET hours/week) | 51,487 (41.0%) | 34,929 (43.6%) | 470 (42.0%) | 1,408 (48.9%) | 1,025 (46.5%) | 94 (54.7%) |
| High (≥50 excess MET hours/week) | 25,398 (20.2%) | 16,041 (20.0%) | 303 (27.1%) | 645 (22.4%) | 462 (20.9%) | 36 (20.9%) |
| Unknown | 22,696 (18.1%) | 13,635 (17.0%) | 191 (17.1%) | 395 (13.7%) | 291 (13.2%) | 17 (9.9%) |
| Lipid medication use, n (%) |  |  |  |  |  |  |
| No | 97,501 (77.6%) | 62,089 (77.4%) | 895 (79.9%) | 2,569 (89.1%) | 2,001 (90.7%) | 156 (90.7%) |
| Yes | 28,174 (22.4%) | 18,112 (22.6%) | 225 (20.1%) | 313 (10.9%) | 205 (9.3%) | 16 (9.3%) |
| Diabetes medication use, n (%) |  |  |  |  |  |  |
| No | 119,099 (94.8%) | 76,859 (95.8%) | 1,092 (97.5%) | 2,826 (98.1%) | 2,157 (97.8%) | 168 (97.7%) |
| Yes | 6,576 (5.2%) | 3,342 (4.2%) | 28 (2.5%) | 56 (1.9%) | 49 (2.2%) | 4 (2.3%) |

1. Values are means ± SD or frequency (percent).
2. Includes participants who consume any red or processed meat, regardless of whether they consume poultry, fish, or dairy. Cut-offs of regular and low consumption determined based on consumption of red and processed meat (beef, lamb, pork, processed meat) as reported on the touchscreen questionnaire.

**Supplemental Table 3**: Baseline characteristics^1^ of **British Indian women and men** by diet group in the UK Biobank.

| **Characteristics** | **Women** | |  | **Men** | |
| --- | --- | --- | --- | --- | --- |
|  | **Meat-eaters** | **Vegetarians** |  | **Meat-eaters** | **Vegetarians** |
| Participants, n | 1,743 | 936 |  | 2,348 | 508 |
| Age, y | 53.1 ± 8.1 | 54.6 ± 7.7 |  | 54.3 ± 8.6 | 55.7 ± 8.4 |
| Fasting time, h | 4.0 ± 2.3 | 4.1 ± 2.2 |  | 4.2 ± 2.5 | 4.1 ± 2.0 |
| Body mass index, n (%) |  |  |  |  |  |
| <20 kg/m^2^ | 47 (2.7%) | 32 (3.4%) |  | 32 (1.4%) | 15 (3.0%) |
| 20-24.9 kg/m^2^ | 572 (32.8%) | 266 (28.4%) |  | 714 (30.4%) | 176 (34.6%) |
| 25-29.9 kg/m^2^ | 704 (40.4%) | 414 (44.2%) |  | 1,146 (48.8%) | 229 (45.1%) |
| ≥30 kg/m^2^ | 409 (23.5%) | 220 (23.5%) |  | 441 (18.8%) | 80 (15.7%) |
| Unknown | 11 (0.6%) | 4 (0.4%) |  | 15 (0.6%) | 8 (1.6%) |
| Alcohol consumption, n (%) |  |  |  |  |  |
| <1 g/day | 1,070 (61.4%) | 841 (89.9%) |  | 632 (26.9%) | 368 (72.4%) |
| 1-7 g/day | 434 (24.9%) | 65 (6.9%) |  | 530 (22.6%) | 81 (15.9%) |
| 8-15 g/day | 158 (9.1%) | 18 (1.9%) |  | 443 (18.9%) | 37 (7.3%) |
| ≥16 g/day | 77 (4.4%) | 5 (0.5%) |  | 730 (31.1%) | 21 (4.1%) |
| Unknown | 4 (0.2%) | 7 (0.7%) |  | 13 (0.6%) | 1 (0.2%) |
| Smoking, n (%) |  |  |  |  |  |
| Never | 1,553 (89.1%) | 911 (97.3%) |  | 1,577 (67.2%) | 392 (77.2%) |
| Previous | 107 (6.1%) | 15 (1.6%) |  | 446 (19.0%) | 76 (15.0%) |
| Current <15 cigarettes/day | 35 (2.0%) | 0 (0.0%) |  | 117 (5.0%) | 10 (2.0%) |
| Current ≥15 cigarettes/day | 35 (2.0%) | 3 (0.3%) |  | 178 (7.6%) | 20 (3.9%) |
| Unknown | 13 (0.7%) | 7 (0.7%) |  | 30 (1.3%) | 10 (2.0%) |
| Physical activity, n (%) |  |  |  |  |  |
| Low (<10 excess MET hours/week) | 371 (21.3%) | 193 (20.6%) |  | 516 (22.0%) | 134 (26.4%) |
| Moderate (10-49 excess MET hours/week) | 602 (34.5%) | 304 (32.5%) |  | 891 (37.9%) | 187 (36.8%) |
| High (≥50 excess MET hours/week) | 206 (11.8%) | 82 (8.8%) |  | 346 (14.7%) | 54 (10.6%) |
| Unknown | 564 (32.4%) | 357 (38.1%) |  | 595 (25.3%) | 133 (26.2%) |
| Lipid medication use, n (%) |  |  |  |  |  |
| No | 1,426 (81.8%) | 760 (81.2%) |  | 1,602 (68.2%) | 370 (72.8%) |
| Yes | 317 (18.2%) | 176 (18.8%) |  | 746 (31.8%) | 138 (27.2%) |
| Diabetes medication use, n (%) |  |  |  |  |  |
| No | 1,571 (90.1%) | 833 (89.0%) |  | 1,988 (84.7%) | 427 (84.1%) |
| Yes | 172 (9.9%) | 103 (11.0%) |  | 360 (15.3%) | 81 (15.9%) |

1. Values are means ± SD or frequency (percent).

**Supplemental Table 4**: **Cardiovascular-related** serum biomarker concentrations (numbers, adjusted means and 95% CIs)^1^ in **white British participants** by diet group in the UK Biobank.

| **Cardiovascular-related serum biomarker** | **Regular meat eaters (>3 times/week)^2^** | **Low meat eaters (>3 times/week)^2^** | **Poultry eaters** | **Fish eaters** | **Vegetarians** | **Vegans** | **p-het^3^** |
| --- | --- | --- | --- | --- | --- | --- | --- |
| **Cholesterol, mmol/L** |  |  |  |  |  |  |  |
| Participants, n | 170,420 | 176,438 | 4,219 | 9,167 | 6,023 | 375 |  |
| Model 1 | 5.85 (5.85, 5.86) | 5.81 (5.81, 5.82) | 5.64 (5.61, 5.67) | 5.68 (5.66, 5.70) | 5.54 (5.52, 5.57) | 5.04 (4.95, 5.13) | <0.0001 |
| Model 2 | 5.85 (5.84, 5.85) | 5.81 (5.81, 5.82) | 5.68 (5.65, 5.71) | 5.72 (5.70, 5.74) | 5.59 (5.56, 5.61) | 5.10 (5.01, 5.19) | <0.0001 |
| Model 3 | 5.84 (5.84, 5.84)^a^ | 5.82 (5.81, 5.82)^b^ | 5.70 (5.67, 5.73)^c^ | 5.73 (5.71, 5.75)^c^ | 5.61 (5.58, 5.63)^d^ | 5.14 (5.05, 5.23)^e^ | <0.0001 |
| Model 4 | 5.84 (5.83, 5.84) | 5.82 (5.81, 5.82) | 5.70 (5.67, 5.73) | 5.73 (5.71, 5.75) | 5.61 (5.58, 5.63) | 5.14 (5.05, 5.23) | <0.0001 |
| Arithmetic mean | 5.94 (5.93, 5.94) | 5.91 (5.91, 5.92) | 5.80 (5.77, 5.83) | 5.83 (5.81, 5.85) | 5.70 (5.68, 5.73) | 5.26 (5.16, 5.37) | <0.0001 |
| **Direct low-density lipoprotein cholesterol, mmol/L** |  |  |  |  |  |  |  |
| Participants, n | 170,073 | 176,128 | 4,212 | 9,146 | 6,015 | 375 |  |
| Model 1 | 3.66 (3.66, 3.67) | 3.63 (3.62, 3.63) | 3.48 (3.46, 3.51) | 3.49 (3.48, 3.51) | 3.41 (3.39, 3.43) | 3.05 (2.98, 3.12) | <0.0001 |
| Model 2 | 3.65 (3.65, 3.65) | 3.63 (3.63, 3.64) | 3.54 (3.52, 3.57) | 3.55 (3.54, 3.57) | 3.47 (3.45, 3.49) | 3.13 (3.07, 3.20) | <0.0001 |
| Model 3 | 3.65 (3.65, 3.65)^a^ | 3.63 (3.63, 3.64)^b^ | 3.54 (3.52, 3.57)^c^ | 3.56 (3.54, 3.57)^c^ | 3.47 (3.45, 3.49)^d^ | 3.13 (3.07, 3.20)^e^ | <0.0001 |
| Model 4 | 3.65 (3.65, 3.65) | 3.63 (3.63, 3.64) | 3.54 (3.52, 3.57) | 3.56 (3.54, 3.57) | 3.47 (3.45, 3.49) | 3.14 (3.07, 3.20) | <0.0001 |
| Arithmetic mean | 3.74 (3.74, 3.74) | 3.72 (3.72, 3.72) | 3.63 (3.61, 3.66) | 3.65 (3.63, 3.66) | 3.56 (3.54, 3.58) | 3.25 (3.17, 3.33) | <0.0001 |
| **High-density lipoprotein cholesterol (HDL-C), mmol/L** |  |  |  |  |  |  |  |
| Participants, n | 156,154 | 161,180 | 3,816 | 8,328 | 5,458 | 346 |  |
| Model 1 | 1.42 (1.42, 1.43) | 1.44 (1.44, 1.44) | 1.46 (1.45, 1.47) | 1.49 (1.48, 1.50) | 1.42 (1.41, 1.43) | 1.35 (1.32, 1.38) | <0.0001 |
| Model 2 | 1.44 (1.44, 1.44) | 1.44 (1.44, 1.44) | 1.42 (1.41, 1.43) | 1.44 (1.43, 1.45) | 1.37 (1.37, 1.38) | 1.28 (1.25, 1.31) | <0.0001 |
| Model 3 | 1.43 (1.43, 1.43)^a^ | 1.44 (1.44, 1.44)^b^ | 1.44 (1.43, 1.45)^ab^ | 1.44 (1.44, 1.45)^b^ | 1.39 (1.38, 1.40)^c^ | 1.32 (1.30, 1.35)^d^ | <0.0001 |
| Model 4 | 1.43 (1.43, 1.43) | 1.44 (1.44, 1.44) | 1.43 (1.43, 1.44) | 1.44 (1.44, 1.45) | 1.39 (1.38, 1.40) | 1.32 (1.29, 1.35) | <0.0001 |
| Arithmetic mean | 1.48 (1.48, 1.48) | 1.49 (1.49, 1.49) | 1.49 (1.48, 1.50) | 1.49 (1.49, 1.50) | 1.43 (1.42, 1.44) | 1.37 (1.33, 1.40) | <0.0001 |
| **Triglycerides, mmol/L** |  |  |  |  |  |  |  |
| Participants, n | 170,264 | 176,323 | 4,214 | 9,156 | 6,017 | 375 |  |
| Model 1 | 1.54 (1.54, 1.55) | 1.45 (1.45, 1.46) | 1.32 (1.30, 1.34) | 1.34 (1.33, 1.36) | 1.51 (1.49, 1.53) | 1.36 (1.29, 1.43) | <0.0001 |
| Model 2 | 1.51 (1.51, 1.52) | 1.47 (1.46, 1.47) | 1.42 (1.40, 1.44) | 1.45 (1.44, 1.46) | 1.62 (1.60, 1.64) | 1.52 (1.45, 1.59) | <0.0001 |
| Model 3 | 1.51 (1.51, 1.52)^a^ | 1.47 (1.46, 1.47)^b^ | 1.41 (1.39, 1.43)^c^ | 1.45 (1.44, 1.47)^b^ | 1.62 (1.60, 1.64)^d^ | 1.50 (1.43, 1.57)^abc^ | <0.0001 |
| Model 4 | 1.51 (1.51, 1.52) | 1.47 (1.46, 1.47) | 1.42 (1.40, 1.44) | 1.46 (1.44, 1.47) | 1.62 (1.60, 1.64) | 1.50 (1.44, 1.58) | <0.0001 |
| Arithmetic mean | 1.74 (1.74, 1.75) | 1.68 (1.68, 1.69) | 1.63 (1.60, 1.66) | 1.67 (1.65, 1.69) | 1.84 (1.82, 1.87) | 1.75 (1.66, 1.85) | <0.0001 |
| **Apolipoprotein A1 (ApoA1), g/L** |  |  |  |  |  |  |  |
| Participants, n | 155,374 | 160,115 | 3,790 | 8,264 | 5,418 | 342 |  |
| Model 1 | 1.53 (1.53, 1.53) | 1.53 (1.53, 1.53) | 1.52 (1.52, 1.53) | 1.55 (1.54, 1.55) | 1.53 (1.52, 1.53) | 1.47 (1.45, 1.49) | <0.0001 |
| Model 2 | 1.54 (1.54, 1.54) | 1.53 (1.53, 1.53) | 1.50 (1.49, 1.50) | 1.52 (1.51, 1.52) | 1.50 (1.50, 1.51) | 1.43 (1.41, 1.45) | <0.0001 |
| Model 3 | 1.53 (1.53, 1.53)^a^ | 1.53 (1.53, 1.53)^a^ | 1.51 (1.51, 1.52)^b^ | 1.52 (1.52, 1.53)^b^ | 1.52 (1.51, 1.52)^b^ | 1.47 (1.44, 1.49)^c^ | <0.0001 |
| Model 4 | 1.53 (1.53, 1.53) | 1.53 (1.53, 1.53) | 1.51 (1.50, 1.52) | 1.52 (1.52, 1.53) | 1.51 (1.51, 1.52) | 1.46 (1.44, 1.49) | <0.0001 |
| Arithmetic mean | 1.55 (1.55, 1.56) | 1.55 (1.55, 1.55) | 1.54 (1.53, 1.54) | 1.54 (1.54, 1.55) | 1.54 (1.53, 1.54) | 1.49 (1.46, 1.51) | <0.0001 |
| **Apolipoprotein B (ApoB), g/L** |  |  |  |  |  |  |  |
| Participants, n | 169,474 | 175,709 | 4,208 | 9,125 | 5,993 | 372 |  |
| Model 1 | 1.05 (1.05, 1.05) | 1.04 (1.04, 1.04) | 1.00 (1.00, 1.01) | 1.00 (1.00, 1.01) | 0.98 (0.97, 0.98) | 0.91 (0.89, 0.93) | <0.0001 |
| Model 2 | 1.05 (1.05, 1.05) | 1.04 (1.04, 1.04) | 1.02 (1.02, 1.03) | 1.02 (1.02, 1.03) | 1.00 (0.99, 1.00) | 0.94 (0.92, 0.96) | <0.0001 |
| Model 3 | 1.05 (1.05, 1.05)^a^ | 1.04 (1.04, 1.04)^b^ | 1.02 (1.02, 1.03)^c^ | 1.02 (1.02, 1.03)^c^ | 1.00 (0.99, 1.00)^d^ | 0.94 (0.92, 0.96)^e^ | <0.0001 |
| Model 4 | 1.05 (1.05, 1.05) | 1.04 (1.04, 1.04) | 1.02 (1.02, 1.03) | 1.02 (1.02, 1.03) | 1.00 (0.99, 1.00) | 0.94 (0.92, 0.96) | <0.0001 |
| Arithmetic mean | 1.07 (1.07, 1.07) | 1.07 (1.07, 1.07) | 1.05 (1.04, 1.06) | 1.05 (1.05, 1.05) | 1.02 (1.02, 1.03) | 0.97 (0.95, 0.99) | <0.0001 |
| **C-reactive protein, mg/L** |  |  |  |  |  |  |  |
| Participants, n | 170,042 | 176,097 | 4,206 | 9,140 | 5,998 | 374 |  |
| Model 1 | 1.50 (1.49, 1.51) | 1.28 (1.28, 1.29) | 0.99 (0.96, 1.02) | 0.92 (0.90, 0.94) | 1.05 (1.02, 1.07) | 0.88 (0.79, 0.98) | <0.0001 |
| Model 2 | 1.43 (1.42, 1.44) | 1.32 (1.31, 1.32) | 1.18 (1.15, 1.21) | 1.11 (1.09, 1.13) | 1.24 (1.21, 1.27) | 1.16 (1.06, 1.28) | <0.0001 |
| Model 3 | 1.43 (1.42, 1.43)^a^ | 1.32 (1.31, 1.33)^b^ | 1.17 (1.14, 1.20)^c^ | 1.12 (1.09, 1.14)^c^ | 1.24 (1.21, 1.27)^d^ | 1.13 (1.03, 1.25)^cd^ | <0.0001 |
| Model 4 | 1.42 (1.42, 1.43) | 1.32 (1.32, 1.33) | 1.18 (1.15, 1.21) | 1.12 (1.10, 1.15) | 1.25 (1.22, 1.28) | 1.14 (1.04, 1.26) | <0.0001 |
| Arithmetic mean | 2.64 (2.62, 2.66) | 2.48 (2.46, 2.50) | 2.25 (2.13, 2.38) | 2.18 (2.09, 2.26) | 2.33 (2.23, 2.44) | 2.32 (1.90, 2.74) | <0.0001 |
| **Total and HDL-C ratio^4^** |  |  |  |  |  |  |  |
| Participants, n | 156,122 | 161,156 | 3,816 | 8,328 | 5,458 | 346 |  |
| Arithmetic mean, model 1 | 4.26 (4.25, 4.26) | 4.17 (4.17, 4.18) | 4.00 (3.97, 4.03) | 3.95 (3.93, 3.98) | 4.05 (4.02, 4.08) | 3.88 (3.77, 4.00) | <0.0001 |
| Arithmetic mean, model 2 | 4.22 (4.21, 4.22) | 4.19 (4.19, 4.20) | 4.16 (4.13, 4.19) | 4.12 (4.10, 4.15) | 4.21 (4.18, 4.23) | 4.14 (4.04, 4.25) | <0.0001 |
| Arithmetic mean, model 3 | 4.23 (4.23, 4.24)^a^ | 4.18 (4.18, 4.19)^b^ | 4.12 (4.09, 4.15)^c^ | 4.12 (4.10, 4.14)^c^ | 4.17 (4.15, 4.20)^bc^ | 4.04 (3.94, 4.15)^bc^ | <0.0001 |
| Arithmetic mean, model 4 | 4.23 (4.22, 4.23) | 4.18 (4.18, 4.19) | 4.13 (4.10, 4.16) | 4.12 (4.10, 4.15) | 4.18 (4.15, 4.20) | 4.05 (3.95, 4.15) | <0.0001 |
| **ApoB to ApoA1 ratio^5^** |  |  |  |  |  |  |  |
| Participants, n | 154,487 | 159,434 | 3,778 | 8,224 | 5,395 | 341 |  |
| Arithmetic mean, model 1 | 0.72 (0.71, 0.72) | 0.71 (0.71, 0.71) | 0.69 (0.68, 0.69) | 0.68 (0.67, 0.68) | 0.67 (0.66, 0.67) | 0.65 (0.62, 0.67) | <0.0001 |
| Arithmetic mean, model 2 | 0.71 (0.71, 0.71) | 0.71 (0.71, 0.71) | 0.71 (0.71, 0.72) | 0.70 (0.70, 0.71) | 0.69 (0.68, 0.69) | 0.68 (0.66, 0.70) | <0.0001 |
| Arithmetic mean, model 3 | 0.71 (0.71, 0.71)^a^ | 0.71 (0.71, 0.71)^b^ | 0.70 (0.70, 0.71)^abc^ | 0.70 (0.70, 0.71)^c^ | 0.68 (0.68, 0.69)^d^ | 0.67 (0.65, 0.69)^d^ | <0.0001 |
| Arithmetic mean, model 4 | 0.71 (0.71, 0.71) | 0.71 (0.71, 0.71) | 0.71 (0.70, 0.71) | 0.70 (0.70, 0.71) | 0.69 (0.68, 0.69) | 0.67 (0.65, 0.69) | <0.0001 |

1. Unless otherwise specified, all estimates represent adjusted geometric means and 95% CIs, or if stated, adjusted arithmetic means and 95% CIs. Model 1 was adjusted for sex, age (5 years categories) and fasting status (0-1, 2, 3, 4, 5, 6-7, ≥8 hours); model 2 was additionally adjusted for BMI (<20, 20.0-22.4, 22.5-24.9, 25.0-27.4, 27.5-29.9, 30.0-32.4, 32.5-34.9, ≥35.0 kg/m^2^, unknown), model 3 was additionally adjusted for alcohol consumption (<1, 1-7, 8-15, ≥16 g/d, unknown) and smoking status (never, previous, current <15 cigarettes/day, current ≥15 cigarettes/day, unknown), model 4 was additionally adjusted for physical activity (low <10 excess MET hours/week, moderate 10-49 excess MET hours/week, high ≥50 excess MET hours/week, unknown). Arithmetic means were based on model 3 unless otherwise specified. ﻿
2. Includes participants who consume any red or processed meat, regardless of whether they consume poultry, fish, or dairy. Cut-offs of regular and low consumption determined based on consumption of red and processed meat (beef, lamb, pork, processed meat) as reported on the touchscreen questionnaire.
3. Represents p for heterogeneity across the six diet groups based on Wald tests. Groups that do not share a superscript letter (ordered alphabetically from left to right) were significantly different at the 5% level from post hoc Bonferroni-corrected pairwise comparisons after linear regression (model 3).
4. Expressed as mmol/L of total cholesterol to mmol/L of HDL-C.
5. Expressed as g/L of ApoB to g/L of ApoA1.

**Supplemental Table 5**: **Cardiovascular-related** serum biomarker concentrations (numbers, adjusted means and 95% CIs)^1^ in **white British women** by diet group in the UK Biobank.

| **Cardiovascular-related serum biomarker** | **Regular meat eaters (>3 times/week)^2^** | **Low meat eaters (>3 times/week)^2^** | **Poultry eaters** | **Fish eaters** | **Vegetarians** | **Vegans** | **p-het^3^** |
| --- | --- | --- | --- | --- | --- | --- | --- |
| **Cholesterol, mmol/L** |  |  |  |  |  |  |  |
| Participants, n | 78,105 | 117,592 | 3,373 | 6,701 | 4,122 | 222 |  |
| Model 1 | 5.96 (5.95, 5.96) | 5.92 (5.92, 5.93) | 5.77 (5.74, 5.80) | 5.82 (5.80, 5.85) | 5.69 (5.67, 5.72) | 5.26 (5.14, 5.38) | <0.0001 |
| Model 2 | 5.95 (5.94, 5.96) | 5.93 (5.92, 5.93) | 5.81 (5.78, 5.84) | 5.86 (5.84, 5.88) | 5.73 (5.70, 5.76) | 5.31 (5.20, 5.43) | <0.0001 |
| Model 3 | 5.94 (5.94, 5.95)^a^ | 5.93 (5.92, 5.93)^b^ | 5.82 (5.79, 5.86)^c^ | 5.87 (5.84, 5.89)^c^ | 5.74 (5.71, 5.77)^d^ | 5.34 (5.22, 5.46)^e^ | <0.0001 |
| Model 4 | 5.94 (5.94, 5.95) | 5.93 (5.92, 5.93) | 5.82 (5.79, 5.86) | 5.87 (5.84, 5.89) | 5.74 (5.71, 5.77) | 5.34 (5.22, 5.46) | <0.0001 |
| Arithmetic mean | 6.04 (6.03, 6.05) | 6.02 (6.02, 6.03) | 5.92 (5.88, 5.95) | 5.97 (5.94, 5.99) | 5.84 (5.81, 5.88) | 5.46 (5.33, 5.59) | <0.0001 |
| **Direct low-density lipoprotein cholesterol, mmol/L** |  |  |  |  |  |  |  |
| Participants, n | 77,976 | 117,407 | 3,368 | 6,686 | 4,116 | 222 |  |
| Model 1 | 3.67 (3.67, 3.68) | 3.64 (3.64, 3.65) | 3.51 (3.49, 3.54) | 3.53 (3.51, 3.55) | 3.46 (3.43, 3.48) | 3.14 (3.05, 3.23) | <0.0001 |
| Model 2 | 3.66 (3.65, 3.66) | 3.65 (3.64, 3.65) | 3.57 (3.54, 3.59) | 3.58 (3.57, 3.60) | 3.51 (3.48, 3.53) | 3.22 (3.13, 3.31) | <0.0001 |
| Model 3 | 3.66 (3.65, 3.66)^a^ | 3.64 (3.64, 3.65)^b^ | 3.56 (3.54, 3.59)^c^ | 3.59 (3.57, 3.61)^c^ | 3.51 (3.48, 3.53)^d^ | 3.21 (3.12, 3.30)^e^ | <0.0001 |
| Model 4 | 3.66 (3.65, 3.66) | 3.64 (3.64, 3.65) | 3.57 (3.54, 3.59) | 3.59 (3.57, 3.61) | 3.51 (3.48, 3.53) | 3.21 (3.12, 3.30) | <0.0001 |
| Arithmetic mean | 3.75 (3.75, 3.76) | 3.74 (3.73, 3.74) | 3.66 (3.63, 3.68) | 3.68 (3.67, 3.70) | 3.60 (3.58, 3.63) | 3.32 (3.22, 3.42) | <0.0001 |
| **High-density lipoprotein cholesterol (HDL-C), mmol/L** |  |  |  |  |  |  |  |
| Participants, n | 70,852 | 106,896 | 3,043 | 6,053 | 3,706 | 208 |  |
| Model 1 | 1.56 (1.55, 1.56) | 1.58 (1.58, 1.58) | 1.61 (1.60, 1.63) | 1.63 (1.62, 1.64) | 1.56 (1.55, 1.57) | 1.49 (1.45, 1.54) | <0.0001 |
| Model 2 | 1.57 (1.57, 1.58) | 1.58 (1.58, 1.58) | 1.56 (1.55, 1.58) | 1.58 (1.57, 1.59) | 1.52 (1.51, 1.53) | 1.42 (1.38, 1.47) | <0.0001 |
| Model 3 | 1.57 (1.57, 1.57)^a^ | 1.58 (1.58, 1.58)^b^ | 1.58 (1.57, 1.60)^ab^ | 1.59 (1.58, 1.59)^b^ | 1.53 (1.52, 1.54)^c^ | 1.46 (1.42, 1.50)^d^ | <0.0001 |
| Model 4 | 1.57 (1.57, 1.57) | 1.58 (1.58, 1.58) | 1.58 (1.57, 1.59) | 1.58 (1.58, 1.59) | 1.53 (1.52, 1.54) | 1.46 (1.42, 1.50) | <0.0001 |
| Arithmetic mean | 1.61 (1.61, 1.61) | 1.62 (1.62, 1.62) | 1.63 (1.61, 1.64) | 1.63 (1.62, 1.64) | 1.57 (1.56, 1.58) | 1.50 (1.46, 1.55) | <0.0001 |
| **Triglycerides, mmol/L** |  |  |  |  |  |  |  |
| Participants, n | 78,061 | 117,534 | 3,368 | 6,693 | 4,118 | 222 |  |
| Model 1 | 1.40 (1.40, 1.40) | 1.32 (1.32, 1.33) | 1.20 (1.18, 1.22) | 1.24 (1.22, 1.25) | 1.40 (1.38, 1.42) | 1.27 (1.19, 1.35) | <0.0001 |
| Model 2 | 1.37 (1.37, 1.37) | 1.33 (1.33, 1.34) | 1.28 (1.26, 1.30) | 1.32 (1.31, 1.33) | 1.48 (1.46, 1.50) | 1.39 (1.31, 1.47) | <0.0001 |
| Model 3 | 1.37 (1.37, 1.38)^a^ | 1.33 (1.33, 1.33)^b^ | 1.27 (1.25, 1.29)^c^ | 1.32 (1.31, 1.34)^b^ | 1.47 (1.45, 1.49)^d^ | 1.37 (1.29, 1.45)^abcd^ | <0.0001 |
| Model 4 | 1.37 (1.37, 1.37) | 1.33 (1.33, 1.33) | 1.27 (1.26, 1.29) | 1.33 (1.31, 1.34) | 1.47 (1.45, 1.49) | 1.37 (1.30, 1.45) | <0.0001 |
| Arithmetic mean | 1.55 (1.55, 1.56) | 1.50 (1.50, 1.50) | 1.43 (1.40, 1.45) | 1.50 (1.48, 1.51) | 1.64 (1.62, 1.67) | 1.57 (1.47, 1.67) | <0.0001 |
| **Apolipoprotein A1 (ApoA1), g/L** |  |  |  |  |  |  |  |
| Participants, n | 70,213 | 105,906 | 3,017 | 5,993 | 3,668 | 204 |  |
| Model 1 | 1.62 (1.62, 1.62) | 1.62 (1.62, 1.62) | 1.62 (1.61, 1.63) | 1.64 (1.63, 1.65) | 1.62 (1.61, 1.63) | 1.56 (1.53, 1.60) | <0.0001 |
| Model 2 | 1.63 (1.63, 1.63) | 1.62 (1.62, 1.62) | 1.59 (1.58, 1.60) | 1.61 (1.61, 1.62) | 1.60 (1.59, 1.60) | 1.53 (1.49, 1.56) | <0.0001 |
| Model 3 | 1.62 (1.62, 1.62)^a^ | 1.62 (1.62, 1.62)^a^ | 1.61 (1.60, 1.62)^b^ | 1.61 (1.61, 1.62)^ab^ | 1.61 (1.60, 1.62)^b^ | 1.56 (1.53, 1.59)^c^ | <0.0001 |
| Model 4 | 1.62 (1.62, 1.62) | 1.62 (1.62, 1.62) | 1.61 (1.60, 1.61) | 1.61 (1.61, 1.62) | 1.61 (1.60, 1.62) | 1.56 (1.52, 1.59) | <0.0001 |
| Arithmetic mean | 1.64 (1.64, 1.65) | 1.64 (1.64, 1.64) | 1.63 (1.62, 1.64) | 1.64 (1.63, 1.64) | 1.63 (1.62, 1.64) | 1.58 (1.54, 1.61) | <0.0001 |
| **Apolipoprotein B (ApoB), g/L** |  |  |  |  |  |  |  |
| Participants, n | 77,804 | 117,231 | 3,367 | 6,671 | 4,108 | 221 |  |
| Model 1 | 1.04 (1.04, 1.05) | 1.03 (1.03, 1.04) | 1.00 (0.99, 1.01) | 1.00 (1.00, 1.01) | 0.98 (0.97, 0.99) | 0.92 (0.89, 0.95) | <0.0001 |
| Model 2 | 1.04 (1.04, 1.04) | 1.04 (1.03, 1.04) | 1.02 (1.01, 1.03) | 1.02 (1.02, 1.03) | 1.00 (0.99, 1.00) | 0.95 (0.92, 0.97) | <0.0001 |
| Model 3 | 1.04 (1.04, 1.04)^a^ | 1.04 (1.03, 1.04)^b^ | 1.02 (1.01, 1.03)^c^ | 1.02 (1.02, 1.03)^c^ | 1.00 (0.99, 1.00)^d^ | 0.94 (0.92, 0.97)^e^ | <0.0001 |
| Model 4 | 1.04 (1.04, 1.04) | 1.04 (1.03, 1.04) | 1.02 (1.01, 1.03) | 1.02 (1.02, 1.03) | 1.00 (0.99, 1.00) | 0.94 (0.92, 0.97) | <0.0001 |
| Arithmetic mean | 1.07 (1.06, 1.07) | 1.06 (1.06, 1.06) | 1.04 (1.04, 1.05) | 1.05 (1.04, 1.05) | 1.02 (1.02, 1.03) | 0.97 (0.95, 1.00) | <0.0001 |
| **C-reactive protein, mg/L** |  |  |  |  |  |  |  |
| Participants, n | 77,966 | 117,409 | 3,363 | 6,684 | 4,106 | 221 |  |
| Model 1 | 1.56 (1.55, 1.57) | 1.33 (1.32, 1.34) | 1.02 (0.98, 1.06) | 0.94 (0.92, 0.97) | 1.07 (1.04, 1.11) | 0.87 (0.76, 1.01) | <0.0001 |
| Model 2 | 1.46 (1.45, 1.47) | 1.36 (1.35, 1.37) | 1.23 (1.19, 1.27) | 1.15 (1.12, 1.17) | 1.26 (1.23, 1.30) | 1.14 (1.01, 1.29) | <0.0001 |
| Model 3 | 1.46 (1.45, 1.47)^a^ | 1.36 (1.35, 1.37)^b^ | 1.21 (1.18, 1.25)^cd^ | 1.15 (1.12, 1.17)^c^ | 1.25 (1.22, 1.29)^d^ | 1.11 (0.99, 1.26)^cd^ | <0.0001 |
| Model 4 | 1.46 (1.45, 1.47) | 1.36 (1.35, 1.37) | 1.22 (1.19, 1.26) | 1.16 (1.13, 1.18) | 1.26 (1.23, 1.30) | 1.12 (0.99, 1.27) | <0.0001 |
| Arithmetic mean | 2.74 (2.71, 2.77) | 2.58 (2.56, 2.61) | 2.39 (2.25, 2.53) | 2.27 (2.18, 2.37) | 2.45 (2.32, 2.57) | 2.20 (1.67, 2.74) | <0.0001 |
| **Total and HDL-C ratio^4^** |  |  |  |  |  |  |  |
| Participants, n | 70,837 | 106,878 | 3,043 | 6,053 | 3,706 | 208 |  |
| Arithmetic mean, model 1 | 3.95 (3.95, 3.96) | 3.86 (3.86, 3.87) | 3.69 (3.66, 3.73) | 3.68 (3.66, 3.71) | 3.77 (3.74, 3.80) | 3.66 (3.53, 3.80) | <0.0001 |
| Arithmetic mean, model 2 | 3.90 (3.90, 3.91) | 3.88 (3.87, 3.89) | 3.84 (3.80, 3.87) | 3.83 (3.80, 3.85) | 3.90 (3.87, 3.93) | 3.88 (3.75, 4.00) | <0.0001 |
| Arithmetic mean, model 3 | 3.92 (3.91, 3.92)^a^ | 3.87 (3.87, 3.88)^b^ | 3.80 (3.77, 3.83)^c^ | 3.83 (3.80, 3.85)^cd^ | 3.87 (3.84, 3.90)^bd^ | 3.80 (3.68, 3.92)^abcd^ | <0.0001 |
| Arithmetic mean, model 4 | 3.91 (3.91, 3.92) | 3.87 (3.87, 3.88) | 3.80 (3.77, 3.84) | 3.83 (3.81, 3.85) | 3.87 (3.85, 3.90) | 3.80 (3.68, 3.92) | <0.0001 |
| **ApoB to ApoA1 ratio^5^** |  |  |  |  |  |  |  |
| Participants, n | 69,928 | 105,567 | 3,011 | 5,965 | 3,658 | 204 |  |
| Arithmetic mean, model 1 | 0.67 (0.67, 0.67) | 0.66 (0.66, 0.66) | 0.64 (0.64, 0.65) | 0.64 (0.63, 0.64) | 0.63 (0.62, 0.63) | 0.61 (0.59, 0.64) | <0.0001 |
| Arithmetic mean, model 2 | 0.66 (0.66, 0.67) | 0.67 (0.66, 0.67) | 0.66 (0.66, 0.67) | 0.66 (0.65, 0.66) | 0.65 (0.64, 0.65) | 0.65 (0.62, 0.67) | <0.0001 |
| Arithmetic mean, model 3 | 0.67 (0.67, 0.67)^a^ | 0.66 (0.66, 0.67)^a^ | 0.66 (0.65, 0.66)^a^ | 0.66 (0.65, 0.66)^a^ | 0.64 (0.64, 0.65)^b^ | 0.63 (0.61, 0.66)^ab^ | <0.0001 |
| Arithmetic mean, model 4 | 0.67 (0.67, 0.67) | 0.66 (0.66, 0.67) | 0.66 (0.65, 0.67) | 0.66 (0.66, 0.66) | 0.64 (0.64, 0.65) | 0.63 (0.61, 0.66) | <0.0001 |

1. Unless otherwise specified, all estimates represent adjusted geometric means and 95% CIs, or if stated, adjusted arithmetic means and 95% CIs. Model 1 was adjusted for age (5 years categories) and fasting status (0-1, 2, 3, 4, 5, 6-7, ≥8 hours); model 2 was additionally adjusted for BMI (<20, 20.0-22.4, 22.5-24.9, 25.0-27.4, 27.5-29.9, 30.0-32.4, 32.5-34.9, ≥35.0 kg/m^2^, unknown), model 3 was additionally adjusted for alcohol consumption (<1, 1-7, 8-15, ≥16 g/d, unknown) and smoking status (never, previous, current <15 cigarettes/day, current ≥15 cigarettes/day, unknown), model 4 was additionally adjusted for physical activity (low <10 excess MET hours/week, moderate 10-49 excess MET hours/week, high ≥50 excess MET hours/week, unknown). Arithmetic means were based on model 3 unless otherwise specified.
2. Includes participants who consume any red or processed meat, regardless of whether they consume poultry, fish, or dairy. Cut-offs of regular and low consumption determined based on consumption of red and processed meat (beef, lamb, pork, processed meat) as reported on the touchscreen questionnaire.
3. Represents p for heterogeneity across the six diet groups based on Wald tests. Groups that do not share a superscript letter (ordered alphabetically from left to right) were significantly different at the 5% level from post hoc Bonferroni-corrected pairwise comparisons after linear regression (model 3).
4. Expressed as mmol/L of total cholesterol to mmol/L of HDL-C.
5. Expressed as g/L of ApoB to g/L of ApoA1.

**Supplemental Table 6**: **Cardiovascular-related** serum biomarker concentrations (numbers, adjusted means and 95% CIs)^1^ in **white British men** by diet group in the UK Biobank.

| **Cardiovascular-related serum biomarker** | **Regular meat eaters (>3 times/week)^2^** | **Low meat eaters (>3 times/week)^2^** | **Poultry eaters** | **Fish eaters** | **Vegetarians** | **Vegans** | **p-het^3^** |
| --- | --- | --- | --- | --- | --- | --- | --- |
| **Cholesterol, mmol/L** |  |  |  |  |  |  |  |
| Participants, n | 92,315 | 58,846 | 846 | 2,466 | 1,901 | 153 |  |
| Model 1 | 5.71 (5.70, 5.72) | 5.66 (5.65, 5.67) | 5.40 (5.33, 5.46) | 5.49 (5.45, 5.53) | 5.36 (5.32, 5.40) | 4.73 (4.60, 4.87) | <0.0001 |
| Model 2 | 5.71 (5.70, 5.72) | 5.66 (5.65, 5.67) | 5.44 (5.37, 5.50) | 5.53 (5.49, 5.57) | 5.40 (5.36, 5.45) | 4.80 (4.67, 4.94) | <0.0001 |
| Model 3 | 5.70 (5.69, 5.71)^a^ | 5.67 (5.66, 5.68)^b^ | 5.49 (5.42, 5.55)^cd^ | 5.55 (5.51, 5.59)^c^ | 5.44 (5.39, 5.48)^d^ | 4.88 (4.74, 5.01)^e^ | <0.0001 |
| Model 4 | 5.70 (5.69, 5.71) | 5.67 (5.66, 5.68) | 5.49 (5.42, 5.55) | 5.55 (5.51, 5.59) | 5.44 (5.39, 5.48) | 4.88 (4.74, 5.01) | <0.0001 |
| Arithmetic mean | 5.79 (5.78, 5.80) | 5.76 (5.75, 5.77) | 5.58 (5.52, 5.65) | 5.65 (5.61, 5.69) | 5.53 (5.48, 5.57) | 4.99 (4.83, 5.15) | <0.0001 |
| **Direct low-density lipoprotein cholesterol, mmol/L** |  |  |  |  |  |  |  |
| Participants, n | 92,097 | 58,721 | 844 | 2,460 | 1,899 | 153 |  |
| Model 1 | 3.64 (3.64, 3.65) | 3.61 (3.60, 3.62) | 3.41 (3.36, 3.46) | 3.46 (3.43, 3.49) | 3.37 (3.34, 3.40) | 2.92 (2.82, 3.02) | <0.0001 |
| Model 2 | 3.64 (3.63, 3.64) | 3.61 (3.61, 3.62) | 3.46 (3.41, 3.51) | 3.51 (3.48, 3.54) | 3.42 (3.39, 3.45) | 3.01 (2.91, 3.11) | <0.0001 |
| Model 3 | 3.63 (3.63, 3.64)^a^ | 3.62 (3.61, 3.62)^b^ | 3.47 (3.42, 3.52)^cd^ | 3.52 (3.49, 3.55)^c^ | 3.43 (3.40, 3.47)^d^ | 3.03 (2.93, 3.13)^e^ | <0.0001 |
| Model 4 | 3.63 (3.63, 3.64) | 3.62 (3.61, 3.62) | 3.48 (3.43, 3.53) | 3.52 (3.49, 3.55) | 3.43 (3.40, 3.47) | 3.03 (2.93, 3.13) | <0.0001 |
| Arithmetic mean | 3.72 (3.71, 3.72) | 3.70 (3.69, 3.70) | 3.56 (3.51, 3.61) | 3.61 (3.58, 3.64) | 3.51 (3.48, 3.55) | 3.14 (3.02, 3.26) | <0.0001 |
| **High-density lipoprotein cholesterol (HDL-C), mmol/L** |  |  |  |  |  |  |  |
| Participants, n | 85,302 | 54,284 | 773 | 2,275 | 1,752 | 138 |  |
| Model 1 | 1.27 (1.26, 1.27) | 1.28 (1.27, 1.28) | 1.27 (1.25, 1.29) | 1.32 (1.31, 1.33) | 1.25 (1.24, 1.27) | 1.18 (1.14, 1.23) | <0.0001 |
| Model 2 | 1.27 (1.27, 1.27) | 1.27 (1.27, 1.27) | 1.23 (1.21, 1.25) | 1.27 (1.26, 1.28) | 1.20 (1.19, 1.21) | 1.11 (1.07, 1.15) | <0.0001 |
| Model 3 | 1.27 (1.26, 1.27)^a^ | 1.28 (1.27, 1.28)^b^ | 1.26 (1.24, 1.28)^ab^ | 1.28 (1.27, 1.29)^ab^ | 1.22 (1.21, 1.23)^c^ | 1.15 (1.11, 1.19)^d^ | <0.0001 |
| Model 4 | 1.27 (1.27, 1.27) | 1.28 (1.27, 1.28) | 1.25 (1.24, 1.27) | 1.28 (1.26, 1.29) | 1.22 (1.21, 1.23) | 1.15 (1.11, 1.19) | <0.0001 |
| Arithmetic mean | 1.30 (1.30, 1.30) | 1.31 (1.31, 1.31) | 1.30 (1.28, 1.32) | 1.31 (1.30, 1.32) | 1.25 (1.24, 1.26) | 1.18 (1.13, 1.22) | <0.0001 |
| **Triglycerides, mmol/L** |  |  |  |  |  |  |  |
| Participants, n | 92,203 | 58,789 | 846 | 2,463 | 1,899 | 153 |  |
| Model 1 | 1.75 (1.75, 1.76) | 1.65 (1.64, 1.65) | 1.49 (1.44, 1.55) | 1.51 (1.48, 1.54) | 1.70 (1.66, 1.74) | 1.48 (1.36, 1.61) | <0.0001 |
| Model 2 | 1.73 (1.72, 1.74) | 1.67 (1.66, 1.67) | 1.63 (1.57, 1.68) | 1.65 (1.62, 1.69) | 1.86 (1.82, 1.91) | 1.73 (1.60, 1.87) | <0.0001 |
| Model 3 | 1.73 (1.72, 1.73)^a^ | 1.67 (1.66, 1.67)^b^ | 1.62 (1.57, 1.68)^b^ | 1.66 (1.63, 1.69)^b^ | 1.86 (1.82, 1.90)^c^ | 1.71 (1.58, 1.85)^abc^ | <0.0001 |
| Model 4 | 1.73 (1.72, 1.73) | 1.67 (1.66, 1.67) | 1.64 (1.58, 1.69) | 1.66 (1.63, 1.69) | 1.86 (1.82, 1.91) | 1.72 (1.59, 1.85) | <0.0001 |
| Arithmetic mean | 1.99 (1.99, 2.00) | 1.92 (1.91, 1.93) | 1.90 (1.83, 1.97) | 1.92 (1.88, 1.96) | 2.15 (2.10, 2.20) | 2.02 (1.85, 2.19) | <0.0001 |
| **Apolipoprotein A1 (ApoA1), g/L** |  |  |  |  |  |  |  |
| Participants, n | 85,161 | 54,209 | 773 | 2,271 | 1,750 | 138 |  |
| Model 1 | 1.42 (1.42, 1.42) | 1.42 (1.42, 1.42) | 1.39 (1.37, 1.40) | 1.43 (1.42, 1.44) | 1.41 (1.40, 1.42) | 1.36 (1.32, 1.39) | <0.0001 |
| Model 2 | 1.43 (1.42, 1.43) | 1.41 (1.41, 1.42) | 1.36 (1.35, 1.38) | 1.40 (1.39, 1.41) | 1.38 (1.37, 1.39) | 1.31 (1.28, 1.35) | <0.0001 |
| Model 3 | 1.42 (1.42, 1.42)^a^ | 1.42 (1.42, 1.42)^a^ | 1.39 (1.37, 1.40)^bc^ | 1.41 (1.40, 1.42)^ab^ | 1.40 (1.39, 1.41)^bc^ | 1.35 (1.32, 1.38)^c^ | <0.0001 |
| Model 4 | 1.42 (1.42, 1.42) | 1.42 (1.42, 1.42) | 1.39 (1.37, 1.40) | 1.41 (1.40, 1.41) | 1.40 (1.39, 1.41) | 1.35 (1.32, 1.38) | <0.0001 |
| Arithmetic mean | 1.44 (1.44, 1.44) | 1.44 (1.44, 1.44) | 1.41 (1.39, 1.42) | 1.42 (1.42, 1.43) | 1.42 (1.41, 1.43) | 1.37 (1.33, 1.40) | <0.0001 |
| **Apolipoprotein B (ApoB), g/L** |  |  |  |  |  |  |  |
| Participants, n | 91,670 | 58,478 | 841 | 2,454 | 1,885 | 151 |  |
| Model 1 | 1.06 (1.06, 1.06) | 1.05 (1.05, 1.05) | 1.00 (0.99, 1.01) | 1.01 (1.00, 1.02) | 0.98 (0.97, 0.99) | 0.89 (0.86, 0.92) | <0.0001 |
| Model 2 | 1.06 (1.06, 1.06) | 1.05 (1.05, 1.05) | 1.02 (1.00, 1.03) | 1.03 (1.02, 1.04) | 1.00 (0.99, 1.01) | 0.93 (0.89, 0.96) | <0.0001 |
| Model 3 | 1.06 (1.05, 1.06)^a^ | 1.05 (1.05, 1.05)^b^ | 1.02 (1.01, 1.04)^cd^ | 1.03 (1.02, 1.04)^c^ | 1.00 (0.99, 1.01)^d^ | 0.93 (0.90, 0.96)^e^ | <0.0001 |
| Model 4 | 1.06 (1.05, 1.06) | 1.05 (1.05, 1.05) | 1.02 (1.01, 1.04) | 1.03 (1.02, 1.04) | 1.00 (0.99, 1.01) | 0.93 (0.90, 0.96) | <0.0001 |
| Arithmetic mean | 1.08 (1.08, 1.08) | 1.07 (1.07, 1.08) | 1.05 (1.03, 1.06) | 1.05 (1.05, 1.06) | 1.02 (1.01, 1.03) | 0.96 (0.93, 1.00) | <0.0001 |
| **C-reactive protein, mg/L** |  |  |  |  |  |  |  |
| Participants, n | 92,076 | 58,688 | 843 | 2,456 | 1,892 | 153 |  |
| Model 1 | 1.42 (1.41, 1.43) | 1.22 (1.21, 1.23) | 0.96 (0.89, 1.02) | 0.90 (0.87, 0.94) | 1.03 (0.99, 1.08) | 0.89 (0.76, 1.05) | <0.0001 |
| Model 2 | 1.39 (1.38, 1.40) | 1.25 (1.24, 1.26) | 1.11 (1.04, 1.19) | 1.07 (1.03, 1.11) | 1.22 (1.17, 1.28) | 1.16 (1.00, 1.35) | <0.0001 |
| Model 3 | 1.38 (1.37, 1.39)^a^ | 1.27 (1.26, 1.28)^b^ | 1.12 (1.05, 1.19)^cd^ | 1.08 (1.04, 1.12)^c^ | 1.23 (1.18, 1.28)^bd^ | 1.13 (0.98, 1.32)^abcd^ | <0.0001 |
| Model 4 | 1.38 (1.37, 1.39) | 1.27 (1.26, 1.28) | 1.13 (1.06, 1.21) | 1.09 (1.05, 1.13) | 1.23 (1.18, 1.29) | 1.15 (0.99, 1.33) | <0.0001 |
| Arithmetic mean | 2.51 (2.48, 2.54) | 2.34 (2.30, 2.37) | 2.10 (1.81, 2.38) | 2.08 (1.91, 2.25) | 2.14 (1.95, 2.33) | 2.42 (1.75, 3.09) | <0.0001 |
| **Total and HDL-C ratio^4^** |  |  |  |  |  |  |  |
| Participants, n | 85,285 | 54,278 | 773 | 2,275 | 1,752 | 138 |  |
| Arithmetic mean, model 1 | 4.66 (4.65, 4.67) | 4.58 (4.57, 4.59) | 4.41 (4.33, 4.49) | 4.31 (4.26, 4.35) | 4.42 (4.37, 4.47) | 4.15 (3.96, 4.34) | <0.0001 |
| Arithmetic mean, model 2 | 4.63 (4.63, 4.64) | 4.60 (4.59, 4.61) | 4.59 (4.51, 4.67) | 4.51 (4.47, 4.56) | 4.63 (4.58, 4.68) | 4.49 (4.31, 4.67) | <0.0001 |
| Arithmetic mean, model 3 | 4.64 (4.64, 4.65)^a^ | 4.59 (4.58, 4.60)^b^ | 4.52 (4.45, 4.59)^bc^ | 4.49 (4.45, 4.54)^c^ | 4.59 (4.54, 4.64)^abc^ | 4.38 (4.20, 4.56)^bc^ | <0.0001 |
| Arithmetic mean, model 4 | 4.64 (4.64, 4.65) | 4.59 (4.58, 4.60) | 4.54 (4.46, 4.61) | 4.50 (4.46, 4.54) | 4.59 (4.54, 4.64) | 4.39 (4.21, 4.56) | <0.0001 |
| **ApoB to ApoA1 ratio^5^** |  |  |  |  |  |  |  |
| Participants, n | 84,559 | 53,867 | 767 | 2,259 | 1,737 | 137 |  |
| Arithmetic mean, model 1 | 0.77 (0.77, 0.77) | 0.77 (0.76, 0.77) | 0.75 (0.74, 0.76) | 0.73 (0.72, 0.74) | 0.72 (0.71, 0.73) | 0.68 (0.65, 0.72) | <0.0001 |
| Arithmetic mean, model 2 | 0.77 (0.77, 0.77) | 0.77 (0.77, 0.77) | 0.78 (0.76, 0.79) | 0.76 (0.75, 0.77) | 0.75 (0.74, 0.75) | 0.73 (0.70, 0.76) | <0.0001 |
| Arithmetic mean, model 3 | 0.77 (0.77, 0.77)^a^ | 0.77 (0.77, 0.77)^b^ | 0.77 (0.75, 0.78)^abc^ | 0.76 (0.75, 0.77)^bcd^ | 0.74 (0.73, 0.75)^d^ | 0.72 (0.68, 0.75)^cd^ | <0.0001 |
| Arithmetic mean, model 4 | 0.77 (0.77, 0.77) | 0.77 (0.77, 0.77) | 0.77 (0.75, 0.78) | 0.76 (0.75, 0.77) | 0.74 (0.73, 0.75) | 0.72 (0.69, 0.75) | <0.0001 |

1. Unless otherwise specified, all estimates represent adjusted geometric means and 95% CIs, or if stated, adjusted arithmetic means and 95% CIs. Model 1 was adjusted for age (5 years categories) and fasting status (0-1, 2, 3, 4, 5, 6-7, ≥8 hours); model 2 was additionally adjusted for BMI (<20, 20.0-22.4, 22.5-24.9, 25.0-27.4, 27.5-29.9, 30.0-32.4, 32.5-34.9, ≥35.0 kg/m^2^, unknown), model 3 was additionally adjusted for alcohol consumption (<1, 1-7, 8-15, ≥16 g/d, unknown) and smoking status (never, previous, current <15 cigarettes/day, current ≥15 cigarettes/day, unknown), model 4 was additionally adjusted for physical activity (low <10 excess MET hours/week, moderate 10-49 excess MET hours/week, high ≥50 excess MET hours/week, unknown). Arithmetic means were based on model 3 unless otherwise specified.
2. Includes participants who consume any red or processed meat, regardless of whether they consume poultry, fish, or dairy. Cut-offs of regular and low consumption determined based on consumption of red and processed meat (beef, lamb, pork, processed meat) as reported on the touchscreen questionnaire.
3. Represents p for heterogeneity across the six diet groups based on Wald tests. ﻿ Groups that do not share a superscript letter (ordered alphabetically from left to right) were significantly different at the 5% level from post hoc Bonferroni-corrected pairwise comparisons after linear regression (model 3).
4. Expressed as mmol/L of total cholesterol to mmol/L of HDL-C.
5. Expressed as g/L of ApoB to g/L of ApoA1.

**Supplemental Table 7**: **Cardiovascular-related** serum biomarker concentrations (numbers, adjusted means and 95% CIs)^1^ in **British Indian participants** by diet group in the UK Biobank.

| **Cardiovascular-related serum biomarker** | **All participants** | | |  | **Women** | | |  | **Men** | | |
| --- | --- | --- | --- | --- | --- | --- | --- | --- | --- | --- | --- |
|  | **Meat-eaters** | **Vegetarians** | **p-het^2^** |  | **Meat-eaters** | **Vegetarians** | **p-het^2^** |  | **Meat-eaters** | **Vegetarians** | **p-het^2^** |
| **Cholesterol, mmol/L** |  |  |  |  |  |  |  |  |  |  |  |
| Participants, n | 2,832 | 1,057 |  |  | 1,314 | 710 |  |  | 1,518 | 347 |  |
| Model 1 | 5.58 (5.54, 5.62) | 5.34 (5.28, 5.40) | <0.0001 |  | 5.63 (5.58, 5.69) | 5.39 (5.32, 5.46) | <0.0001 |  | 5.53 (5.48, 5.58) | 5.28 (5.17, 5.38) | <0.0001 |
| Model 2 | 5.58 (5.54, 5.62) | 5.34 (5.29, 5.40) | <0.0001 |  | 5.63 (5.58, 5.69) | 5.39 (5.32, 5.46) | <0.0001 |  | 5.53 (5.48, 5.58) | 5.28 (5.18, 5.38) | <0.0001 |
| Model 3 | 5.57 (5.53, 5.61) | 5.37 (5.31, 5.43) | <0.0001 |  | 5.62 (5.57, 5.68) | 5.41 (5.34, 5.48) | <0.0001 |  | 5.51 (5.46, 5.57) | 5.33 (5.22, 5.45) | 0.005 |
| Model 4 | 5.57 (5.53, 5.61) | 5.37 (5.31, 5.43) | <0.0001 |  | 5.62 (5.57, 5.67) | 5.41 (5.34, 5.48) | <0.0001 |  | 5.51 (5.46, 5.57) | 5.33 (5.22, 5.45) | 0.005 |
| Arithmetic mean | 5.66 (5.63, 5.70) | 5.45 (5.38, 5.51) | <0.0001 |  | 5.71 (5.66, 5.76) | 5.49 (5.42, 5.56) | <0.0001 |  | 5.61 (5.56, 5.66) | 5.41 (5.30, 5.52) | 0.002 |
| **Direct low-density lipoprotein cholesterol, mmol/L** |  |  |  |  |  |  |  |  |  |  |  |
| Participants, n | 2,833 | 1,056 |  |  | 1,314 | 710 |  |  | 1,519 | 346 |  |
| Model 1 | 3.52 (3.50, 3.55) | 3.38 (3.34, 3.43) | <0.0001 |  | 3.52 (3.48, 3.56) | 3.36 (3.31, 3.41) | <0.0001 |  | 3.53 (3.49, 3.57) | 3.41 (3.33, 3.49) | 0.008 |
| Model 2 | 3.52 (3.49, 3.55) | 3.39 (3.34, 3.43) | <0.0001 |  | 3.52 (3.48, 3.56) | 3.36 (3.31, 3.41) | <0.0001 |  | 3.53 (3.49, 3.57) | 3.42 (3.34, 3.50) | 0.01 |
| Model 3 | 3.52 (3.50, 3.55) | 3.38 (3.34, 3.43) | <0.0001 |  | 3.52 (3.48, 3.56) | 3.36 (3.31, 3.41) | <0.0001 |  | 3.53 (3.49, 3.57) | 3.42 (3.34, 3.51) | 0.03 |
| Model 4 | 3.52 (3.50, 3.55) | 3.38 (3.34, 3.43) | <0.0001 |  | 3.52 (3.48, 3.56) | 3.36 (3.31, 3.41) | <0.0001 |  | 3.53 (3.49, 3.57) | 3.42 (3.34, 3.51) | 0.03 |
| Arithmetic mean | 3.61 (3.58, 3.64) | 3.45 (3.40, 3.50) | <0.0001 |  | 3.60 (3.56, 3.64) | 3.43 (3.38, 3.49) | <0.0001 |  | 3.62 (3.58, 3.65) | 3.48 (3.40, 3.57) | 0.007 |
| **High-density lipoprotein cholesterol (HDL-C), mmol/L** |  |  |  |  |  |  |  |  |  |  |  |
| Participants, n | 2,585 | 945 |  |  | 1,185 | 632 |  |  | 1,400 | 313 |  |
| Model 1 | 1.29 (1.28, 1.30) | 1.21 (1.19, 1.23) | <0.0001 |  | 1.40 (1.38, 1.41) | 1.33 (1.31, 1.36) | <0.0001 |  | 1.19 (1.18, 1.20) | 1.08 (1.05, 1.11) | <0.0001 |
| Model 2 | 1.29 (1.28, 1.30) | 1.21 (1.19, 1.23) | <0.0001 |  | 1.40 (1.38, 1.41) | 1.33 (1.31, 1.36) | <0.0001 |  | 1.19 (1.18, 1.20) | 1.08 (1.05, 1.10) | <0.0001 |
| Model 3 | 1.28 (1.27, 1.29) | 1.25 (1.23, 1.27) | 0.005 |  | 1.38 (1.37, 1.40) | 1.36 (1.33, 1.38) | 0.06 |  | 1.18 (1.16, 1.19) | 1.14 (1.11, 1.16) | 0.02 |
| Model 4 | 1.28 (1.27, 1.29) | 1.25 (1.23, 1.27) | 0.007 |  | 1.38 (1.37, 1.40) | 1.36 (1.33, 1.38) | 0.07 |  | 1.18 (1.16, 1.19) | 1.14 (1.11, 1.17) | 0.02 |
| Arithmetic mean | 1.32 (1.31, 1.33) | 1.28 (1.26, 1.30) | 0.002 |  | 1.42 (1.40, 1.44) | 1.39 (1.37, 1.41) | 0.04 |  | 1.21 (1.19, 1.22) | 1.16 (1.13, 1.19) | 0.008 |
| **Triglycerides, mmol/L** |  |  |  |  |  |  |  |  |  |  |  |
| Participants, n | 2,833 | 1,056 |  |  | 1,314 | 710 |  |  | 1,519 | 346 |  |
| Model 1 | 1.64 (1.61, 1.68) | 1.74 (1.68, 1.79) | 0.003 |  | 1.49 (1.45, 1.53) | 1.56 (1.51, 1.61) | 0.03 |  | 1.84 (1.79, 1.89) | 1.95 (1.85, 2.07) | 0.05 |
| Model 2 | 1.64 (1.61, 1.67) | 1.75 (1.70, 1.80) | 0.001 |  | 1.49 (1.45, 1.52) | 1.56 (1.51, 1.61) | 0.02 |  | 1.83 (1.79, 1.88) | 1.98 (1.87, 2.09) | 0.01 |
| Model 3 | 1.65 (1.62, 1.69) | 1.71 (1.66, 1.76) | 0.09 |  | 1.49 (1.46, 1.53) | 1.55 (1.49, 1.60) | 0.12 |  | 1.85 (1.80, 1.89) | 1.91 (1.80, 2.03) | 0.30 |
| Model 4 | 1.66 (1.63, 1.69) | 1.71 (1.65, 1.76) | 0.11 |  | 1.49 (1.46, 1.53) | 1.55 (1.49, 1.60) | 0.12 |  | 1.85 (1.80, 1.90) | 1.91 (1.80, 2.02) | 0.35 |
| Arithmetic mean | 1.91 (1.87, 1.95) | 1.93 (1.86, 2.00) | 0.61 |  | 1.70 (1.65, 1.75) | 1.72 (1.66, 1.79) | 0.60 |  | 2.14 (2.08, 2.20) | 2.17 (2.03, 2.31) | 0.69 |
| **Apolipoprotein A1 (ApoA1), g/L** |  |  |  |  |  |  |  |  |  |  |  |
| Participants, n | 2,574 | 944 |  |  | 1,178 | 631 |  |  | 1,396 | 313 |  |
| Model 1 | 1.42 (1.41, 1.43) | 1.37 (1.36, 1.38) | <0.0001 |  | 1.50 (1.48, 1.51) | 1.46 (1.44, 1.48) | 0.002 |  | 1.35 (1.34, 1.36) | 1.27 (1.25, 1.29) | <0.0001 |
| Model 2 | 1.42 (1.42, 1.43) | 1.37 (1.36, 1.38) | <0.0001 |  | 1.50 (1.48, 1.51) | 1.46 (1.44, 1.48) | 0.001 |  | 1.35 (1.34, 1.36) | 1.27 (1.25, 1.29) | <0.0001 |
| Model 3 | 1.41 (1.40, 1.42) | 1.40 (1.39, 1.41) | 0.13 |  | 1.49 (1.47, 1.50) | 1.48 (1.46, 1.49) | 0.36 |  | 1.34 (1.33, 1.35) | 1.32 (1.30, 1.34) | 0.14 |
| Model 4 | 1.41 (1.40, 1.42) | 1.40 (1.39, 1.41) | 0.19 |  | 1.49 (1.47, 1.50) | 1.48 (1.46, 1.50) | 0.43 |  | 1.34 (1.33, 1.35) | 1.32 (1.30, 1.34) | 0.20 |
| Arithmetic mean | 1.43 (1.42, 1.44) | 1.42 (1.40, 1.43) | 0.09 |  | 1.51 (1.49, 1.52) | 1.49 (1.48, 1.51) | 0.31 |  | 1.35 (1.34, 1.36) | 1.33 (1.31, 1.36) | 0.10 |
| **Apolipoprotein B (ApoB), g/L** |  |  |  |  |  |  |  |  |  |  |  |
| Participants, n | 2,808 | 1,052 |  |  | 1,307 | 707 |  |  | 1,501 | 345 |  |
| Model 1 | 1.03 (1.03, 1.04) | 1.00 (0.98, 1.01) | <0.0001 |  | 1.02 (1.01, 1.03) | 0.98 (0.96, 0.99) | <0.0001 |  | 1.05 (1.04, 1.06) | 1.02 (1.00, 1.04) | 0.01 |
| Model 2 | 1.03 (1.03, 1.04) | 1.00 (0.99, 1.01) | <0.0001 |  | 1.02 (1.01, 1.03) | 0.98 (0.96, 0.99) | <0.0001 |  | 1.05 (1.04, 1.06) | 1.02 (1.00, 1.05) | 0.03 |
| Model 3 | 1.04 (1.03, 1.04) | 1.00 (0.98, 1.01) | <0.0001 |  | 1.02 (1.01, 1.03) | 0.98 (0.96, 0.99) | <0.0001 |  | 1.05 (1.04, 1.06) | 1.02 (1.00, 1.05) | 0.03 |
| Model 4 | 1.04 (1.03, 1.04) | 1.00 (0.98, 1.01) | <0.0001 |  | 1.02 (1.01, 1.03) | 0.98 (0.96, 0.99) | <0.0001 |  | 1.05 (1.04, 1.06) | 1.02 (1.00, 1.05) | 0.03 |
| Arithmetic mean | 1.06 (1.05, 1.07) | 1.02 (1.00, 1.03) | <0.0001 |  | 1.04 (1.03, 1.05) | 1.00 (0.98, 1.01) | <0.0001 |  | 1.07 (1.06, 1.08) | 1.04 (1.02, 1.06) | 0.01 |
| **C-reactive protein, mg/L** |  |  |  |  |  |  |  |  |  |  |  |
| Participants, n | 2,821 | 1,055 |  |  | 1,309 | 708 |  |  | 1,512 | 347 |  |
| Model 1 | 1.67 (1.60, 1.73) | 1.66 (1.56, 1.76) | 0.88 |  | 1.91 (1.81, 2.03) | 1.95 (1.81, 2.11) | 0.66 |  | 1.43 (1.36, 1.50) | 1.36 (1.23, 1.51) | 0.38 |
| Model 2 | 1.66 (1.60, 1.72) | 1.67 (1.58, 1.77) | 0.80 |  | 1.91 (1.82, 2.01) | 1.96 (1.83, 2.10) | 0.55 |  | 1.43 (1.36, 1.49) | 1.38 (1.26, 1.52) | 0.57 |
| Model 3 | 1.68 (1.62, 1.73) | 1.63 (1.54, 1.73) | 0.49 |  | 1.92 (1.83, 2.02) | 1.94 (1.80, 2.08) | 0.89 |  | 1.44 (1.38, 1.51) | 1.33 (1.20, 1.47) | 0.16 |
| Model 4 | 1.68 (1.62, 1.73) | 1.63 (1.54, 1.73) | 0.49 |  | 1.92 (1.83, 2.02) | 1.94 (1.80, 2.08) | 0.89 |  | 1.44 (1.38, 1.51) | 1.33 (1.20, 1.47) | 0.17 |
| Arithmetic mean | 2.95 (2.80, 3.09) | 2.72 (2.47, 2.97) | 0.13 |  | 3.42 (3.19, 3.65) | 3.25 (2.93, 3.57) | 0.42 |  | 2.42 (2.25, 2.60) | 2.13 (1.74, 2.52) | 0.19 |
| **Total and HDL-C ratio^3^** |  |  |  |  |  |  |  |  |  |  |  |
| Participants, n | 2,583 | 945 |  |  | 1,184 | 632 |  |  | 1,399 | 313 |  |
| Arithmetic mean, model 1 | 4.46 (4.42, 4.50) | 4.53 (4.46, 4.60) | 0.11 |  | 4.16 (4.10, 4.22) | 4.13 (4.06, 4.21) | 0.60 |  | 4.79 (4.73, 4.85) | 4.98 (4.86, 5.11) | 0.007 |
| Arithmetic mean, model 2 | 4.46 (4.42, 4.50) | 4.53 (4.47, 4.60) | 0.06 |  | 4.16 (4.10, 4.21) | 4.14 (4.06, 4.21) | 0.68 |  | 4.79 (4.73, 4.85) | 5.00 (4.88, 5.12) | 0.002 |
| Arithmetic mean, model 3 | 4.50 (4.46, 4.54) | 4.42 (4.35, 4.48) | 0.04 |  | 4.19 (4.13, 4.24) | 4.08 (4.01, 4.16) | 0.04 |  | 4.84 (4.78, 4.89) | 4.80 (4.67, 4.93) | 0.66 |
| Arithmetic mean, model 4 | 4.50 (4.46, 4.54) | 4.41 (4.34, 4.48) | 0.03 |  | 4.19 (4.13, 4.24) | 4.08 (4.00, 4.16) | 0.03 |  | 4.84 (4.78, 4.89) | 4.79 (4.66, 4.92) | 0.55 |
| **ApoB to ApoA1 ratio^4^** |  |  |  |  |  |  |  |  |  |  |  |
| Participants, n | 2,555 | 940 |  |  | 1,173 | 628 |  |  | 1,382 | 312 |  |
| Arithmetic mean, model 1 | 0.75 (0.75, 0.76) | 0.75 (0.74, 0.76) | 0.57 |  | 0.71 (0.70, 0.72) | 0.69 (0.67, 0.70) | 0.04 |  | 0.81 (0.80, 0.82) | 0.82 (0.80, 0.85) | 0.007 |
| Arithmetic mean, model 2 | 0.75 (0.75, 0.76) | 0.75 (0.74, 0.76) | 0.69 |  | 0.71 (0.70, 0.72) | 0.69 (0.67, 0.70) | 0.04 |  | 0.81 (0.80, 0.82) | 0.83 (0.80, 0.85) | 0.003 |
| Arithmetic mean, model 3 | 0.76 (0.75, 0.77) | 0.73 (0.72, 0.75) | 0.001 |  | 0.71 (0.70, 0.72) | 0.68 (0.66, 0.69) | 0.001 |  | 0.81 (0.80, 0.82) | 0.80 (0.77, 0.82) | 0.60 |
| Arithmetic mean, model 4 | 0.76 (0.75, 0.77) | 0.73 (0.72, 0.75) | 0.0003 |  | 0.71 (0.70, 0.72) | 0.68 (0.66, 0.69) | 0.001 |  | 0.81 (0.80, 0.82) | 0.80 (0.77, 0.82) | 0.50 |

1. Unless otherwise specified, all estimates represent adjusted geometric means and 95% CIs, or if stated, adjusted arithmetic means and 95% CIs. Model 1 was adjusted for sex (for analyses with all participants), age (5 years categories) and fasting status (0-1, 2, 3, 4, 5, 6-7, ≥8 hours); model 2 was additionally adjusted for BMI (<20, 20.0-22.4, 22.5-24.9, 25.0-27.4, 27.5-29.9, 30.0-32.4, 32.5-34.9, ≥35.0 kg/m^2^, unknown), model 3 was additionally adjusted for alcohol consumption (<1, 1-7, 8-15, ≥16 g/d, unknown) and smoking status (never, previous, current <15 cigarettes/day, current ≥15 cigarettes/day, unknown), model 4 was additionally adjusted for physical activity (low <10 excess MET hours/week, moderate 10-49 excess MET hours/week, high ≥50 excess MET hours/week, unknown). Arithmetic means were based on model 3 unless otherwise specified.
2. Represents p for heterogeneity across the two diet groups based on Wald tests.
3. Expressed as mmol/L of total cholesterol to mmol/L of HDL-C.
4. Expressed as g/L of ApoB to g/L of ApoA1.

**Supplemental Table 8**: **Bone and joint-related** serum biomarker concentrations (numbers, adjusted means and 95% CIs)^1^ in **white British participants** by diet group in the UK Biobank.

| **Bone and joint-related serum biomarker** | **Regular meat eaters (>3 times/week)^2^** | **Low meat eaters (>3 times/week)^2^** | **Poultry eaters** | **Fish eaters** | **Vegetarians** | **Vegans** | **p-het^3^** |
| --- | --- | --- | --- | --- | --- | --- | --- |
| **Vitamin D, nmol/L** |  |  |  |  |  |  |  |
| Participants, n | 199,723 | 199,842 | 4,600 | 9,534 | 6,183 | 381 |  |
| Model 1 | 44.1 (44.1, 44.2) | 45.9 (45.9, 46.0) | 46.9 (46.4, 47.5) | 45.2 (44.8, 45.6) | 38.1 (37.8, 38.6) | 34.8 (33.4, 36.3) | <0.0001 |
| Model 2 | 44.5 (44.4, 44.6) | 45.7 (45.6, 45.8) | 45.9 (45.4, 46.5) | 44.1 (43.7, 44.4) | 37.3 (37.0, 37.7) | 33.8 (32.4, 35.2) | <0.0001 |
| Model 3 | 44.5 (44.4, 44.5)^a^ | 45.7 (45.6, 45.8)^b^ | 46.3 (45.8, 46.9)^b^ | 44.0 (43.6, 44.3)^a^ | 37.5 (37.1, 37.9)^c^ | 34.4 (33.1, 35.9)^d^ | <0.0001 |
| Model 4 | 44.5 (44.4, 44.6) | 45.7 (45.6, 45.8) | 46.0 (45.5, 46.6) | 43.8 (43.4, 44.1) | 37.4 (37.0, 37.8) | 34.2 (32.8, 35.6) | <0.0001 |
| Model 5 | 44.1 (44.1, 44.2) | 45.3 (45.2, 45.4) | 45.5 (45.0, 46.1) | 43.3 (42.9, 43.6) | 36.9 (36.5, 37.3) | 33.0 (31.6, 34.4) | <0.0001 |
| Arithmetic mean | 49.0 (49.0, 49.1) | 50.3 (50.2, 50.4) | 51.0 (50.5, 51.6) | 48.4 (48.0, 48.8) | 42.5 (42.1, 43.0) | 39.7 (37.8, 41.6) | <0.0001 |
| **Alkaline phosphatase, U/L** |  |  |  |  |  |  |  |
| Participants, n | 208,734 | 209,304 | 4,766 | 9,917 | 6,448 | 399 |  |
| Model 1 | 81.1 (81.0, 81.2) | 79.6 (79.5, 79.7) | 78.5 (77.9, 79.1) | 77.4 (77.0, 77.8) | 80.6 (80.1, 81.1) | 84.0 (81.8, 86.3) | <0.0001 |
| Model 2 | 80.7 (80.6, 80.8) | 79.8 (79.7, 79.9) | 79.9 (79.3, 80.5) | 78.9 (78.5, 79.4) | 82.1 (81.5, 82.6) | 86.4 (84.1, 88.7) | <0.0001 |
| Model 3 | 80.8 (80.7, 80.9)^a^ | 79.8 (79.7, 79.8)^b^ | 79.3 (78.7, 79.9)^bc^ | 78.9 (78.5, 79.4)^c^ | 81.6 (81.1, 82.2)^d^ | 84.9 (82.7, 87.1)^d^ | <0.0001 |
| Model 4 | 80.8 (80.7, 80.9) | 79.8 (79.7, 79.9) | 79.4 (78.8, 80.0) | 79.0 (78.6, 79.5) | 81.7 (81.2, 82.2) | 85.0 (82.8, 87.3) | <0.0001 |
| Arithmetic mean | 84.1 (84.0, 84.2) | 83.0 (82.9, 83.1) | 82.3 (81.6, 83.1) | 82.1 (81.6, 82.6) | 84.8 (84.2, 85.5) | 87.6 (85.1, 90.1) | <0.0001 |
| **Calcium, mmol/L** |  |  |  |  |  |  |  |
| Participants, n | 191,410 | 191,407 | 4,319 | 9,008 | 5,854 | 369 |  |
| Model 1 | 2.38 (2.38, 2.38) | 2.38 (2.38, 2.38) | 2.37 (2.37, 2.38) | 2.37 (2.37, 2.38) | 2.37 (2.37, 2.37) | 2.35 (2.34, 2.36) | <0.0001 |
| Model 2 | 2.38 (2.38, 2.38) | 2.38 (2.38, 2.38) | 2.37 (2.37, 2.38) | 2.37 (2.37, 2.38) | 2.37 (2.37, 2.37) | 2.35 (2.34, 2.36) | <0.0001 |
| Model 3 | 2.38 (2.38, 2.38)^a^ | 2.38 (2.38, 2.38)^a^ | 2.37 (2.37, 2.38)^b^ | 2.37 (2.37, 2.38)^b^ | 2.37 (2.37, 2.37)^c^ | 2.35 (2.34, 2.36)^d^ | <0.0001 |
| Model 4 | 2.38 (2.38, 2.38) | 2.38 (2.38, 2.38) | 2.37 (2.37, 2.38) | 2.37 (2.37, 2.38) | 2.37 (2.37, 2.37) | 2.35 (2.34, 2.36) | <0.0001 |
| Arithmetic mean | 2.38 (2.38, 2.38) | 2.38 (2.38, 2.38) | 2.38 (2.37, 2.38) | 2.38 (2.37, 2.38) | 2.37 (2.37, 2.37) | 2.35 (2.34, 2.36) | <0.0001 |
| Albumin corrected^4^ | 2.27 (2.27, 2.28) | 2.27 (2.27, 2.27) | 2.27 (2.27, 2.27) | 2.27 (2.27, 2.27) | 2.27 (2.27, 2.27) | 2.25 (2.24, 2.26) | <0.0001 |

1. Unless otherwise specified, all estimates represent adjusted geometric means and 95% CIs, or if stated, adjusted arithmetic means and 95% CIs. Model 1 was adjusted for sex, age (5 years categories) and fasting status (0-1, 2, 3, 4, 5, 6-7, ≥8 hours), and for vitamin D month of recruitment; model 2 was additionally adjusted for BMI (<20, 20.0-22.4, 22.5-24.9, 25.0-27.4, 27.5-29.9, 30.0-32.4, 32.5-34.9, ≥35.0 kg/m^2^, unknown), model 3 was additionally adjusted for alcohol consumption (<1, 1-7, 8-15, ≥16 g/d, unknown) and smoking status (never, previous, current <15 cigarettes/day, current ≥15 cigarettes/day, unknown), model 4 was additionally adjusted for physical activity (low <10 excess MET hours/week, moderate 10-49 excess MET hours/week, high ≥50 excess MET hours/week, unknown). For vitamin D, model 5 was model 4 with further exclusion of participants who reported vitamin D supplement use (excluded 6,368 regular meat eaters, 8,593 low meat eaters, 343 poultry eaters, 531 fish eaters, 333 vegetarians, 33 vegans). Arithmetic means and albumin corrected values were based on model 3 unless otherwise specified.
2. Includes participants who consume any red or processed meat, regardless of whether they consume poultry, fish, or dairy. Cut-offs of regular and low consumption determined based on consumption of red and processed meat (beef, lamb, pork, processed meat) as reported on the touchscreen questionnaire.
3. Represents p for heterogeneity across the six diet groups based on Wald tests. Groups that do not share a superscript letter (ordered alphabetically from left to right) were significantly different at the 5% level from post hoc Bonferroni-corrected pairwise comparisons after linear regression (model 3).
4. Albumin-corrected calcium were estimated using the formula serum calcium (mg/dL) + 0.8*(4-albumin [g/dl]). Conversion factors used were 1 mmol/L=4.01 mg/dL for calcium and 10 g/L = 1g/dL for albumin.

**Supplemental Table 9:** **Bone and joint-related** serum biomarker concentrations (numbers, adjusted means and 95% CIs)^1^ in **white British women** by diet group in the UK Biobank.

| **Bone and joint-related serum biomarker** | **Regular meat eaters (>3 times/week)^2^** | **Low meat eaters (>3 times/week)^2^** | **Poultry eaters** | **Fish eaters** | **Vegetarians** | **Vegans** | **p-het^3^** |
| --- | --- | --- | --- | --- | --- | --- | --- |
| **Vitamin D, nmol/L** |  |  |  |  |  |  |  |
| Participants, n | 84,467 | 125,874 | 3,558 | 6,843 | 4,154 | 218 |  |
| Model 1 | 44.3 (44.1, 44.4) | 45.6 (45.5, 45.7) | 46.8 (46.1, 47.5) | 44.9 (44.5, 45.4) | 37.6 (37.2, 38.1) | 33.4 (31.6, 35.4) | <0.0001 |
| Model 2 | 44.8 (44.6, 44.9) | 45.4 (45.3, 45.5) | 45.7 (45.0, 46.3) | 43.6 (43.2, 44.1) | 36.8 (36.3, 37.3) | 32.3 (30.6, 34.1) | <0.0001 |
| Model 3 | 44.7 (44.6, 44.8)^a^ | 45.4 (45.3, 45.5)^b^ | 46.1 (45.5, 46.7)^b^ | 43.6 (43.2, 44.0)^c^ | 37.0 (36.6, 37.5)^d^ | 32.9 (31.2, 34.8)^e^ | <0.0001 |
| Model 4 | 44.8 (44.6, 44.9) | 45.4 (45.3, 45.5) | 45.8 (45.2, 46.5) | 43.5 (43.0, 43.9) | 36.9 (36.4, 37.4) | 32.7 (31.0, 34.6) | <0.0001 |
| Model 5 | 44.2 (44.1, 44.4) | 44.9 (44.8, 45.0) | 45.2 (44.5, 45.8) | 42.8 (42.4, 43.2) | 36.3 (35.8, 36.7) | 31.1 (29.4, 32.9) | <0.0001 |
| Arithmetic mean | 49.3 (49.1, 49.4) | 50.0 (49.9, 50.1) | 50.8 (50.2, 51.4) | 48.1 (47.6, 48.5) | 41.9 (41.3, 42.5) | 38.4 (35.9, 40.9) | <0.0001 |
| **Alkaline phosphatase, U/L** |  |  |  |  |  |  |  |
| Participants, n | 89,785 | 133,348 | 3,704 | 7,156 | 4,350 | 230 |  |
| Model 1 | 82.2 (82.1, 82.4) | 80.7 (80.6, 80.8) | 78.8 (78.1, 79.5) | 78.4 (77.9, 78.9) | 81.8 (81.1, 82.5) | 84.2 (81.3, 87.3) | <0.0001 |
| Model 2 | 81.6 (81.4, 81.7) | 80.9 (80.8, 81.1) | 80.6 (79.9, 81.3) | 80.3 (79.8, 80.8) | 83.5 (82.8, 84.2) | 86.9 (84.0, 90.0) | <0.0001 |
| Model 3 | 81.7 (81.6, 81.8)^a^ | 80.9 (80.8, 81.0)^b^ | 80.0 (79.4, 80.7)^b^ | 80.3 (79.8, 80.8)^b^ | 83.1 (82.4, 83.7)^c^ | 85.6 (82.7, 88.6)^ac^ | <0.0001 |
| Model 4 | 81.7 (81.5, 81.8) | 80.9 (80.8, 81.0) | 80.1 (79.4, 80.8) | 80.3 (79.8, 80.8) | 83.1 (82.5, 83.8) | 85.7 (82.8, 88.7) | <0.0001 |
| Arithmetic mean | 85.4 (85.2, 85.6) | 84.5 (84.3, 84.6) | 83.5 (82.6, 84.3) | 83.8 (83.2, 84.4) | 86.4 (85.7, 87.2) | 88.8 (85.5, 92.1) | <0.0001 |
| **Calcium, mmol/L** |  |  |  |  |  |  |  |
| Participants, n | 81,507 | 121,318 | 3,346 | 6,461 | 3,921 | 216 |  |
| Model 1 | 2.39 (2.38, 2.39) | 2.39 (2.38, 2.39) | 2.38 (2.38, 2.38) | 2.38 (2.38, 2.38) | 2.38 (2.37, 2.38) | 2.36 (2.34, 2.37) | <0.0001 |
| Model 2 | 2.39 (2.39, 2.39) | 2.39 (2.38, 2.39) | 2.38 (2.38, 2.38) | 2.38 (2.38, 2.38) | 2.38 (2.37, 2.38) | 2.35 (2.34, 2.37) | <0.0001 |
| Model 3 | 2.39 (2.39, 2.39)^a^ | 2.39 (2.38, 2.39)^ab^ | 2.38 (2.38, 2.38)^bc^ | 2.38 (2.38, 2.38)^c^ | 2.38 (2.37, 2.38)^c^ | 2.36 (2.34, 2.37)^d^ | <0.0001 |
| Model 4 | 2.39 (2.38, 2.39) | 2.39 (2.38, 2.39) | 2.38 (2.38, 2.38) | 2.38 (2.38, 2.38) | 2.38 (2.37, 2.38) | 2.36 (2.34, 2.37) | <0.0001 |
| Arithmetic mean | 2.39 (2.39, 2.39) | 2.39 (2.39, 2.39) | 2.38 (2.38, 2.39) | 2.38 (2.38, 2.39) | 2.38 (2.37, 2.38) | 2.36 (2.34, 2.37) | <0.0001 |
| Albumin corrected^4^ | 2.29 (2.29, 2.29) | 2.29 (2.29, 2.29) | 2.28 (2.28, 2.29) | 2.28 (2.28, 2.28) | 2.28 (2.28, 2.28) | 2.26 (2.25, 2.27) | <0.0001 |

1. Unless otherwise specified, all estimates represent adjusted geometric means and 95% CIs, or if stated, adjusted arithmetic means and 95% CIs. Model 1 was adjusted for age (5 years categories) and fasting status (0-1, 2, 3, 4, 5, 6-7, ≥8 hours), and for vitamin D month of recruitment; model 2 was additionally adjusted for BMI (<20, 20.0-22.4, 22.5-24.9, 25.0-27.4, 27.5-29.9, 30.0-32.4, 32.5-34.9, ≥35.0 kg/m^2^, unknown), model 3 was additionally adjusted for alcohol consumption (<1, 1-7, 8-15, ≥16 g/d, unknown) and smoking status (never, previous, current <15 cigarettes/day, current ≥15 cigarettes/day, unknown), model 4 was additionally adjusted for physical activity (low <10 excess MET hours/week, moderate 10-49 excess MET hours/week, high ≥50 excess MET hours/week, unknown). For vitamin D, model 5 was model 4 with further exclusion of participants who reported vitamin D supplement use (excluded 3,781 regular meat eaters, 6,581 low meat eaters, 278 poultry eaters, 424 fish eaters, 257 vegetarians, 19 vegans). Arithmetic means and albumin corrected values were based on model 3 unless otherwise specified.
2. Includes participants who consume any red or processed meat, regardless of whether they consume poultry, fish, or dairy. Cut-offs of regular and low consumption determined based on consumption of red and processed meat (beef, lamb, pork, processed meat) as reported on the touchscreen questionnaire.
3. Represents p for heterogeneity across the six diet groups based on Wald tests. ﻿ Groups that do not share a superscript letter (ordered alphabetically from left to right) were significantly different at the 5% level from post hoc Bonferroni-corrected pairwise comparisons after linear regression (model 3).
4. Albumin-corrected calcium were estimated using the formula serum calcium (mg/dL) + 0.8*(4-albumin [g/dl]). Conversion factors used were 1 mmol/L=4.01 mg/dL for calcium and 10 g/L = 1g/dL for albumin.

**Supplemental Table 10:** **Bone and joint-related** serum biomarker concentrations (numbers, adjusted means and 95% CIs)^1^ in **white British men** by diet group in the UK Biobank.

| **Bone and joint-related serum biomarker** | **Regular meat eaters (>3 times/week)^2^** | **Low meat eaters (>3 times/week)^2^** | **Poultry eaters** | **Fish eaters** | **Vegetarians** | **Vegans** | **p-het^3^** |
| --- | --- | --- | --- | --- | --- | --- | --- |
| **Vitamin D, nmol/L** |  |  |  |  |  |  |  |
| Participants, n | 115,256 | 73,968 | 1,042 | 2,691 | 2,029 | 163 |  |
| Model 1 | 44.1 (44.0, 44.2) | 46.4 (46.3, 46.5) | 47.1 (46.0, 48.3) | 45.6 (44.9, 46.3) | 39.0 (38.3, 39.7) | 36.7 (34.5, 39.1) | <0.0001 |
| Model 2 | 44.3 (44.2, 44.4) | 46.2 (46.0, 46.3) | 46.3 (45.2, 47.5) | 44.7 (44.1, 45.4) | 38.4 (37.7, 39.1) | 36.1 (34.0, 38.4) | <0.0001 |
| Model 3 | 44.3 (44.2, 44.4)^a^ | 46.1 (46.0, 46.2)^b^ | 46.7 (45.6, 47.8)^b^ | 44.6 (44.0, 45.3)^a^ | 38.5 (37.8, 39.1)^c^ | 36.7 (34.5, 39.0)^c^ | <0.0001 |
| Model 4 | 44.4 (44.3, 44.5) | 46.1 (46.0, 46.2) | 46.2 (45.1, 47.3) | 44.4 (43.7, 45.0) | 38.4 (37.7, 39.0) | 36.4 (34.3, 38.7) | <0.0001 |
| Model 5 | 44.2 (44.1, 44.3) | 45.9 (45.7, 46.0) | 46.0 (44.9, 47.1) | 44.0 (43.3, 44.7) | 38.0 (37.3, 38.7) | 35.8 (33.6, 38.1) | <0.0001 |
| Arithmetic mean | 48.9 (48.8, 49.1) | 50.7 (50.5, 50.8) | 51.3 (50.2, 52.4) | 49.0 (48.3, 49.7) | 43.7 (42.9, 44.5) | 41.5 (38.7, 44.4) | <0.0001 |
| **Alkaline phosphatase, U/L** |  |  |  |  |  |  |  |
| Participants, n | 118,949 | 75,956 | 1,062 | 2,761 | 2,098 | 169 |  |
| Model 1 | 79.7 (79.6, 79.8) | 78.3 (78.2, 78.4) | 79.7 (78.4, 80.9) | 77.6 (76.9, 78.4) | 80.2 (79.3, 81.1) | 84.1 (80.9, 87.5) | <0.0001 |
| Model 2 | 79.6 (79.5, 79.7) | 78.4 (78.3, 78.6) | 80.2 (79.0, 81.5) | 78.2 (77.4, 78.9) | 80.8 (79.9, 81.7) | 84.8 (81.5, 88.2) | <0.0001 |
| Model 3 | 79.6 (79.5, 79.7)^a^ | 78.4 (78.3, 78.6)^b^ | 79.6 (78.4, 80.8)^ab^ | 78.1 (77.4, 78.9)^b^ | 80.4 (79.6, 81.3)^a^ | 83.4 (80.2, 86.7)^a^ | <0.0001 |
| Model 4 | 79.6 (79.4, 79.7) | 78.4 (78.3, 78.6) | 79.7 (78.4, 80.9) | 78.2 (77.5, 79.0) | 80.5 (79.6, 81.4) | 83.6 (80.4, 87.0) | <0.0001 |
| Arithmetic mean | 82.5 (82.4, 82.6) | 81.3 (81.1, 81.4) | 82.2 (80.7, 83.6) | 80.8 (79.9, 81.8) | 83.5 (82.4, 84.6) | 85.6 (81.9, 89.4) | <0.0001 |
| **Calcium, mmol/L** |  |  |  |  |  |  |  |
| Participants, n | 109,903 | 70,089 | 973 | 2,547 | 1,933 | 153 |  |
| Model 1 | 2.37 (2.37, 2.37) | 2.37 (2.37, 2.37) | 2.36 (2.36, 2.37) | 2.37 (2.36, 2.37) | 2.36 (2.36, 2.37) | 2.34 (2.33, 2.36) | <0.0001 |
| Model 2 | 2.37 (2.37, 2.37) | 2.37 (2.37, 2.37) | 2.36 (2.36, 2.37) | 2.37 (2.36, 2.37) | 2.36 (2.36, 2.37) | 2.34 (2.33, 2.36) | <0.0001 |
| Model 3 | 2.37 (2.37, 2.37)^a^ | 2.37 (2.37, 2.37)^a^ | 2.37 (2.36, 2.37)^abc^ | 2.37 (2.36, 2.37)^ab^ | 2.36 (2.36, 2.37)^bc^ | 2.35 (2.33, 2.36)^c^ | <0.0001 |
| Model 4 | 2.37 (2.37, 2.37) | 2.37 (2.37, 2.37) | 2.37 (2.36, 2.37) | 2.37 (2.36, 2.37) | 2.36 (2.36, 2.37) | 2.35 (2.33, 2.36) | <0.0001 |
| Arithmetic mean | 2.37 (2.37, 2.37) | 2.37 (2.37, 2.37) | 2.37 (2.36, 2.37) | 2.37 (2.37, 2.37) | 2.36 (2.36, 2.37) | 2.35 (2.33, 2.36) | <0.0001 |
| Albumin corrected^4^ | 2.26 (2.26, 2.26) | 2.26 (2.26, 2.26) | 2.25 (2.25, 2.26) | 2.26 (2.25, 2.26) | 2.26 (2.25, 2.26) | 2.24 (2.23, 2.25) | <0.0001 |

1. Unless otherwise specified, all estimates represent adjusted geometric means and 95% CIs, or if stated, adjusted arithmetic means and 95% CIs. Model 1 was adjusted for age (5 years categories) and fasting status (0-1, 2, 3, 4, 5, 6-7, ≥8 hours), and for vitamin D month of recruitment; model 2 was additionally adjusted for BMI (<20, 20.0-22.4, 22.5-24.9, 25.0-27.4, 27.5-29.9, 30.0-32.4, 32.5-34.9, ≥35.0 kg/m^2^, unknown), model 3 was additionally adjusted for alcohol consumption (<1, 1-7, 8-15, ≥16 g/d, unknown) and smoking status (never, previous, current <15 cigarettes/day, current ≥15 cigarettes/day, unknown), model 4 was additionally adjusted for physical activity (low <10 excess MET hours/week, moderate 10-49 excess MET hours/week, high ≥50 excess MET hours/week, unknown). For vitamin D, model 5 was model 4 with further exclusion of participants who reported vitamin D supplement use (excluded 2,587 regular meat eaters, 2,012 low meat eaters, 65 poultry eaters, 107 fish eaters, 76 vegetarians, 14 vegans). Arithmetic means and albumin corrected values were based on model 3 unless otherwise specified.
2. Includes participants who consume any red or processed meat, regardless of whether they consume poultry, fish, or dairy. Cut-offs of regular and low consumption determined based on consumption of red and processed meat (beef, lamb, pork, processed meat) as reported on the touchscreen questionnaire.
3. Represents p for heterogeneity across the six diet groups based on Wald tests. Groups that do not share a superscript letter (ordered alphabetically from left to right) were significantly different at the 5% level from post hoc Bonferroni-corrected pairwise comparisons after linear regression (model 3).
4. Albumin-corrected calcium were estimated using the formula serum calcium (mg/dL) + 0.8*(4-albumin [g/dl]). Conversion factors used were 1 mmol/L=4.01 mg/dL for calcium and 10 g/L = 1g/dL for albumin.

**Supplemental Table 11**: **Bone and joint-related** serum biomarker concentrations (numbers, adjusted means and 95% CIs)^1^ in **British Indian participants** by diet group in the UK Biobank.

| **Bone and joint-related serum biomarker** | **All participants** | | |  | **Women** | | |  | **Men** | | |
| --- | --- | --- | --- | --- | --- | --- | --- | --- | --- | --- | --- |
|  | **Meat-eaters** | **Vegetarians** | **p-het^2^** |  | **Meat-eaters** | **Vegetarians** | **p-het^2^** |  | **Meat-eaters** | **Vegetarians** | **p-het^2^** |
| **Vitamin D, nmol/L** |  |  |  |  |  |  |  |  |  |  |  |
| Participants, n | 3,431 | 1,056 |  |  | 1,441 | 687 |  |  | 1,990 | 369 |  |
| Model 1 | 24.8 (24.4, 25.3) | 22.5 (21.8, 23.1) | <0.0001 |  | 26.1 (25.4, 26.8) | 24.7 (23.7, 25.7) | 0.02 |  | 23.7 (23.2, 24.2) | 20.0 (19.1, 21.0) | <0.0001 |
| Model 2 | 24.9 (24.5, 25.3) | 22.4 (21.7, 23.1) | <0.0001 |  | 26.1 (25.4, 26.8) | 24.7 (23.7, 25.7) | 0.02 |  | 23.7 (23.3, 24.2) | 19.9 (19.0, 20.8) | <0.0001 |
| Model 3 | 24.7 (24.3, 25.1) | 22.9 (22.2, 23.7) | 0.0001 |  | 25.8 (25.1, 26.5) | 25.3 (24.3, 26.3) | 0.40 |  | 23.6 (23.2, 24.1) | 20.3 (19.3, 21.3) | <0.0001 |
| Model 4 | 24.7 (24.3, 25.1) | 23.0 (22.3, 23.7) | 0.0001 |  | 25.8 (25.1, 26.5) | 25.3 (24.3, 26.3) | 0.43 |  | 23.6 (23.2, 24.1) | 20.3 (19.3, 21.3) | <0.0001 |
| Model 5 | 24.0 (23.6, 24.4) | 21.9 (21.2, 22.6) | <0.0001 |  | 24.8 (24.1, 25.5) | 23.6 (22.6, 24.6) | 0.06 |  | 23.3 (22.8, 23.8) | 19.9 (18.9, 21.0) | <0.0001 |
| Arithmetic mean | 27.9 (27.4, 28.5) | 27.2 (26.2, 28.2) | 0.19 |  | 29.4 (28.4, 30.3) | 30.2 (28.8, 31.6) | 0.37 |  | 26.5 (26.0, 27.1) | 23.3 (21.9, 24.8) | <0.0001 |
| **Alkaline phosphatase, U/L** |  |  |  |  |  |  |  |  |  |  |  |
| Participants, n | 3,821 | 1,342 |  |  | 1,604 | 869 |  |  | 2,217 | 473 |  |
| Model 1 | 84.1 (83.4, 84.8) | 88.9 (87.6, 90.1) | <0.0001 |  | 85.7 (84.6, 86.8) | 90.7 (89.1, 92.3) | <0.0001 |  | 82.7 (81.8, 83.6) | 86.7 (84.7, 88.7) | 0.0004 |
| Model 2 | 84.0 (83.3, 84.7) | 89.0 (87.7, 90.3) | <0.0001 |  | 85.7 (84.6, 86.8) | 90.7 (89.2, 92.3) | <0.0001 |  | 82.7 (81.8, 83.6) | 86.7 (84.7, 88.7) | 0.0003 |
| Model 3 | 84.4 (83.7, 85.1) | 87.9 (86.7, 89.3) | <0.0001 |  | 86.0 (84.9, 87.1) | 90.1 (88.5, 91.7) | <0.0001 |  | 83.0 (82.1, 83.9) | 85.3 (83.2, 87.5) | 0.05 |
| Model 4 | 84.4 (83.7, 85.1) | 87.9 (86.6, 89.2) | <0.0001 |  | 86.0 (84.9, 87.1) | 90.1 (88.5, 91.7) | <0.0001 |  | 83.0 (82.1, 83.9) | 85.3 (83.2, 87.4) | 0.06 |
| Arithmetic mean | 87.5 (86.7, 88.3) | 91.3 (89.9, 92.7) | <0.0001 |  | 89.4 (88.2, 90.6) | 93.6 (91.9, 95.3) | 0.0001 |  | 85.8 (84.8, 86.8) | 88.4 (86.1, 90.6) | 0.05 |
| **Calcium, mmol/L** |  |  |  |  |  |  |  |  |  |  |  |
| Participants, n | 3,495 | 1,205 |  |  | 1,449 | 776 |  |  | 2,046 | 429 |  |
| Model 1 | 2.37 (2.37, 2.38) | 2.37 (2.36, 2.37) | 0.14 |  | 2.38 (2.38, 2.39) | 2.37 (2.37, 2.38) | 0.13 |  | 2.37 (2.36, 2.37) | 2.36 (2.35, 2.37) | 0.36 |
| Model 2 | 2.37 (2.37, 2.38) | 2.37 (2.36, 2.37) | 0.14 |  | 2.38 (2.38, 2.39) | 2.37 (2.37, 2.38) | 0.14 |  | 2.37 (2.36, 2.37) | 2.36 (2.36, 2.37) | 0.42 |
| Model 3 | 2.37 (2.37, 2.38) | 2.37 (2.36, 2.38) | 0.29 |  | 2.38 (2.38, 2.39) | 2.37 (2.37, 2.38) | 0.24 |  | 2.37 (2.36, 2.37) | 2.37 (2.36, 2.38) | 0.85 |
| Model 4 | 2.37 (2.37, 2.38) | 2.37 (2.36, 2.38) | 0.27 |  | 2.38 (2.38, 2.39) | 2.37 (2.37, 2.38) | 0.23 |  | 2.37 (2.36, 2.37) | 2.37 (2.36, 2.38) | 0.85 |
| Arithmetic mean | 2.38 (2.37, 2.38) | 2.37 (2.37, 2.38) | 0.30 |  | 2.38 (2.38, 2.39) | 2.38 (2.37, 2.38) | 0.27 |  | 2.37 (2.37, 2.37) | 2.37 (2.36, 2.38) | 0.83 |
| Albumin corrected^3^ | 2.28 (2.27, 2.28) | 2.28 (2.27, 2.28) | 0.49 |  | 2.30 (2.29, 2.30) | 2.30 (2.29, 2.30) | 0.69 |  | 2.26 (2.26, 2.27) | 2.26 (2.26, 2.27) | 0.70 |

1. Unless otherwise specified, all estimates represent adjusted geometric means and 95% CIs, or if stated, adjusted arithmetic means and 95% CIs. Model 1 was adjusted for sex (for analyses with all participants), age (5 years categories) and fasting status (0-1, 2, 3, 4, 5, 6-7, ≥8 hours), and for vitamin D month of recruitment;; model 2 was additionally adjusted for BMI (<20, 20.0-22.4, 22.5-24.9, 25.0-27.4, 27.5-29.9, 30.0-32.4, 32.5-34.9, ≥35.0 kg/m^2^, unknown), model 3 was additionally adjusted for alcohol consumption (<1, 1-7, 8-15, ≥16 g/d, unknown) and smoking status (never, previous, current <15 cigarettes/day, current ≥15 cigarettes/day, unknown), model 4 was additionally adjusted for physical activity (low <10 excess MET hours/week, moderate 10-49 excess MET hours/week, high ≥50 excess MET hours/week, unknown). For vitamin D, model 5 was model 4 with further exclusion of participants who reported vitamin D supplement use (excluded 141 meat eaters and 108 vegetarians in women, and 94 meat eaters and 14 vegetarians in men). Arithmetic means and albumin corrected values were based on model 3 unless otherwise specified.
2. Represents p for heterogeneity across the two diet groups based on Wald tests.
3. Albumin-corrected calcium were estimated using the formula serum calcium (mg/dL) + 0.8*(4-albumin [g/dl]). Conversion factors used were 1 mmol/L=4.01 mg/dL for calcium and 10 g/L = 1g/dL for albumin.

**Supplemental Table 12**: **Cancer-related** serum biomarker concentrations (numbers, adjusted means and 95% CIs)^1^ in **white British participants** by diet group in the UK Biobank.

| **Cancer-related serum biomarker** | **Regular meat eaters (>3 times/week)^2^** | **Low meat eaters (>3 times/week)^2^** | **Poultry eaters** | **Fish eaters** | **Vegetarians** | **Vegans** | **p-het^3^** |
| --- | --- | --- | --- | --- | --- | --- | --- |
| **Sex hormone-binding globulin, nmol/L** |  |  |  |  |  |  |  |
| Participants, n | 189,633 | 189,537 | 4,283 | 8,922 | 5,795 | 362 |  |
| Model 1 | 44.2 (44.1, 44.3) | 46.8 (46.7, 46.9) | 53.4 (52.6, 54.1) | 53.7 (53.2, 54.2) | 50.2 (49.6, 50.8) | 57.3 (54.7, 60.1) | <0.0001 |
| Model 2 | 45.0 (44.9, 45.1) | 46.3 (46.2, 46.4) | 49.2 (48.6, 49.9) | 49.3 (48.8, 49.7) | 46.3 (45.8, 46.8) | 50.5 (48.3, 52.7) | <0.0001 |
| Model 3 | 45.0 (45.0, 45.1)^a^ | 46.3 (46.2, 46.4)^b^ | 49.1 (48.5, 49.7)^c^ | 49.3 (48.9, 49.7)^c^ | 46.2 (45.7, 46.8)^b^ | 50.2 (48.1, 52.4)^c^ | <0.0001 |
| Model 4 | 45.1 (45.0, 45.1) | 46.3 (46.2, 46.4) | 49.0 (48.4, 49.6) | 49.2 (48.8, 49.7) | 46.2 (45.7, 46.7) | 50.1 (48.0, 52.3) | <0.0001 |
| Arithmetic mean | 51.2 (51.1, 51.3) | 52.5 (52.3, 52.6) | 56.6 (55.9, 57.3) | 56.3 (55.8, 56.8) | 52.4 (51.8, 53.0) | 56.9 (54.5, 59.4) | <0.0001 |
| **Testosterone, nmol/L** |  |  |  |  |  |  |  |
| Participants, n | 206,793 | 207,519 | 4,727 | 9,850 | 6,406 | 393 |  |
| Model 1 | 2.70 (2.70, 2.71) | 2.71 (2.71, 2.72) | 2.62 (2.58, 2.66) | 2.65 (2.62, 2.68) | 2.61 (2.58, 2.64) | 2.73 (2.59, 2.88) | <0.0001 |
| Model 2 | 2.70 (2.70, 2.71) | 2.71 (2.71, 2.72) | 2.63 (2.59, 2.67) | 2.66 (2.63, 2.68) | 2.62 (2.58, 2.65) | 2.75 (2.61, 2.90) | <0.0001 |
| Model 3 | 2.70 (2.69, 2.70)^a^ | 2.72 (2.71, 2.72)^b^ | 2.65 (2.61, 2.69)^ac^ | 2.67 (2.64, 2.70)^ac^ | 2.64 (2.60, 2.67)^c^ | 2.80 (2.66, 2.95)^abc^ | <0.0001 |
| Model 4 | 2.70 (2.69, 2.70) | 2.72 (2.71, 2.72) | 2.65 (2.61, 2.69) | 2.67 (2.64, 2.70) | 2.64 (2.60, 2.67) | 2.80 (2.66, 2.94) | <0.0001 |
| Arithmetic mean | 5.98 (5.97, 5.99) | 6.04 (6.03, 6.05) | 6.02 (5.95, 6.09) | 6.05 (6.00, 6.10) | 5.97 (5.91, 6.03) | 6.25 (6.00, 6.50) | <0.0001 |
| **Insulin-like growth factor I, nmol/L** |  |  |  |  |  |  |  |
| Participants, n | 207,591 | 208,182 | 4,737 | 9,865 | 6,413 | 396 |  |
| Model 1 | 20.5 (20.5, 20.5) | 20.8 (20.8, 20.8) | 20.9 (20.8, 21.1) | 20.7 (20.5, 20.8) | 19.9 (19.7, 20.0) | 19.3 (18.8, 19.8) | <0.0001 |
| Model 2 | 20.5 (20.5, 20.6) | 20.7 (20.7, 20.8) | 20.8 (20.6, 20.9) | 20.5 (20.4, 20.6) | 19.7 (19.6, 19.9) | 19.2 (18.7, 19.7) | <0.0001 |
| Model 3 | 20.6 (20.6, 20.6)^ab^ | 20.7 (20.7, 20.7)^c^ | 20.8 (20.6, 21.0)^bc^ | 20.4 (20.3, 20.6)^a^ | 19.7 (19.6, 19.9)^d^ | 19.2 (18.7, 19.7)^d^ | <0.0001 |
| Model 4 | 20.6 (20.6, 20.6) | 20.7 (20.7, 20.7) | 20.8 (20.6, 20.9) | 20.4 (20.3, 20.5) | 19.7 (19.6, 19.8) | 19.2 (18.7, 19.7) | <0.0001 |
| Arithmetic mean | 21.3 (21.3, 21.4) | 21.5 (21.5, 21.5) | 21.6 (21.4, 21.7) | 21.2 (21.1, 21.3) | 20.5 (20.3, 20.6) | 20.0 (19.5, 20.6) | <0.0001 |

1. Unless otherwise specified, all estimates represent adjusted geometric means and 95% CIs, or if stated, adjusted arithmetic means and 95% CIs. Model 1 was adjusted for sex, age (5 years categories) and fasting status (0-1, 2, 3, 4, 5, 6-7, ≥8 hours); model 2 was additionally adjusted for BMI (<20, 20.0-22.4, 22.5-24.9, 25.0-27.4, 27.5-29.9, 30.0-32.4, 32.5-34.9, ≥35.0 kg/m^2^, unknown), model 3 was additionally adjusted for alcohol consumption (<1, 1-7, 8-15, ≥16 g/d, unknown) and smoking status (never, previous, current <15 cigarettes/day, current ≥15 cigarettes/day, unknown), model 4 was additionally adjusted for physical activity (low <10 excess MET hours/week, moderate 10-49 excess MET hours/week, high ≥50 excess MET hours/week, unknown). Arithmetic means were based on model 3 unless otherwise specified.
2. Includes participants who consume any red or processed meat, regardless of whether they consume poultry, fish, or dairy. Cut-offs of regular and low consumption determined based on consumption of red and processed meat (beef, lamb, pork, processed meat) as reported on the touchscreen questionnaire.
3. Represents p for heterogeneity across the six diet groups based on Wald tests. Groups that do not share a superscript letter (ordered alphabetically from left to right) were significantly different at the 5% level from post hoc Bonferroni-corrected pairwise comparisons after linear regression (model 3).

**Supplemental Table 13**: **Cancer-related** serum biomarker concentrations (numbers, adjusted means and 95% CIs)^1^ in **white British women** by diet group in the UK Biobank.

| **Cancer-related serum biomarker** | **Regular meat eaters (>3 times/week)^2^** | **Low meat eaters (>3 times/week)^2^** | **Poultry eaters** | **Fish eaters** | **Vegetarians** | **Vegans** | **p-het^3^** |
| --- | --- | --- | --- | --- | --- | --- | --- |
| **Sex hormone-binding globulin, nmol/L** |  |  |  |  |  |  |  |
| Participants, n | 80,569 | 119,968 | 3,313 | 6,393 | 3,876 | 210 |  |
| Model 1 | 52.6 (52.4, 52.8) | 56.5 (56.4, 56.7) | 64.4 (63.3, 65.5) | 64.8 (64.1, 65.6) | 60.3 (59.4, 61.2) | 69.1 (64.7, 73.9) | <0.0001 |
| Model 2 | 54.1 (54.0, 54.3) | 56.0 (55.8, 56.1) | 59.4 (58.6, 60.3) | 59.5 (58.8, 60.1) | 55.9 (55.1, 56.7) | 61.2 (57.7, 65.0) | <0.0001 |
| Model 3 | 54.2 (54.0, 54.4)^a^ | 55.9 (55.8, 56.1)^b^ | 59.2 (58.3, 60.1)^c^ | 59.4 (58.8, 60.1)^c^ | 55.7 (55.0, 56.5)^b^ | 60.7 (57.2, 64.4)^bc^ | <0.0001 |
| Model 4 | 54.2 (54.1, 54.4) | 55.9 (55.8, 56.1) | 59.1 (58.2, 60.0) | 59.4 (58.7, 60.0) | 55.7 (54.9, 56.4) | 60.6 (57.1, 64.4) | <0.0001 |
| Arithmetic mean | 61.1 (60.9, 61.3) | 62.9 (62.7, 63.0) | 67.0 (66.0, 68.0) | 66.9 (66.2, 67.6) | 62.6 (61.7, 63.5) | 68.5 (64.7, 72.3) | <0.0001 |
| **Testosterone, nmol/L** |  |  |  |  |  |  |  |
| Participants, n | 89,030 | 132,293 | 3,674 | 7,104 | 4,325 | 227 |  |
| Model 1 | 0.82 (0.81, 0.82) | 0.81 (0.80, 0.81) | 0.76 (0.74, 0.78) | 0.76 (0.75, 0.77) | 0.74 (0.73, 0.76) | 0.77 (0.71, 0.83) | <0.0001 |
| Model 2 | 0.81 (0.81, 0.81) | 0.81 (0.80, 0.81) | 0.78 (0.76, 0.80) | 0.78 (0.77, 0.79) | 0.76 (0.75, 0.78) | 0.80 (0.73, 0.87) | <0.0001 |
| Model 3 | 0.81 (0.80, 0.81)^a^ | 0.81 (0.81, 0.81)^a^ | 0.79 (0.77, 0.81)^ab^ | 0.78 (0.77, 0.80)^b^ | 0.77 (0.76, 0.79)^b^ | 0.82 (0.75, 0.89)^ab^ | <0.0001 |
| Model 4 | 0.81 (0.80, 0.81) | 0.81 (0.81, 0.81) | 0.79 (0.77, 0.81) | 0.78 (0.77, 0.80) | 0.77 (0.76, 0.79) | 0.82 (0.75, 0.89) | <0.0001 |
| Arithmetic mean | 0.99 (0.98, 0.99) | 0.98 (0.98, 0.99) | 0.97 (0.95, 0.99) | 0.95 (0.94, 0.97) | 0.94 (0.92, 0.96) | 0.98 (0.90, 1.07) | <0.0001 |
| **Insulin-like growth factor I, nmol/L** |  |  |  |  |  |  |  |
| Participants, n | 89,252 | 132,635 | 3,681 | 7,123 | 4,327 | 228 |  |
| Model 1 | 20.1 (20.0, 20.1) | 20.2 (20.2, 20.3) | 20.4 (20.2, 20.6) | 20.0 (19.9, 20.1) | 19.2 (19.0, 19.3) | 18.4 (17.8, 19.1) | <0.0001 |
| Model 2 | 20.2 (20.1, 20.2) | 20.2 (20.2, 20.2) | 20.2 (20.0, 20.4) | 19.8 (19.7, 19.9) | 19.1 (18.9, 19.2) | 18.3 (17.7, 18.9) | <0.0001 |
| Model 3 | 20.2 (20.1, 20.2)^a^ | 20.2 (20.2, 20.2)^a^ | 20.3 (20.1, 20.4)^a^ | 19.8 (19.7, 20.0)^b^ | 19.1 (18.9, 19.2)^c^ | 18.4 (17.7, 19.0)^c^ | <0.0001 |
| Model 4 | 20.2 (20.1, 20.2) | 20.2 (20.2, 20.2) | 20.2 (20.1, 20.4) | 19.8 (19.7, 19.9) | 19.1 (18.9, 19.2) | 18.3 (17.7, 19.0) | <0.0001 |
| Arithmetic mean | 21.0 (20.9, 21.0) | 21.0 (20.9, 21.0) | 21.0 (20.9, 21.2) | 20.6 (20.5, 20.7) | 19.8 (19.7, 20.0) | 19.3 (18.6, 20.0) | <0.0001 |

1. Unless otherwise specified, all estimates represent adjusted geometric means and 95% CIs, or if stated, adjusted arithmetic means and 95% CIs. Model 1 was adjusted for age (5 years categories) and fasting status (0-1, 2, 3, 4, 5, 6-7, ≥8 hours); model 2 was additionally adjusted for BMI (<20, 20.0-22.4, 22.5-24.9, 25.0-27.4, 27.5-29.9, 30.0-32.4, 32.5-34.9, ≥35.0 kg/m^2^, unknown), model 3 was additionally adjusted for alcohol consumption (<1, 1-7, 8-15, ≥16 g/d, unknown) and smoking status (never, previous, current <15 cigarettes/day, current ≥15 cigarettes/day, unknown), model 4 was additionally adjusted for physical activity (low <10 excess MET hours/week, moderate 10-49 excess MET hours/week, high ≥50 excess MET hours/week, unknown). Arithmetic means were based on model 3 unless otherwise specified.
2. Includes participants who consume any red or processed meat, regardless of whether they consume poultry, fish, or dairy. Cut-offs of regular and low consumption determined based on consumption of red and processed meat (beef, lamb, pork, processed meat) as reported on the touchscreen questionnaire.
3. Represents p for heterogeneity across the six diet groups based on Wald tests. Groups that do not share a superscript letter (ordered alphabetically from left to right) were significantly different at the 5% level from post hoc Bonferroni-corrected pairwise comparisons after linear regression (model 3).

**Supplemental Table 14**: **Cancer-related** serum biomarker concentrations (numbers, adjusted means and 95% CIs)^1^ in **white British men** by diet group in the UK Biobank.

| **Cancer-related serum biomarker** | **Regular meat eaters (>3 times/week)^2^** | **Low meat eaters (>3 times/week)^2^** | **Poultry eaters** | **Fish eaters** | **Vegetarians** | **Vegans** | **p-het^3^** |
| --- | --- | --- | --- | --- | --- | --- | --- |
| **Sex hormone-binding globulin, nmol/L** |  |  |  |  |  |  |  |
| Participants, n | 109,064 | 69,569 | 970 | 2,529 | 1,919 | 152 |  |
| Model 1 | 36.0 (35.9, 36.1) | 37.4 (37.2, 37.5) | 42.4 (41.3, 43.5) | 41.8 (41.1, 42.4) | 39.5 (38.8, 40.2) | 46.2 (43.4, 49.2) | <0.0001 |
| Model 2 | 36.3 (36.2, 36.4) | 37.1 (36.9, 37.2) | 39.5 (38.6, 40.5) | 38.7 (38.1, 39.2) | 36.6 (36.0, 37.2) | 40.9 (38.5, 43.4) | <0.0001 |
| Model 3 | 36.3 (36.2, 36.4)^a^ | 37.1 (37.0, 37.2)^b^ | 39.4 (38.4, 40.3)^c^ | 38.8 (38.2, 39.3)^c^ | 36.5 (35.9, 37.2)^ab^ | 40.6 (38.2, 43.1)^c^ | <0.0001 |
| Model 4 | 36.3 (36.2, 36.4) | 37.1 (37.0, 37.2) | 39.2 (38.3, 40.1) | 38.7 (38.1, 39.3) | 36.5 (35.9, 37.1) | 40.5 (38.1, 43.0) | <0.0001 |
| Arithmetic mean | 39.5 (39.4, 39.6) | 40.3 (40.1, 40.4) | 43.2 (42.2, 44.1) | 42.1 (41.5, 42.7) | 39.9 (39.2, 40.6) | 44.2 (41.8, 46.6) | <0.0001 |
| **Testosterone, nmol/L** |  |  |  |  |  |  |  |
| Participants, n | 117,763 | 75,226 | 1,053 | 2,746 | 2,081 | 166 |  |
| Model 1 | 11.3 (11.2, 11.3) | 11.5 (11.5, 11.6) | 12.1 (11.9, 12.4) | 12.2 (12.1, 12.4) | 11.8 (11.7, 12.0) | 12.5 (11.9, 13.1) | <0.0001 |
| Model 2 | 11.4 (11.3, 11.4) | 11.5 (11.4, 11.5) | 11.6 (11.4, 11.8) | 11.7 (11.5, 11.8) | 11.3 (11.1, 11.4) | 11.6 (11.1, 12.2) | <0.0001 |
| Model 3 | 11.3 (11.3, 11.4)^a^ | 11.5 (11.4, 11.5)^b^ | 11.6 (11.4, 11.8)^abc^ | 11.7 (11.6, 11.8)^c^ | 11.3 (11.2, 11.5)^ab^ | 11.7 (11.2, 12.3)^abc^ | <0.0001 |
| Model 4 | 11.3 (11.3, 11.4) | 11.5 (11.4, 11.5) | 11.6 (11.4, 11.8) | 11.7 (11.6, 11.8) | 11.3 (11.2, 11.5) | 11.7 (11.1, 12.3) | <0.0001 |
| Arithmetic mean | 11.9 (11.9, 11.9) | 12.1 (12.0, 12.1) | 12.3 (12.1, 12.5) | 12.3 (12.2, 12.4) | 11.9 (11.8, 12.1) | 12.3 (11.8, 12.8) | <0.0001 |
| **Insulin-like growth factor I, nmol/L** |  |  |  |  |  |  |  |
| Participants, n | 118,339 | 75,547 | 1,056 | 2,742 | 2,086 | 168 |  |
| Model 1 | 21.0 (21.0, 21.0) | 21.5 (21.5, 21.6) | 21.8 (21.5, 22.1) | 21.5 (21.3, 21.7) | 20.8 (20.6, 21.1) | 20.5 (19.7, 21.3) | <0.0001 |
| Model 2 | 21.1 (21.0, 21.1) | 21.5 (21.4, 21.5) | 21.6 (21.3, 22.0) | 21.4 (21.2, 21.6) | 20.7 (20.5, 20.9) | 20.4 (19.7, 21.2) | <0.0001 |
| Model 3 | 21.1 (21.1, 21.1)^a^ | 21.4 (21.3, 21.4)^b^ | 21.5 (21.2, 21.8)^ab^ | 21.3 (21.1, 21.5)^ab^ | 20.6 (20.4, 20.9)^c^ | 20.4 (19.6, 21.2)^abc^ | <0.0001 |
| Model 4 | 21.1 (21.1, 21.1) | 21.4 (21.3, 21.4) | 21.5 (21.2, 21.8) | 21.3 (21.1, 21.5) | 20.6 (20.4, 20.9) | 20.3 (19.6, 21.1) | <0.0001 |
| Arithmetic mean | 21.8 (21.8, 21.9) | 22.1 (22.0, 22.1) | 22.2 (21.9, 22.5) | 22.0 (21.8, 22.2) | 21.3 (21.1, 21.6) | 21.0 (20.2, 21.9) | <0.0001 |

1. Unless otherwise specified, all estimates represent adjusted geometric means and 95% CIs, or if stated, adjusted arithmetic means and 95% CIs. Model 1 was adjusted for age (5 years categories) and fasting status (0-1, 2, 3, 4, 5, 6-7, ≥8 hours); model 2 was additionally adjusted for BMI (<20, 20.0-22.4, 22.5-24.9, 25.0-27.4, 27.5-29.9, 30.0-32.4, 32.5-34.9, ≥35.0 kg/m^2^, unknown), model 3 was additionally adjusted for alcohol consumption (<1, 1-7, 8-15, ≥16 g/d, unknown) and smoking status (never, previous, current <15 cigarettes/day, current ≥15 cigarettes/day, unknown), model 4 was additionally adjusted for physical activity (low <10 excess MET hours/week, moderate 10-49 excess MET hours/week, high ≥50 excess MET hours/week, unknown). Arithmetic means were based on model 3 unless otherwise specified.
2. Includes participants who consume any red or processed meat, regardless of whether they consume poultry, fish, or dairy. Cut-offs of regular and low consumption determined based on consumption of red and processed meat (beef, lamb, pork, processed meat) as reported on the touchscreen questionnaire.
3. Represents p for heterogeneity across the six diet groups based on Wald tests. Groups that do not share a superscript letter (ordered alphabetically from left to right) were significantly different at the 5% level from post hoc Bonferroni-corrected pairwise comparisons after linear regression (model 3).

**Supplemental Table 15**: **Cancer-related** serum biomarker concentrations (numbers, adjusted means and 95% CIs)^1^ in **British Indian participants** by diet group in the UK Biobank.

| **Cancer-related serum biomarker** | **All participants** | | |  | **Women** | | |  | **Men** | | |
| --- | --- | --- | --- | --- | --- | --- | --- | --- | --- | --- | --- |
|  | **Meat-eaters** | **Vegetarians** | **p-het^2^** |  | **Meat-eaters** | **Vegetarians** | **p-het^2^** |  | **Meat-eaters** | **Vegetarians** | **p-het^2^** |
| **Sex hormone-binding globulin, nmol/L** |  |  |  |  |  |  |  |  |  |  |  |
| Participants, n | 3,476 | 1,189 |  |  | 1,436 | 765 |  |  | 2,040 | 424 |  |
| Model 1 | 36.1 (35.5, 36.7) | 35.5 (34.6, 36.5) | 0.31 |  | 43.6 (42.4, 44.7) | 43.2 (41.6, 44.8) | 0.70 |  | 30.4 (29.9, 31.0) | 30.2 (29.1, 31.4) | 0.71 |
| Model 2 | 36.2 (35.7, 36.7) | 35.3 (34.4, 36.2) | 0.10 |  | 43.6 (42.6, 44.7) | 43.0 (41.6, 44.5) | 0.50 |  | 30.5 (30.0, 31.0) | 30.0 (28.9, 31.1) | 0.40 |
| Model 3 | 36.0 (35.5, 36.6) | 35.7 (34.8, 36.7) | 0.61 |  | 43.4 (42.4, 44.5) | 43.4 (41.9, 44.9) | 0.96 |  | 30.5 (29.9, 31.0) | 30.1 (28.9, 31.3) | 0.56 |
| Model 4 | 36.0 (35.5, 36.6) | 35.8 (34.8, 36.8) | 0.69 |  | 43.4 (42.4, 44.5) | 43.4 (41.9, 44.9) | 0.97 |  | 30.4 (29.9, 31.0) | 30.1 (29.0, 31.3) | 0.65 |
| Arithmetic mean | 41.1 (40.4, 41.8) | 40.6 (39.4, 41.9) | 0.54 |  | 49.7 (48.3, 51.1) | 49.5 (47.6, 51.4) | 0.87 |  | 33.3 (32.8, 33.9) | 32.7 (31.4, 34.0) | 0.41 |
| **Testosterone, nmol/L** |  |  |  |  |  |  |  |  |  |  |  |
| Participants, n | 3,793 | 1,327 |  |  | 1,590 | 860 |  |  | 2,203 | 467 |  |
| Model 1 | 3.12 (3.07, 3.17) | 3.05 (2.97, 3.14) | 0.22 |  | 0.79 (0.77, 0.82) | 0.77 (0.74, 0.81) | 0.40 |  | 11.0 (10.8, 11.1) | 10.9 (10.6, 11.2) | 0.76 |
| Model 2 | 3.12 (3.07, 3.17) | 3.05 (2.97, 3.14) | 0.18 |  | 0.79 (0.77, 0.82) | 0.77 (0.74, 0.81) | 0.44 |  | 11.0 (10.8, 11.1) | 10.8 (10.5, 11.1) | 0.31 |
| Model 3 | 3.11 (3.06, 3.16) | 3.07 (2.98, 3.16) | 0.46 |  | 0.79 (0.76, 0.82) | 0.77 (0.74, 0.81) | 0.44 |  | 10.9 (10.8, 11.1) | 11.0 (10.7, 11.3) | 0.76 |
| Model 4 | 3.11 (3.06, 3.16) | 3.07 (2.98, 3.16) | 0.47 |  | 0.79 (0.76, 0.82) | 0.77 (0.74, 0.81) | 0.44 |  | 10.9 (10.8, 11.1) | 11.0 (10.7, 11.3) | 0.70 |
| Arithmetic mean | 6.45 (6.37, 6.53) | 6.43 (6.29, 6.57) | 0.76 |  | 0.97 (0.94, 1.00) | 0.94 (0.90, 0.99) | 0.35 |  | 11.5 (11.3, 11.6) | 11.4 (11.1, 11.8) | 0.82 |
| **Insulin-like growth factor I, nmol/L** |  |  |  |  |  |  |  |  |  |  |  |
| Participants, n | 3,799 | 1,338 |  |  | 1,593 | 867 |  |  | 2,206 | 471 |  |
| Model 1 | 19.6 (19.4, 19.8) | 18.0 (17.7, 18.3) | <0.0001 |  | 18.9 (18.7, 19.2) | 17.2 (16.8, 17.5) | <0.0001 |  | 20.2 (20.0, 20.5) | 19.1 (18.6, 19.6) | <0.0001 |
| Model 2 | 19.6 (19.4, 19.8) | 18.0 (17.7, 18.3) | <0.0001 |  | 18.9 (18.7, 19.2) | 17.2 (16.8, 17.5) | <0.0001 |  | 20.2 (20.0, 20.5) | 19.1 (18.6, 19.6) | <0.0001 |
| Model 3 | 19.6 (19.4, 19.8) | 18.0 (17.7, 18.3) | <0.0001 |  | 18.9 (18.6, 19.2) | 17.3 (16.9, 17.6) | <0.0001 |  | 20.3 (20.0, 20.5) | 18.9 (18.4, 19.4) | <0.0001 |
| Model 4 | 19.6 (19.4, 19.8) | 18.0 (17.7, 18.3) | <0.0001 |  | 18.9 (18.6, 19.2) | 17.3 (16.9, 17.6) | <0.0001 |  | 20.3 (20.0, 20.5) | 18.9 (18.4, 19.4) | <0.0001 |
| Arithmetic mean | 20.5 (20.3, 20.6) | 18.9 (18.6, 19.2) | <0.0001 |  | 19.8 (19.5, 20.0) | 18.2 (17.8, 18.5) | <0.0001 |  | 21.1 (20.9, 21.3) | 19.6 (19.1, 20.1) | <0.0001 |

1. Unless otherwise specified, all estimates represent adjusted geometric means and 95% CIs, or if stated, adjusted arithmetic means and 95% CIs. Model 1 was adjusted for sex (for analyses with all participants), age (5 years categories) and fasting status (0-1, 2, 3, 4, 5, 6-7, ≥8 hours); model 2 was additionally adjusted for BMI (<20, 20.0-22.4, 22.5-24.9, 25.0-27.4, 27.5-29.9, 30.0-32.4, 32.5-34.9, ≥35.0 kg/m^2^, unknown), model 3 was additionally adjusted for alcohol consumption (<1, 1-7, 8-15, ≥16 g/d, unknown) and smoking status (never, previous, current <15 cigarettes/day, current ≥15 cigarettes/day, unknown), model 4 was additionally adjusted for physical activity (low <10 excess MET hours/week, moderate 10-49 excess MET hours/week, high ≥50 excess MET hours/week, unknown). Arithmetic means were based on model 3 unless otherwise specified.
2. Represents p for heterogeneity across the two diet groups based on Wald tests.

**Supplemental Table 16**: **Diabetes-related** serum and packed red blood cell biomarker concentrations (numbers, adjusted means and 95% CIs)^1^ in **white British participants** by diet group in the UK Biobank.

| **Diabetes-related serum or packed red blood cell biomarker** | **Regular meat eaters (>3 times/week)^2^** | **Low meat eaters (>3 times/week)^2^** | **Poultry eaters** | **Fish eaters** | **Vegetarians** | **Vegans** | **p-het^3^** |
| --- | --- | --- | --- | --- | --- | --- | --- |
| **Glycated hemoglobin (HbA1c), mmol/mol** |  |  |  |  |  |  |  |
| Participants, n | 199,503 | 202,486 | 4,652 | 9,673 | 6,299 | 377 |  |
| Model 1 | 35.2 (35.2, 35.2) | 34.8 (34.8, 34.8) | 34.5 (34.4, 34.6) | 34.5 (34.4, 34.6) | 34.7 (34.6, 34.8) | 33.8 (33.4, 34.2) | <0.0001 |
| Model 2 | 35.1 (35.1, 35.1) | 34.9 (34.9, 34.9) | 34.8 (34.7, 34.9) | 34.8 (34.7, 34.9) | 34.9 (34.8, 35.0) | 34.1 (33.7, 34.5) | <0.0001 |
| Model 3 | 35.1 (35.1, 35.1)^a^ | 34.9 (34.8, 34.9)^b^ | 34.7 (34.6, 34.8)^b^ | 34.8 (34.7, 34.9)^b^ | 34.9 (34.8, 35.0)^b^ | 33.9 (33.5, 34.3)^c^ | <0.0001 |
| Model 4 | 35.1 (35.1, 35.1) | 34.9 (34.8, 34.9) | 34.7 (34.6, 34.8) | 34.8 (34.7, 34.9) | 34.9 (34.8, 35.0) | 33.9 (33.5, 34.3) | <0.0001 |
| Arithmetic mean | 35.4 (35.4, 35.4) | 35.1 (35.1, 35.2) | 35.0 (34.8, 35.1) | 35.1 (35.0, 35.2) | 35.2 (35.0, 35.3) | 34.2 (33.7, 34.7) | <0.0001 |
| **Glucose, mmol/L** |  |  |  |  |  |  |  |
| Participants, n | 183,267 | 185,786 | 4,259 | 8,890 | 5,762 | 361 |  |
| Model 1 | 4.97 (4.97, 4.98) | 4.95 (4.94, 4.95) | 4.92 (4.90, 4.94) | 4.90 (4.89, 4.92) | 4.92 (4.90, 4.93) | 4.86 (4.79, 4.93) | <0.0001 |
| Model 2 | 4.96 (4.96, 4.97) | 4.95 (4.95, 4.96) | 4.95 (4.93, 4.97) | 4.94 (4.92, 4.95) | 4.95 (4.93, 4.97) | 4.91 (4.84, 4.98) | <0.0001 |
| Model 3 | 4.96 (4.96, 4.96)^a^ | 4.95 (4.95, 4.96)^ab^ | 4.95 (4.93, 4.97)^ab^ | 4.94 (4.93, 4.95)^b^ | 4.95 (4.93, 4.97)^ab^ | 4.91 (4.85, 4.99)^ab^ | 0.002 |
| Model 4 | 4.96 (4.96, 4.96) | 4.95 (4.95, 4.96) | 4.95 (4.93, 4.97) | 4.94 (4.93, 4.95) | 4.95 (4.93, 4.97) | 4.92 (4.85, 4.99) | 0.005 |
| Arithmetic mean | 5.02 (5.01, 5.02) | 5.01 (5.00, 5.01) | 5.00 (4.98, 5.03) | 4.99 (4.97, 5.01) | 5.01 (4.99, 5.03) | 4.96 (4.87, 5.05) | 0.001 |

1. Unless otherwise specified, all estimates represent adjusted geometric means and 95% CIs, or if stated, adjusted arithmetic means and 95% CIs. Model 1 was adjusted for sex, age (5 years categories) and fasting status (0-1, 2, 3, 4, 5, 6-7, ≥8 hours); model 2 was additionally adjusted for BMI (<20, 20.0-22.4, 22.5-24.9, 25.0-27.4, 27.5-29.9, 30.0-32.4, 32.5-34.9, ≥35.0 kg/m^2^, unknown), model 3 was additionally adjusted for alcohol consumption (<1, 1-7, 8-15, ≥16 g/d, unknown) and smoking status (never, previous, current <15 cigarettes/day, current ≥15 cigarettes/day, unknown), model 4 was additionally adjusted for physical activity (low <10 excess MET hours/week, moderate 10-49 excess MET hours/week, high ≥50 excess MET hours/week, unknown). Arithmetic means were based on model 3 unless otherwise specified.
2. Includes participants who consume any red or processed meat, regardless of whether they consume poultry, fish, or dairy. Cut-offs of regular and low consumption determined based on consumption of red and processed meat (beef, lamb, pork, processed meat) as reported on the touchscreen questionnaire.
3. Represents p for heterogeneity across the six diet groups based on Wald tests. ﻿Groups that do not share a superscript letter (ordered alphabetically from left to right) were significantly different at the 5% level from post hoc Bonferroni-corrected pairwise comparisons after linear regression (model 3).

**Supplemental Table 17**: **Diabetes-related** serum and packed red blood cell biomarker concentrations (numbers, adjusted means and 95% CIs)^1^ in **white British women** by diet group in the UK Biobank.

| **Diabetes-related serum or packed red blood cell biomarker** | **Regular meat eaters (>3 times/week)^2^** | **Low meat eaters (>3 times/week)^2^** | **Poultry eaters** | **Fish eaters** | **Vegetarians** | **Vegans** | **p-het^3^** |
| --- | --- | --- | --- | --- | --- | --- | --- |
| **Glycated hemoglobin (HbA1c), mmol/mol** |  |  |  |  |  |  |  |
| Participants, n | 87,046 | 130,030 | 3,635 | 6,991 | 4,262 | 221 |  |
| Model 1 | 35.1 (35.1, 35.2) | 34.8 (34.8, 34.8) | 34.6 (34.5, 34.8) | 34.6 (34.5, 34.7) | 34.8 (34.7, 34.9) | 33.9 (33.4, 34.4) | <0.0001 |
| Model 2 | 35.0 (35.0, 35.1) | 34.9 (34.8, 34.9) | 34.8 (34.7, 35.0) | 34.9 (34.8, 35.0) | 35.0 (34.9, 35.1) | 34.1 (33.7, 34.6) | <0.0001 |
| Model 3 | 35.1 (35.0, 35.1)^a^ | 34.8 (34.8, 34.9)^b^ | 34.8 (34.6, 34.9)^b^ | 34.9 (34.8, 35.0)^b^ | 35.0 (34.9, 35.1)^ab^ | 34.0 (33.5, 34.5)^c^ | <0.0001 |
| Model 4 | 35.1 (35.0, 35.1) | 34.8 (34.8, 34.9) | 34.8 (34.6, 34.9) | 34.9 (34.8, 35.0) | 35.0 (34.9, 35.1) | 34.0 (33.5, 34.5) | <0.0001 |
| Arithmetic mean | 35.3 (35.3, 35.3) | 35.1 (35.1, 35.1) | 35.0 (34.9, 35.1) | 35.1 (35.0, 35.2) | 35.2 (35.1, 35.3) | 34.2 (33.7, 34.8) | <0.0001 |
| **Glucose, mmol/L** |  |  |  |  |  |  |  |
| Participants, n | 79,174 | 118,655 | 3,312 | 6,396 | 3,872 | 211 |  |
| Model 1 | 4.96 (4.96, 4.96) | 4.94 (4.94, 4.94) | 4.90 (4.88, 4.92) | 4.90 (4.89, 4.92) | 4.91 (4.89, 4.93) | 4.86 (4.77, 4.95) | <0.0001 |
| Model 2 | 4.95 (4.95, 4.95) | 4.94 (4.94, 4.95) | 4.93 (4.90, 4.95) | 4.93 (4.91, 4.94) | 4.94 (4.92, 4.96) | 4.90 (4.81, 4.98) | 0.018 |
| Model 3 | 4.95 (4.94, 4.95)^a^ | 4.94 (4.94, 4.95)^a^ | 4.93 (4.91, 4.95)^a^ | 4.93 (4.91, 4.94)^a^ | 4.94 (4.92, 4.96)^a^ | 4.90 (4.82, 4.99)^a^ | 0.10 |
| Model 4 | 4.95 (4.94, 4.95) | 4.95 (4.94, 4.95) | 4.93 (4.91, 4.95) | 4.93 (4.91, 4.95) | 4.94 (4.92, 4.96) | 4.90 (4.82, 4.99) | 0.11 |
| Arithmetic mean | 5.00 (4.99, 5.00) | 4.99 (4.99, 5.00) | 4.97 (4.95, 5.00) | 4.98 (4.96, 5.00) | 4.99 (4.97, 5.01) | 4.95 (4.85, 5.05) | 0.08 |

1. Unless otherwise specified, all estimates represent adjusted geometric means and 95% CIs, or if stated, adjusted arithmetic means and 95% CIs. Model 1 was adjusted for age (5 years categories) and fasting status (0-1, 2, 3, 4, 5, 6-7, ≥8 hours); model 2 was additionally adjusted for BMI (<20, 20.0-22.4, 22.5-24.9, 25.0-27.4, 27.5-29.9, 30.0-32.4, 32.5-34.9, ≥35.0 kg/m^2^, unknown), model 3 was additionally adjusted for alcohol consumption (<1, 1-7, 8-15, ≥16 g/d, unknown) and smoking status (never, previous, current <15 cigarettes/day, current ≥15 cigarettes/day, unknown), model 4 was additionally adjusted for physical activity (low <10 excess MET hours/week, moderate 10-49 excess MET hours/week, high ≥50 excess MET hours/week, unknown). Arithmetic means were based on model 3 unless otherwise specified.
2. Includes participants who consume any red or processed meat, regardless of whether they consume poultry, fish, or dairy. Cut-offs of regular and low consumption determined based on consumption of red and processed meat (beef, lamb, pork, processed meat) as reported on the touchscreen questionnaire.
3. Represents p for heterogeneity across the six diet groups based on Wald tests. Groups that do not share a superscript letter (ordered alphabetically from left to right) were significantly different at the 5% level from post hoc Bonferroni-corrected pairwise comparisons after linear regression (model 3).

**Supplemental Table 18**: **Diabetes-related** serum and packed red blood cell biomarker concentrations (numbers, adjusted means and 95% CIs)^1^ in **white British men** by diet group in the UK Biobank.

| **Diabetes-related serum or packed red blood cell biomarker** | **Regular meat eaters (>3 times/week)^2^** | **Low meat eaters (>3 times/week)^2^** | **Poultry eaters** | **Fish eaters** | **Vegetarians** | **Vegans** | **p-het^3^** |
| --- | --- | --- | --- | --- | --- | --- | --- |
| **Glycated hemoglobin (HbA1c), mmol/mol** |  |  |  |  |  |  |  |
| Participants, n | 112,457 | 72,456 | 1,017 | 2,682 | 2,037 | 156 |  |
| Model 1 | 35.2 (35.2, 35.3) | 34.8 (34.8, 34.8) | 34.1 (33.8, 34.4) | 34.2 (34.1, 34.4) | 34.4 (34.2, 34.6) | 33.6 (32.9, 34.2) | <0.0001 |
| Model 2 | 35.2 (35.1, 35.2) | 34.9 (34.9, 34.9) | 34.4 (34.2, 34.7) | 34.6 (34.4, 34.7) | 34.8 (34.6, 34.9) | 34.0 (33.4, 34.7) | <0.0001 |
| Model 3 | 35.2 (35.1, 35.2)^a^ | 34.9 (34.9, 34.9)^b^ | 34.4 (34.1, 34.6)^c^ | 34.6 (34.4, 34.8)^c^ | 34.7 (34.5, 34.9)^bc^ | 33.9 (33.2, 34.5)^c^ | <0.0001 |
| Model 4 | 35.2 (35.1, 35.2) | 34.9 (34.9, 34.9) | 34.4 (34.1, 34.6) | 34.6 (34.5, 34.8) | 34.7 (34.5, 34.9) | 33.9 (33.2, 34.5) | <0.0001 |
| Arithmetic mean | 35.5 (35.4, 35.5) | 35.2 (35.2, 35.2) | 34.7 (34.4, 35.0) | 34.9 (34.7, 35.1) | 35.0 (34.8, 35.3) | 34.2 (33.4, 35.0) | <0.0001 |
| **Glucose, mmol/L** |  |  |  |  |  |  |  |
| Participants, n | 104,093 | 67,131 | 947 | 2,494 | 1,890 | 150 |  |
| Model 1 | 4.98 (4.98, 4.99) | 4.95 (4.95, 4.96) | 4.95 (4.90, 5.00) | 4.91 (4.88, 4.94) | 4.93 (4.89, 4.96) | 4.85 (4.74, 4.97) | <0.0001 |
| Model 2 | 4.98 (4.97, 4.98) | 4.96 (4.96, 4.97) | 4.99 (4.95, 5.04) | 4.95 (4.93, 4.98) | 4.97 (4.94, 5.00) | 4.92 (4.80, 5.03) | 0.001 |
| Model 3 | 4.98 (4.97, 4.98)^a^ | 4.96 (4.96, 4.97)^a^ | 5.00 (4.96, 5.05)^a^ | 4.96 (4.93, 4.98)^a^ | 4.97 (4.94, 5.01)^a^ | 4.92 (4.81, 5.04)^a^ | 0.03 |
| Model 4 | 4.98 (4.97, 4.98) | 4.96 (4.96, 4.97) | 5.00 (4.96, 5.05) | 4.96 (4.93, 4.99) | 4.98 (4.94, 5.01) | 4.93 (4.81, 5.04) | 0.03 |
| Arithmetic mean | 5.04 (5.03, 5.04) | 5.02 (5.02, 5.03) | 5.06 (5.01, 5.12) | 5.01 (4.97, 5.04) | 5.04 (4.99, 5.08) | 4.97 (4.82, 5.12) | 0.02 |

1. Unless otherwise specified, all estimates represent adjusted geometric means and 95% CIs, or if stated, adjusted arithmetic means and 95% CIs. Model 1 was adjusted for age (5 years categories) and fasting status (0-1, 2, 3, 4, 5, 6-7, ≥8 hours); model 2 was additionally adjusted for BMI (<20, 20.0-22.4, 22.5-24.9, 25.0-27.4, 27.5-29.9, 30.0-32.4, 32.5-34.9, ≥35.0 kg/m^2^, unknown), model 3 was additionally adjusted for alcohol consumption (<1, 1-7, 8-15, ≥16 g/d, unknown) and smoking status (never, previous, current <15 cigarettes/day, current ≥15 cigarettes/day, unknown), model 4 was additionally adjusted for physical activity (low <10 excess MET hours/week, moderate 10-49 excess MET hours/week, high ≥50 excess MET hours/week, unknown). Arithmetic means were based on model 3 unless otherwise specified.
2. Includes participants who consume any red or processed meat, regardless of whether they consume poultry, fish, or dairy. Cut-offs of regular and low consumption determined based on consumption of red and processed meat (beef, lamb, pork, processed meat) as reported on the touchscreen questionnaire.
3. Represents p for heterogeneity across the six diet groups based on Wald tests. Groups that do not share a superscript letter (ordered alphabetically from left to right) were significantly different at the 5% level from post hoc Bonferroni-corrected pairwise comparisons after linear regression (model 3).

**Supplemental Table 19**: **Diabetes-related** serum and packed red blood cell biomarker concentrations (numbers, adjusted means and 95% CIs)^1^ in **British Indian participants** by diet group in the UK Biobank.

| **Diabetes-related serum or packed red blood cell biomarker** | **All participants** | | |  | **Women** | | |  | **Men** | | |
| --- | --- | --- | --- | --- | --- | --- | --- | --- | --- | --- | --- |
|  | **Meat-eaters** | **Vegetarians** | **p-het^2^** |  | **Meat-eaters** | **Vegetarians** | **p-het^2^** |  | **Meat-eaters** | **Vegetarians** | **p-het^2^** |
| **Glycated hemoglobin (HbA1c), mmol/mol** |  |  |  |  |  |  |  |  |  |  |  |
| Participants, n | 3,290 | 1,158 |  |  | 1,437 | 762 |  |  | 1,853 | 396 |  |
| Model 1 | 37.4 (37.2, 37.6) | 37.6 (37.3, 38.0) | 0.23 |  | 37.2 (36.9, 37.4) | 37.8 (37.4, 38.1) | 0.004 |  | 37.7 (37.4, 37.9) | 37.2 (36.6, 37.7) | 0.13 |
| Model 2 | 37.4 (37.2, 37.6) | 37.7 (37.4, 38.0) | 0.16 |  | 37.2 (36.9, 37.4) | 37.8 (37.5, 38.1) | 0.003 |  | 37.6 (37.4, 37.9) | 37.2 (36.7, 37.8) | 0.20 |
| Model 3 | 37.5 (37.3, 37.7) | 37.4 (37.1, 37.8) | 0.81 |  | 37.3 (37.0, 37.5) | 37.6 (37.3, 38.0) | 0.09 |  | 37.7 (37.4, 38.0) | 36.9 (36.3, 37.5) | 0.02 |
| Model 4 | 37.5 (37.3, 37.7) | 37.4 (37.1, 37.8) | 0.83 |  | 37.3 (37.0, 37.5) | 37.6 (37.3, 38.0) | 0.09 |  | 37.7 (37.4, 38.0) | 36.9 (36.3, 37.5) | 0.02 |
| Arithmetic mean | 37.9 (37.7, 38.1) | 37.9 (37.6, 38.3) | 0.96 |  | 37.6 (37.3, 37.9) | 38.1 (37.7, 38.5) | 0.06 |  | 38.2 (37.9, 38.5) | 37.4 (36.7, 38.1) | 0.03 |
| **Glucose, mmol/L** |  |  |  |  |  |  |  |  |  |  |  |
| Participants, n | 3,042 | 1,049 |  |  | 1,307 | 692 |  |  | 1,735 | 357 |  |
| Model 1 | 4.97 (4.94, 5.00) | 4.95 (4.91, 5.00) | 0.53 |  | 4.96 (4.92, 5.00) | 4.94 (4.89, 5.00) | 0.54 |  | 4.98 (4.94, 5.02) | 4.96 (4.87, 5.04) | 0.60 |
| Model 2 | 4.97 (4.94, 5.00) | 4.96 (4.91, 5.01) | 0.64 |  | 4.96 (4.92, 5.00) | 4.95 (4.90, 5.00) | 0.73 |  | 4.98 (4.94, 5.02) | 4.96 (4.88, 5.05) | 0.67 |
| Model 3 | 4.97 (4.95, 5.00) | 4.95 (4.90, 5.00) | 0.35 |  | 4.97 (4.93, 5.01) | 4.94 (4.88, 4.99) | 0.45 |  | 4.98 (4.94, 5.02) | 4.96 (4.86, 5.05) | 0.60 |
| Model 4 | 4.97 (4.95, 5.00) | 4.95 (4.90, 5.00) | 0.38 |  | 4.97 (4.93, 5.01) | 4.94 (4.88, 5.00) | 0.48 |  | 4.98 (4.94, 5.02) | 4.96 (4.87, 5.05) | 0.62 |
| Arithmetic mean | 5.04 (5.01, 5.08) | 5.02 (4.96, 5.09) | 0.59 |  | 5.02 (4.97, 5.07) | 5.01 (4.94, 5.08) | 0.75 |  | 5.07 (5.01, 5.12) | 5.04 (4.91, 5.16) | 0.70 |

1. Unless otherwise specified, all estimates represent adjusted geometric means and 95% CIs, or if stated, adjusted arithmetic means and 95% CIs. Model 1 was adjusted for sex (for analyses with all participants), age (5 years categories) and fasting status (0-1, 2, 3, 4, 5, 6-7, ≥8 hours); model 2 was additionally adjusted for BMI (<20, 20.0-22.4, 22.5-24.9, 25.0-27.4, 27.5-29.9, 30.0-32.4, 32.5-34.9, ≥35.0 kg/m^2^, unknown), model 3 was additionally adjusted for alcohol consumption (<1, 1-7, 8-15, ≥16 g/d, unknown) and smoking status (never, previous, current <15 cigarettes/day, current ≥15 cigarettes/day, unknown), model 4 was additionally adjusted for physical activity (low <10 excess MET hours/week, moderate 10-49 excess MET hours/week, high ≥50 excess MET hours/week, unknown). Arithmetic means were based on model 3 unless otherwise specified.
2. Represents p for heterogeneity across the two diet groups based on Wald tests.

**Supplemental Table 20**: **Renal-related** serum and urinary biomarker concentrations (numbers, adjusted means and 95% CIs)^1^ in **white British participants** by diet group in the UK Biobank.

| **Renal-related serum or urinary biomarker** | **Regular meat eaters (>3 times/week)^2^** | **Low meat eaters (>3 times/week)^2^** | **Poultry eaters** | **Fish eaters** | **Vegetarians** | **Vegans** | **p-het^3^** |
| --- | --- | --- | --- | --- | --- | --- | --- |
| **Cystatin C, mg/L** |  |  |  |  |  |  |  |
| Participants, n | 208,720 | 209,277 | 4,768 | 9,920 | 6,441 | 399 |  |
| Model 1 | 0.90 (0.90, 0.90) | 0.89 (0.89, 0.89) | 0.88 (0.88, 0.89) | 0.89 (0.89, 0.89) | 0.92 (0.92, 0.92) | 0.91 (0.90, 0.92) | <0.0001 |
| Model 2 | 0.89 (0.89, 0.90) | 0.89 (0.89, 0.89) | 0.90 (0.89, 0.90) | 0.90 (0.90, 0.91) | 0.93 (0.93, 0.94) | 0.93 (0.92, 0.95) | <0.0001 |
| Model 3 | 0.90 (0.89, 0.90)^a^ | 0.89 (0.89, 0.89)^b^ | 0.89 (0.89, 0.90)^ab^ | 0.90 (0.90, 0.91)^c^ | 0.93 (0.93, 0.93)^d^ | 0.92 (0.91, 0.93)^cd^ | <0.0001 |
| Model 4 | 0.90 (0.89, 0.90) | 0.89 (0.89, 0.89) | 0.89 (0.89, 0.90) | 0.90 (0.90, 0.91) | 0.93 (0.93, 0.93) | 0.92 (0.91, 0.94) | <0.0001 |
| Arithmetic mean | 0.91 (0.91, 0.91) | 0.91 (0.90, 0.91) | 0.90 (0.90, 0.91) | 0.92 (0.91, 0.92) | 0.94 (0.94, 0.95) | 0.93 (0.92, 0.95) | <0.0001 |
| **Serum creatinine, µmol/L** |  |  |  |  |  |  |  |
| Participants, n | 208,631 | 209,195 | 4,763 | 9,906 | 6,444 | 399 |  |
| Model 1 | 71.2 (71.2, 71.3) | 70.5 (70.5, 70.6) | 68.3 (68.0, 68.6) | 66.6 (66.4, 66.8) | 64.5 (64.3, 64.8) | 62.2 (61.2, 63.2) | <0.0001 |
| Model 2 | 71.1 (71.1, 71.2) | 70.6 (70.5, 70.6) | 68.8 (68.5, 69.1) | 67.1 (66.8, 67.3) | 65.0 (64.7, 65.2) | 62.9 (61.9, 63.9) | <0.0001 |
| Model 3 | 71.2 (71.2, 71.3)^a^ | 70.5 (70.5, 70.6)^b^ | 68.6 (68.3, 68.9)^c^ | 66.9 (66.7, 67.1)^d^ | 64.8 (64.5, 65.1)^e^ | 62.5 (61.5, 63.6)^f^ | <0.0001 |
| Model 4 | 71.2 (71.2, 71.3) | 70.5 (70.5, 70.6) | 68.6 (68.3, 68.9) | 66.9 (66.7, 67.1) | 64.8 (64.5, 65.0) | 62.5 (61.5, 63.5) | <0.0001 |
| Arithmetic mean | 72.4 (72.4, 72.4) | 72.4 (72.3, 72.4) | 72.1 (72.0, 72.2) | 72.0 (71.9, 72.1) | 71.9 (71.8, 72.0) | 71.5 (71.1, 71.9) | <0.0001 |
| **Total protein, g/L** |  |  |  |  |  |  |  |
| Participants, n | 191,220 | 191,295 | 4,318 | 8,997 | 5,852 | 368 |  |
| Model 1 | 72.3 (72.3, 72.3) | 72.2 (72.2, 72.3) | 72.0 (71.9, 72.1) | 71.9 (71.8, 72.0) | 71.8 (71.7, 71.9) | 71.3 (70.9, 71.7) | <0.0001 |
| Model 2 | 72.3 (72.3, 72.3) | 72.2 (72.2, 72.3) | 72.0 (71.9, 72.2) | 71.9 (71.9, 72.0) | 71.9 (71.8, 72.0) | 71.4 (71.0, 71.8) | <0.0001 |
| Model 3 | 72.3 (72.3, 72.3)^a^ | 72.2 (72.2, 72.3)^b^ | 72.0 (71.9, 72.1)^c^ | 71.9 (71.8, 72.0)^cd^ | 71.8 (71.7, 71.9)^cd^ | 71.4 (71.0, 71.8)^d^ | <0.0001 |
| Model 4 | 72.3 (72.3, 72.3) | 72.2 (72.2, 72.3) | 72.0 (71.9, 72.1) | 71.9 (71.8, 72.0) | 71.8 (71.7, 71.9) | 71.4 (71.0, 71.8) | <0.0001 |
| Arithmetic mean | 72.4 (72.4, 72.4) | 72.4 (72.3, 72.4) | 72.1 (72.0, 72.2) | 72.0 (71.9, 72.1) | 71.9 (71.8, 72.0) | 71.5 (71.1, 71.9) | <0.0001 |
| **Urea, mmol/L** |  |  |  |  |  |  |  |
| Participants, n | 208,583 | 209,160 | 4,761 | 9,906 | 6,445 | 398 |  |
| Model 1 | 5.36 (5.36, 5.37) | 5.24 (5.23, 5.24) | 4.92 (4.89, 4.95) | 4.70 (4.68, 4.73) | 4.49 (4.46, 4.51) | 4.17 (4.08, 4.27) | <0.0001 |
| Model 2 | 5.35 (5.35, 5.36) | 5.24 (5.24, 5.25) | 4.96 (4.93, 4.99) | 4.74 (4.72, 4.77) | 4.53 (4.50, 4.55) | 4.23 (4.13, 4.33) | <0.0001 |
| Model 3 | 5.36 (5.36, 5.37)^a^ | 5.23 (5.23, 5.24)^b^ | 4.95 (4.91, 4.98)^c^ | 4.73 (4.71, 4.75)^d^ | 4.51 (4.48, 4.54)^e^ | 4.21 (4.11, 4.30)^f^ | <0.0001 |
| Model 4 | 5.36 (5.36, 5.37) | 5.23 (5.23, 5.24) | 4.94 (4.91, 4.97) | 4.73 (4.71, 4.75) | 4.51 (4.48, 4.53) | 4.20 (4.11, 4.30) | <0.0001 |
| Arithmetic mean | 5.52 (5.52, 5.53) | 5.39 (5.39, 5.40) | 5.11 (5.08, 5.15) | 4.90 (4.87, 4.92) | 4.69 (4.66, 4.73) | 4.43 (4.30, 4.56) | <0.0001 |
| **Phosphate, mmol/L** |  |  |  |  |  |  |  |
| Participants, n | 191,115 | 191,103 | 4,312 | 8,989 | 5,849 | 368 |  |
| Model 1 | 1.15 (1.15, 1.15) | 1.15 (1.15, 1.15) | 1.15 (1.14, 1.15) | 1.15 (1.15, 1.15) | 1.15 (1.15, 1.16) | 1.12 (1.11, 1.14) | <0.0001 |
| Model 2 | 1.15 (1.15, 1.15) | 1.15 (1.14, 1.15) | 1.14 (1.14, 1.15) | 1.14 (1.14, 1.15) | 1.15 (1.14, 1.15) | 1.12 (1.10, 1.13) | <0.0001 |
| Model 3 | 1.15 (1.15, 1.15)^a^ | 1.15 (1.15, 1.15)^a^ | 1.14 (1.14, 1.15)^a^ | 1.14 (1.14, 1.15)^a^ | 1.15 (1.15, 1.15)^a^ | 1.12 (1.10, 1.13)^b^ | <0.0001 |
| Model 4 | 1.15 (1.15, 1.15) | 1.15 (1.15, 1.15) | 1.14 (1.14, 1.15) | 1.14 (1.14, 1.15) | 1.15 (1.14, 1.15) | 1.12 (1.10, 1.13) | <0.0001 |
| Arithmetic mean | 1.16 (1.16, 1.16) | 1.16 (1.16, 1.16) | 1.15 (1.15, 1.16) | 1.16 (1.15, 1.16) | 1.16 (1.16, 1.17) | 1.13 (1.12, 1.15) | <0.0001 |
| **Urate, µmol/L** |  |  |  |  |  |  |  |
| Participants, n | 208,504 | 209,026 | 4,760 | 9,904 | 6,435 | 398 |  |
| Model 1 | 304 (303, 304) | 296 (296, 296) | 280 (279, 282) | 278 (276, 279) | 272 (271, 274) | 291 (284, 297) | <0.0001 |
| Model 2 | 301 (301, 301) | 297 (297, 298) | 290 (288, 292) | 288 (287, 289) | 282 (280, 283) | 307 (301, 313) | <0.0001 |
| Model 3 | 301 (300, 301)^a^ | 298 (298, 298)^b^ | 292 (290, 294)^c^ | 289 (287, 290)^d^ | 283 (282, 284)^e^ | 310 (304, 317)^f^ | <0.0001 |
| Model 4 | 301 (300, 301) | 298 (298, 298) | 292 (290, 294) | 289 (287, 290) | 283 (282, 284) | 311 (304, 317) | <0.0001 |
| Arithmetic mean | 311 (311, 311) | 308 (308, 308) | 303 (301, 305) | 300 (298, 301) | 294 (293, 296) | 319 (313, 325) | <0.0001 |
| **Urinary creatinine, µmol/L** |  |  |  |  |  |  |  |
| Participants, n | 215,198 | 215,919 | 4,905 | 10,211 | 6,605 | 412 |  |
| Model 1 | 7,380 (7,360, 7,400) | 6,960 (6,940, 6,980) | 6,220 (6,110, 6,330) | 5,940 (5,870, 6,010) | 5,870 (5,780, 5,960) | 5,030 (4,730, 5,340) | <0.0001 |
| Model 2 | 7,280 (7,260, 7,300) | 7,010 (7,000, 7,030) | 6,560 (6,440, 6,670) | 6,280 (6,210, 6,360) | 6,180 (6,090, 6,270) | 5,430 (5,110, 5,760) | <0.0001 |
| Model 3 | 7,280 (7,260, 7,300)^a^ | 7,010 (7,000, 7,030)^b^ | 6,560 (6,450, 6,670)^c^ | 6,300 (6,220, 6,370)^d^ | 6,190 (6,090, 6,280)^d^ | 5,440 (5,120, 5,770)^e^ | <0.0001 |
| Model 4 | 7,280 (7,260, 7,300) | 7,020 (7,000, 7,030) | 6,570 (6,460, 6,690) | 6,310 (6,230, 6,380) | 6,190 (6,100, 6,290) | 5,450 (5,130, 5,780) | <0.0001 |
| Arithmetic mean | 8,960 (8,940, 8,990) | 8,710 (8,690, 8,730) | 8,420 (8,270, 8,570) | 8,060 (7,960, 8,160) | 8,000 (7,870, 8,130) | 7,370 (6,850, 7,880) | <0.0001 |
| **Urinary sodium/ creatinine ratio^4^** |  |  |  |  |  |  |  |
| Participants, n | 214,872 | 215,364 | 4,873 | 10,162 | 6,579 | 406 |  |
| Arithmetic mean, model 1 | 10.8 (10.8, 10.8) | 10.5 (10.4, 10.5) | 10.3 (10.1, 10.5) | 11.0 (10.9, 11.2) | 11.6 (11.4, 11.8) | 12.0 (11.3, 12.6) | <0.0001 |
| Arithmetic mean, model 2 | 10.8 (10.8, 10.9) | 10.5 (10.4, 10.5) | 10.2 (10.0, 10.4) | 11.0 (10.9, 11.1) | 11.6 (11.4, 11.7) | 11.9 (11.2, 12.6) | <0.0001 |
| Arithmetic mean, model 3 | 10.8 (10.8, 10.9)^a^ | 10.5 (10.4, 10.5)^b^ | 10.2 (10.0, 10.4)^b^ | 11.0 (10.9, 11.1)^a^ | 11.5 (11.4, 11.7)^c^ | 11.8 (11.1, 12.5)^ac^ | <0.0001 |
| Arithmetic mean, model 4 | 10.8 (10.8, 10.8) | 10.5 (10.4, 10.5) | 10.2 (10.0, 10.4) | 11.0 (10.9, 11.1) | 11.6 (11.4, 11.7) | 11.8 (11.1, 12.5) | <0.0001 |
| **Urinary potassium/ creatinine ratio^4^** |  |  |  |  |  |  |  |
| Participants, n | 214,751 | 215,438 | 4,892 | 10,181 | 6,582 | 411 |  |
| Arithmetic mean, model 1 | 8.23 (8.21, 8.24) | 8.60 (8.59, 8.62) | 9.29 (9.18, 9.41) | 9.67 (9.59, 9.75) | 9.80 (9.70, 9.90) | 10.2 (9.84, 10.6) | <0.0001 |
| Arithmetic mean, model 2 | 8.29 (8.27, 8.30) | 8.57 (8.55, 8.58) | 9.06 (8.94, 9.17) | 9.41 (9.33, 9.49) | 9.57 (9.47, 9.67) | 9.89 (9.49, 10.3) | <0.0001 |
| Arithmetic mean, model 3 | 8.26 (8.25, 8.28)^a^ | 8.58 (8.57, 8.60)^b^ | 9.15 (9.03, 9.26)^c^ | 9.42 (9.34, 9.50)^d^ | 9.64 (9.54, 9.73)^e^ | 10.1 (9.67, 10.5)^e^ | <0.0001 |
| Arithmetic mean, model 4 | 8.27 (8.25, 8.29) | 8.58 (8.57, 8.60) | 9.12 (9.01, 9.24) | 9.40 (9.32, 9.48) | 9.62 (9.52, 9.72) | 10.0 (9.65, 10.4) | <0.0001 |

1. Unless otherwise specified, all estimates represent adjusted geometric means and 95% CIs, or if stated, adjusted arithmetic means and 95% CIs. Model 1 was adjusted for sex, age (5 years categories) and fasting status (0-1, 2, 3, 4, 5, 6-7, ≥8 hours); model 2 was additionally adjusted for BMI (<20, 20.0-22.4, 22.5-24.9, 25.0-27.4, 27.5-29.9, 30.0-32.4, 32.5-34.9, ≥35.0 kg/m^2^, unknown), model 3 was additionally adjusted for alcohol consumption (<1, 1-7, 8-15, ≥16 g/d, unknown) and smoking status (never, previous, current <15 cigarettes/day, current ≥15 cigarettes/day, unknown), model 4 was additionally adjusted for physical activity (low <10 excess MET hours/week, moderate 10-49 excess MET hours/week, high ≥50 excess MET hours/week, unknown). Arithmetic means were based on model 3 unless otherwise specified.
2. Includes participants who consume any red or processed meat, regardless of whether they consume poultry, fish, or dairy. Cut-offs of regular and low consumption determined based on consumption of red and processed meat (beef, lamb, pork, processed meat) as reported on the touchscreen questionnaire.
3. Represents p for heterogeneity across the six diet groups based on Wald tests. Groups that do not share a superscript letter (ordered alphabetically from left to right) were significantly different at the 5% level from post hoc Bonferroni-corrected pairwise comparisons after linear regression (model 3).
4. Expressed as per mmol/L of urinary sodium or potassium to per mmol/L of urinary creatinine.

**Supplemental Table 21**: **Renal-related** serum and urinary biomarker concentrations (numbers, adjusted means and 95% CIs)^1^ in **white British women** by diet group in the UK Biobank.

| **Renal-related serum or urinary biomarker** | **Regular meat eaters (>3 times/week)^2^** | **Low meat eaters (>3 times/week)^2^** | **Poultry eaters** | **Fish eaters** | **Vegetarians** | **Vegans** | **p-het^3^** |
| --- | --- | --- | --- | --- | --- | --- | --- |
| **Cystatin C, mg/L** |  |  |  |  |  |  |  |
| Participants, n | 89,775 | 133,334 | 3,705 | 7,158 | 4,345 | 230 |  |
| Model 1 | 0.87 (0.87, 0.87) | 0.86 (0.86, 0.86) | 0.85 (0.85, 0.86) | 0.86 (0.86, 0.86) | 0.89 (0.89, 0.90) | 0.88 (0.87, 0.90) | <0.0001 |
| Model 2 | 0.86 (0.86, 0.87) | 0.86 (0.86, 0.87) | 0.87 (0.86, 0.87) | 0.88 (0.87, 0.88) | 0.91 (0.90, 0.91) | 0.90 (0.89, 0.92) | <0.0001 |
| Model 3 | 0.87 (0.86, 0.87)^a^ | 0.86 (0.86, 0.87)^a^ | 0.86 (0.86, 0.87)^a^ | 0.88 (0.87, 0.88)^b^ | 0.90 (0.90, 0.91)^c^ | 0.89 (0.88, 0.91)^bc^ | <0.0001 |
| Model 4 | 0.87 (0.86, 0.87) | 0.86 (0.86, 0.87) | 0.86 (0.86, 0.87) | 0.88 (0.87, 0.88) | 0.90 (0.90, 0.91) | 0.90 (0.88, 0.91) | <0.0001 |
| Arithmetic mean | 0.88 (0.88, 0.88) | 0.88 (0.88, 0.88) | 0.87 (0.87, 0.88) | 0.89 (0.88, 0.89) | 0.91 (0.91, 0.92) | 0.91 (0.89, 0.92) | <0.0001 |
| **Serum creatinine, µmol/L** |  |  |  |  |  |  |  |
| Participants, n | 89,751 | 133,275 | 3,700 | 7,147 | 4,348 | 230 |  |
| Model 1 | 64.0 (63.9, 64.0) | 63.4 (63.4, 63.5) | 61.5 (61.2, 61.9) | 60.5 (60.2, 60.7) | 58.9 (58.6, 59.2) | 57.1 (55.9, 58.3) | <0.0001 |
| Model 2 | 63.8 (63.8, 63.9) | 63.4 (63.4, 63.5) | 61.9 (61.6, 62.2) | 60.9 (60.6, 61.1) | 59.3 (59.0, 59.5) | 57.6 (56.4, 58.8) | <0.0001 |
| Model 3 | 63.9 (63.8, 64.0)^a^ | 63.4 (63.4, 63.5)^b^ | 61.8 (61.5, 62.1)^c^ | 60.8 (60.6, 61.0)^d^ | 59.1 (58.8, 59.4)^e^ | 57.4 (56.2, 58.6)^e^ | <0.0001 |
| Model 4 | 63.9 (63.8, 64.0) | 63.4 (63.4, 63.5) | 61.8 (61.5, 62.1) | 60.8 (60.5, 61.0) | 59.1 (58.8, 59.4) | 57.4 (56.1, 58.6) | <0.0001 |
| Arithmetic mean | 72.3 (72.3, 72.4) | 72.3 (72.2, 72.3) | 72.0 (71.8, 72.1) | 71.9 (71.8, 72.0) | 71.9 (71.7, 72.0) | 71.6 (71.0, 72.1) | <0.0001 |
| **Total protein, g/L** |  |  |  |  |  |  |  |
| Participants, n | 81,433 | 121,265 | 3,344 | 6,455 | 3,918 | 215 |  |
| Model 1 | 72.2 (72.2, 72.3) | 72.1 (72.1, 72.2) | 71.9 (71.7, 72.0) | 71.8 (71.7, 71.9) | 71.8 (71.7, 71.9) | 71.5 (71.0, 72.1) | <0.0001 |
| Model 2 | 72.2 (72.2, 72.2) | 72.1 (72.1, 72.2) | 71.9 (71.8, 72.0) | 71.9 (71.8, 72.0) | 71.8 (71.7, 71.9) | 71.6 (71.0, 72.1) | <0.0001 |
| Model 3 | 72.2 (72.2, 72.3)^a^ | 72.1 (72.1, 72.2)^b^ | 71.9 (71.7, 72.0)^c^ | 71.8 (71.7, 71.9)^c^ | 71.7 (71.6, 71.9)^c^ | 71.5 (70.9, 72.0)^abc^ | <0.0001 |
| Model 4 | 72.2 (72.2, 72.3) | 72.1 (72.1, 72.2) | 71.9 (71.7, 72.0) | 71.8 (71.7, 71.9) | 71.8 (71.6, 71.9) | 71.5 (71.0, 72.0) | <0.0001 |
| Arithmetic mean | 72.3 (72.3, 72.4) | 72.3 (72.2, 72.3) | 72.0 (71.8, 72.1) | 71.9 (71.8, 72.0) | 71.9 (71.7, 72.0) | 71.6 (71.0, 72.1) | <0.0001 |
| **Urea, mmol/L** |  |  |  |  |  |  |  |
| Participants, n | 89,725 | 133,253 | 3,698 | 7,149 | 4,348 | 229 |  |
| Model 1 | 5.23 (5.23, 5.24) | 5.08 (5.08, 5.09) | 4.76 (4.73, 4.80) | 4.57 (4.55, 4.60) | 4.36 (4.34, 4.39) | 3.98 (3.86, 4.10) | <0.0001 |
| Model 2 | 5.22 (5.21, 5.23) | 5.09 (5.08, 5.09) | 4.80 (4.76, 4.83) | 4.61 (4.58, 4.63) | 4.40 (4.37, 4.43) | 4.02 (3.90, 4.14) | <0.0001 |
| Model 3 | 5.23 (5.22, 5.23)^a^ | 5.08 (5.08, 5.09)^b^ | 4.79 (4.76, 4.83)^c^ | 4.60 (4.58, 4.62)^d^ | 4.39 (4.36, 4.42)^e^ | 4.01 (3.90, 4.13)^f^ | <0.0001 |
| Model 4 | 5.23 (5.22, 5.23) | 5.08 (5.08, 5.09) | 4.79 (4.75, 4.82) | 4.60 (4.57, 4.62) | 4.39 (4.36, 4.42) | 4.01 (3.89, 4.13) | <0.0001 |
| Arithmetic mean | 5.38 (5.37, 5.38) | 5.24 (5.23, 5.24) | 4.95 (4.91, 4.99) | 4.76 (4.73, 4.79) | 4.57 (4.53, 4.61) | 4.23 (4.07, 4.40) | <0.0001 |
| **Phosphate, mmol/L** |  |  |  |  |  |  |  |
| Participants, n | 81,388 | 121,121 | 3,340 | 6,451 | 3,916 | 215 |  |
| Model 1 | 1.18 (1.18, 1.18) | 1.18 (1.18, 1.19) | 1.19 (1.18, 1.19) | 1.19 (1.19, 1.20) | 1.20 (1.19, 1.20) | 1.17 (1.15, 1.19) | <0.0001 |
| Model 2 | 1.19 (1.18, 1.19) | 1.18 (1.18, 1.18) | 1.18 (1.18, 1.19) | 1.19 (1.18, 1.19) | 1.19 (1.19, 1.20) | 1.16 (1.15, 1.18) | 0.0005 |
| Model 3 | 1.19 (1.18, 1.19)^a^ | 1.18 (1.18, 1.18)^a^ | 1.18 (1.18, 1.19)^a^ | 1.19 (1.18, 1.19)^a^ | 1.19 (1.19, 1.20)^a^ | 1.17 (1.15, 1.19)^a^ | 0.0006 |
| Model 4 | 1.19 (1.18, 1.19) | 1.18 (1.18, 1.18) | 1.18 (1.18, 1.19) | 1.19 (1.18, 1.19) | 1.19 (1.19, 1.20) | 1.17 (1.15, 1.19) | 0.0004 |
| Arithmetic mean | 1.20 (1.19, 1.20) | 1.19 (1.19, 1.19) | 1.19 (1.19, 1.20) | 1.20 (1.19, 1.20) | 1.20 (1.20, 1.21) | 1.18 (1.16, 1.20) | 0.0001 |
| **Urate, µmol/L** |  |  |  |  |  |  |  |
| Participants, n | 89,682 | 133,157 | 3,699 | 7,146 | 4,342 | 229 |  |
| Model 1 | 269 (269, 270) | 261 (261, 261) | 247 (245, 249) | 244 (243, 246) | 241 (239, 242) | 260 (252, 268) | <0.0001 |
| Model 2 | 266 (265, 266) | 262 (262, 262) | 255 (254, 257) | 254 (252, 255) | 249 (247, 250) | 273 (266, 281) | <0.0001 |
| Model 3 | 265 (265, 266)^a^ | 262 (262, 263)^b^ | 256 (254, 258)^c^ | 254 (253, 255)^c^ | 250 (248, 251)^d^ | 276 (269, 284)^a^ | <0.0001 |
| Model 4 | 265 (265, 266) | 262 (262, 263) | 256 (255, 258) | 254 (253, 255) | 250 (248, 251) | 276 (269, 284) | <0.0001 |
| Arithmetic mean | 273 (273, 274) | 270 (270, 270) | 264 (262, 266) | 262 (260, 263) | 258 (256, 259) | 284 (276, 291) | <0.0001 |
| **Urinary creatinine, µmol/L** |  |  |  |  |  |  |  |
| Participants, n | 92,726 | 137,589 | 3,818 | 7,380 | 4,460 | 242 |  |
| Model 1 | 5,990 (5,970, 6,020) | 5,650 (5,630, 5,670) | 5,020 (4,920, 5,130) | 4,790 (4,710, 4,860) | 4,760 (4,670, 4,860) | 3,850 (3,540, 4,180) | <0.0001 |
| Model 2 | 5,880 (5,850, 5,900) | 5,690 (5,670, 5,710) | 5,280 (5,180, 5,390) | 5,060 (4,990, 5,140) | 5,000 (4,900, 5,090) | 4,120 (3,800, 4,470) | <0.0001 |
| Model 3 | 5,870 (5,850, 5,900)^a^ | 5,690 (5,670, 5,710)^b^ | 5,290 (5,180, 5,400)^c^ | 5,070 (4,990, 5,140)^d^ | 5,000 (4,910, 5,100)^d^ | 4,120 (3,800, 4,470)^e^ | <0.0001 |
| Model 4 | 5,870 (5,850, 5,900) | 5,690 (5,670, 5,710) | 5,300 (5,190, 5,410) | 5,080 (5,000, 5,150) | 5,010 (4,920, 5,110) | 4,130 (3,800, 4,480) | <0.0001 |
| Arithmetic mean | 7,240 (7,210, 7,280) | 7,080 (7,050, 7,100) | 6,750 (6,600, 6,900) | 6,430 (6,320, 6,540) | 6,440 (6,300, 6,580) | 5,480 (4,880, 6,080) | <0.0001 |
| **Urinary sodium/ creatinine ratio^4^** |  |  |  |  |  |  |  |
| Participants, n | 92,506 | 137,133 | 3,792 | 7,336 | 4,438 | 237 |  |
| Arithmetic mean, model 1 | 11.7 (11.7, 11.8) | 11.4 (11.3, 11.4) | 11.2 (11.0, 11.5) | 12.0 (11.8, 12.2) | 12.5 (12.2, 12.7) | 13.5 (12.5, 14.5) | <0.0001 |
| Arithmetic mean, model 2 | 11.7 (11.7, 11.8) | 11.4 (11.3, 11.4) | 11.2 (10.9, 11.4) | 11.9 (11.8, 12.1) | 12.4 (12.2, 12.6) | 13.4 (12.4, 14.4) | <0.0001 |
| Arithmetic mean, model 3 | 11.7 (11.7, 11.8)^a^ | 11.4 (11.3, 11.4)^b^ | 11.1 (10.9, 11.4)^b^ | 11.9 (11.7, 12.1)^ac^ | 12.4 (12.2, 12.6)^d^ | 13.4 (12.4, 14.4)^cd^ | <0.0001 |
| Arithmetic mean, model 4 | 11.7 (11.7, 11.8) | 11.4 (11.3, 11.4) | 11.1 (10.9, 11.4) | 11.9 (11.8, 12.1) | 12.4 (12.2, 12.6) | 13.4 (12.4, 14.4) | <0.0001 |
| **Urinary potassium/ creatinine ratio^4^** |  |  |  |  |  |  |  |
| Participants, n | 92,538 | 137,292 | 3,809 | 7,360 | 4,443 | 241 |  |
| Arithmetic mean, model 1 | 9.33 (9.30, 9.35) | 9.75 (9.73, 9.77) | 10.5 (10.3, 10.6) | 10.8 (10.7, 10.9) | 11.0 (10.9, 11.1) | 11.6 (11.0, 12.1) | <0.0001 |
| Arithmetic mean, model 2 | 9.42 (9.39, 9.45) | 9.71 (9.69, 9.74) | 10.2 (10.1, 10.3) | 10.5 (10.5, 10.6) | 10.8 (10.7, 10.9) | 11.2 (10.7, 11.7) | <0.0001 |
| Arithmetic mean, model 3 | 9.40 (9.37, 9.43)^a^ | 9.73 (9.70, 9.75)^b^ | 10.3 (10.1, 10.4)^c^ | 10.5 (10.4, 10.6)^d^ | 10.8 (10.7, 11.0)^e^ | 11.4 (10.8, 11.9)^e^ | <0.0001 |
| Arithmetic mean, model 4 | 9.40 (9.38, 9.43) | 9.72 (9.70, 9.75) | 10.3 (10.1, 10.4) | 10.5 (10.4, 10.6) | 10.8 (10.7, 10.9) | 11.3 (10.8, 11.9) | <0.0001 |

1. Unless otherwise specified, all estimates represent adjusted geometric means and 95% CIs, or if stated, adjusted arithmetic means and 95% CIs. Model 1 was adjusted for age (5 years categories) and fasting status (0-1, 2, 3, 4, 5, 6-7, ≥8 hours); model 2 was additionally adjusted for BMI (<20, 20.0-22.4, 22.5-24.9, 25.0-27.4, 27.5-29.9, 30.0-32.4, 32.5-34.9, ≥35.0 kg/m^2^, unknown), model 3 was additionally adjusted for alcohol consumption (<1, 1-7, 8-15, ≥16 g/d, unknown) and smoking status (never, previous, current <15 cigarettes/day, current ≥15 cigarettes/day, unknown), model 4 was additionally adjusted for physical activity (low <10 excess MET hours/week, moderate 10-49 excess MET hours/week, high ≥50 excess MET hours/week, unknown). Arithmetic means were based on model 3 unless otherwise specified.
2. Includes participants who consume any red or processed meat, regardless of whether they consume poultry, fish, or dairy. Cut-offs of regular and low consumption determined based on consumption of red and processed meat (beef, lamb, pork, processed meat) as reported on the touchscreen questionnaire.
3. Represents p for heterogeneity across the six diet groups based on Wald tests. Groups that do not share a superscript letter (ordered alphabetically from left to right) were significantly different at the 5% level from post hoc Bonferroni-corrected pairwise comparisons after linear regression (model 3).
4. Expressed as per mmol/L of urinary sodium or potassium to per mmol/L of urinary creatinine.

**Supplemental Table 22**: **Renal-related** serum and urinary biomarker concentrations (numbers, adjusted means and 95% CIs)^1^ in **white British men** by diet group in the UK Biobank.

| **Renal-related serum or urinary biomarker** | **Regular meat eaters (>3 times/week)^2^** | **Low meat eaters (>3 times/week)^2^** | **Poultry eaters** | **Fish eaters** | **Vegetarians** | **Vegans** | **p-het^3^** |
| --- | --- | --- | --- | --- | --- | --- | --- |
| **Cystatin C, mg/L** |  |  |  |  |  |  |  |
| Participants, n | 118,945 | 75,943 | 1,063 | 2,762 | 2,096 | 169 |  |
| Model 1 | 0.93 (0.93, 0.93) | 0.93 (0.92, 0.93) | 0.92 (0.91, 0.93) | 0.92 (0.92, 0.93) | 0.95 (0.95, 0.96) | 0.94 (0.92, 0.97) | <0.0001 |
| Model 2 | 0.93 (0.93, 0.93) | 0.93 (0.93, 0.93) | 0.93 (0.92, 0.94) | 0.94 (0.93, 0.94) | 0.97 (0.96, 0.97) | 0.97 (0.94, 0.99) | <0.0001 |
| Model 3 | 0.93 (0.93, 0.93)^a^ | 0.93 (0.93, 0.93)^b^ | 0.92 (0.92, 0.93)^ab^ | 0.94 (0.93, 0.94)^a^ | 0.96 (0.96, 0.97)^c^ | 0.95 (0.93, 0.97)^abc^ | <0.0001 |
| Model 4 | 0.93 (0.93, 0.93) | 0.93 (0.93, 0.93) | 0.93 (0.92, 0.93) | 0.94 (0.93, 0.94) | 0.96 (0.96, 0.97) | 0.95 (0.93, 0.97) | <0.0001 |
| Arithmetic mean | 0.94 (0.94, 0.95) | 0.94 (0.94, 0.94) | 0.94 (0.93, 0.95) | 0.95 (0.94, 0.95) | 0.97 (0.97, 0.98) | 0.96 (0.94, 0.99) | <0.0001 |
| **Serum creatinine, µmol/L** |  |  |  |  |  |  |  |
| Participants, n | 118,880 | 75,920 | 1,063 | 2,759 | 2,096 | 169 |  |
| Model 1 | 80.9 (80.9, 81.0) | 80.1 (80.0, 80.2) | 76.9 (76.1, 77.6) | 73.5 (73.0, 73.9) | 70.9 (70.4, 71.4) | 68.6 (66.9, 70.3) | <0.0001 |
| Model 2 | 80.9 (80.8, 80.9) | 80.1 (80.0, 80.2) | 77.5 (76.7, 78.3) | 74.1 (73.7, 74.6) | 71.5 (71.0, 72.0) | 69.7 (68.0, 71.5) | <0.0001 |
| Model 3 | 81.0 (80.9, 81.1)^a^ | 79.9 (79.8, 80.0)^b^ | 77.0 (76.2, 77.7)^c^ | 73.8 (73.4, 74.3)^d^ | 71.2 (70.7, 71.7)^e^ | 69.2 (67.6, 71.0)^e^ | <0.0001 |
| Model 4 | 81.0 (80.9, 81.1) | 79.9 (79.8, 80.0) | 77.0 (76.3, 77.8) | 73.8 (73.4, 74.3) | 71.2 (70.7, 71.7) | 69.2 (67.6, 71.0) | <0.0001 |
| Arithmetic mean | 72.5 (72.5, 72.5) | 72.5 (72.5, 72.5) | 72.4 (72.2, 72.7) | 72.2 (72.0, 72.3) | 72.1 (71.9, 72.3) | 71.3 (70.7, 72.0) | <0.0001 |
| **Total protein, g/L** |  |  |  |  |  |  |  |
| Participants, n | 109,787 | 70,030 | 974 | 2,542 | 1,934 | 153 |  |
| Model 1 | 72.4 (72.4, 72.4) | 72.4 (72.3, 72.4) | 72.2 (71.9, 72.4) | 72.0 (71.8, 72.1) | 71.9 (71.7, 72.1) | 71.1 (70.4, 71.7) | <0.0001 |
| Model 2 | 72.4 (72.4, 72.4) | 72.4 (72.3, 72.4) | 72.3 (72.0, 72.5) | 72.1 (71.9, 72.2) | 72.0 (71.8, 72.2) | 71.2 (70.6, 71.8) | <0.0001 |
| Model 3 | 72.4 (72.4, 72.4)^a^ | 72.4 (72.3, 72.4)^a^ | 72.3 (72.1, 72.5)^ab^ | 72.0 (71.9, 72.2)^bc^ | 72.0 (71.8, 72.2)^bc^ | 71.2 (70.6, 71.8)^c^ | <0.0001 |
| Model 4 | 72.4 (72.4, 72.4) | 72.4 (72.3, 72.4) | 72.3 (72.1, 72.6) | 72.1 (71.9, 72.2) | 72.0 (71.8, 72.2) | 71.2 (70.6, 71.9) | <0.0001 |
| Arithmetic mean | 72.5 (72.5, 72.5) | 72.5 (72.5, 72.5) | 72.4 (72.2, 72.7) | 72.2 (72.0, 72.3) | 72.1 (71.9, 72.3) | 71.3 (70.7, 72.0) | <0.0001 |
| **Urea, mmol/L** |  |  |  |  |  |  |  |
| Participants, n | 118,858 | 75,907 | 1,063 | 2,757 | 2,097 | 169 |  |
| Model 1 | 5.52 (5.52, 5.53) | 5.43 (5.43, 5.44) | 5.18 (5.11, 5.25) | 4.90 (4.86, 4.95) | 4.67 (4.62, 4.72) | 4.44 (4.29, 4.60) | <0.0001 |
| Model 2 | 5.52 (5.51, 5.53) | 5.44 (5.43, 5.45) | 5.22 (5.15, 5.29) | 4.94 (4.90, 4.99) | 4.71 (4.66, 4.76) | 4.51 (4.36, 4.67) | <0.0001 |
| Model 3 | 5.53 (5.53, 5.54)^a^ | 5.42 (5.41, 5.43)^b^ | 5.17 (5.10, 5.24)^c^ | 4.91 (4.87, 4.95)^d^ | 4.68 (4.63, 4.72)^e^ | 4.47 (4.31, 4.63)^e^ | <0.0001 |
| Model 4 | 5.53 (5.53, 5.54) | 5.42 (5.41, 5.43) | 5.15 (5.08, 5.23) | 4.91 (4.86, 4.95) | 4.68 (4.63, 4.72) | 4.46 (4.31, 4.62) | <0.0001 |
| Arithmetic mean | 5.70 (5.69, 5.71) | 5.58 (5.57, 5.59) | 5.34 (5.26, 5.43) | 5.06 (5.01, 5.12) | 4.84 (4.78, 4.90) | 4.66 (4.45, 4.87) | <0.0001 |
| **Phosphate, mmol/L** |  |  |  |  |  |  |  |
| Participants, n | 109,727 | 69,982 | 972 | 2,538 | 1,933 | 153 |  |
| Model 1 | 1.11 (1.11, 1.11) | 1.10 (1.10, 1.10) | 1.09 (1.08, 1.10) | 1.10 (1.09, 1.11) | 1.10 (1.10, 1.11) | 1.07 (1.04, 1.09) | <0.0001 |
| Model 2 | 1.11 (1.11, 1.11) | 1.10 (1.10, 1.10) | 1.09 (1.08, 1.10) | 1.10 (1.09, 1.10) | 1.10 (1.09, 1.11) | 1.06 (1.03, 1.08) | <0.0001 |
| Model 3 | 1.11 (1.11, 1.11)^a^ | 1.10 (1.10, 1.10)^a^ | 1.09 (1.08, 1.10)^ab^ | 1.10 (1.09, 1.10)^ab^ | 1.10 (1.09, 1.11)^ab^ | 1.06 (1.04, 1.09)^b^ | <0.0001 |
| Model 4 | 1.11 (1.11, 1.11) | 1.10 (1.10, 1.10) | 1.09 (1.08, 1.10) | 1.10 (1.09, 1.10) | 1.10 (1.09, 1.11) | 1.06 (1.04, 1.09) | <0.0001 |
| Arithmetic mean | 1.12 (1.12, 1.12) | 1.12 (1.11, 1.12) | 1.10 (1.09, 1.11) | 1.11 (1.10, 1.12) | 1.12 (1.11, 1.12) | 1.08 (1.05, 1.10) | <0.0001 |
| **Urate, µmol/L** |  |  |  |  |  |  |  |
| Participants, n | 118,822 | 75,869 | 1,061 | 2,758 | 2,093 | 169 |  |
| Model 1 | 351 (350, 351) | 344 (344, 345) | 329 (325, 333) | 327 (325, 329) | 317 (314, 320) | 331 (321, 341) | <0.0001 |
| Model 2 | 349 (349, 350) | 346 (345, 346) | 340 (336, 344) | 338 (336, 341) | 328 (325, 331) | 349 (340, 360) | <0.0001 |
| Model 3 | 349 (348, 349)^a^ | 346 (346, 347)^b^ | 344 (340, 348)^abc^ | 340 (337, 342)^c^ | 330 (327, 333)^d^ | 355 (345, 366)^ab^ | <0.0001 |
| Model 4 | 349 (348, 349) | 346 (346, 347) | 344 (340, 348) | 340 (337, 342) | 330 (327, 332) | 355 (345, 366) | <0.0001 |
| Arithmetic mean | 356 (356, 356) | 354 (353, 354) | 352 (348, 356) | 346 (344, 349) | 338 (335, 340) | 361 (351, 371) | <0.0001 |
| **Urinary creatinine, µmol/L** |  |  |  |  |  |  |  |
| Participants, n | 122,472 | 78,330 | 1,087 | 2,831 | 2,145 | 170 |  |
| Model 1 | 9,460 (9,430, 9,490) | 8,920 (8,880, 8,950) | 8,130 (7,850, 8,420) | 7,740 (7,570, 7,910) | 7,510 (7,330, 7,700) | 6,980 (6,380, 7,630) | <0.0001 |
| Model 2 | 9,390 (9,360, 9,420) | 8,990 (8,950, 9,020) | 8,570 (8,280, 8,870) | 8,180 (8,010, 8,360) | 7,940 (7,750, 8,140) | 7,610 (6,970, 8,300) | <0.0001 |
| Model 3 | 9,390 (9,360, 9,420)^a^ | 8,980 (8,940, 9,020)^b^ | 8,560 (8,260, 8,860)^bc^ | 8,180 (8,010, 8,370)^cd^ | 7,940 (7,750, 8,140)^d^ | 7,640(7,000, 8,340)^cd^ | <0.0001 |
| Model 4 | 9,390 (9,360, 9,420) | 8,980 (8,950, 9,020) | 8,570 (8,280, 8,880) | 8,200 (8,030, 8,380) | 7,940 (7,750, 8,140) | 7,660 (7,010, 8,360) | <0.0001 |
| Arithmetic mean | 11,000 (11,000,11,000) | 10,600 (10,600,10,700) | 10,400 (10,000,10,700) | 9,930 (9,710,10,100) | 9,770 (9,520,10,000) | 9,700 (8,800,10,600) | <0.0001 |
| **Urinary sodium/ creatinine ratio^4^** |  |  |  |  |  |  |  |
| Participants, n | 122,366 | 78,231 | 1,081 | 2,826 | 2,141 | 169 |  |
| Arithmetic mean, model 1 | 9.74 (9.71, 9.77) | 9.38 (9.34, 9.43) | 9.03 (8.67, 9.38) | 9.88 (9.66, 10.10) | 10.7 (10.4, 10.9) | 10.0 (9.12, 10.9) | <0.0001 |
| Arithmetic mean, model 2 | 9.74 (9.70, 9.77) | 9.39 (9.35, 9.43) | 9.01 (8.65, 9.37) | 9.87 (9.65, 10.09) | 10.6 (10.4, 10.9) | 9.98 (9.07, 10.9) | <0.0001 |
| Arithmetic mean, model 3 | 9.73 (9.70, 9.76)^a^ | 9.40 (9.36, 9.44)^b^ | 9.00 (8.64, 9.36)^b^ | 9.87 (9.64, 10.09)^a^ | 10.6 (10.4, 10.9)^c^ | 9.88 (8.97, 10.8)^abc^ | <0.0001 |
| Arithmetic mean, model 4 | 9.73 (9.70, 9.76) | 9.41 (9.36, 9.45) | 8.95 (8.59, 9.31) | 9.86 (9.64, 10.08) | 10.6 (10.4, 10.9) | 9.92 (9.01, 10.8) | <0.0001 |
| **Urinary potassium/ creatinine ratio^4^** |  |  |  |  |  |  |  |
| Participants, n | 122,213 | 78,146 | 1,083 | 2,821 | 2,139 | 170 |  |
| Arithmetic mean, model 1 | 6.90 (6.88, 6.92) | 7.23 (7.21, 7.26) | 7.86 (7.63, 8.09) | 8.29 (8.15, 8.44) | 8.32 (8.16, 8.49) | 8.64 (8.05, 9.23) | <0.0001 |
| Arithmetic mean, model 2 | 6.93 (6.91, 6.96) | 7.20 (7.17, 7.23) | 7.66 (7.43, 7.90) | 8.08 (7.94, 8.23) | 8.12 (7.96, 8.29) | 8.34 (7.75, 8.92) | <0.0001 |
| Arithmetic mean, model 3 | 6.91 (6.89, 6.93)^a^ | 7.23 (7.20, 7.26)^b^ | 7.80 (7.57, 8.03)^c^ | 8.12 (7.98, 8.27)^c^ | 8.20 (8.04, 8.37)^c^ | 8.52 (7.94, 9.11)^c^ | <0.0001 |
| Arithmetic mean, model 4 | 6.91 (6.89, 6.93) | 7.23 (7.20, 7.26) | 7.78 (7.55, 8.01) | 8.10 (7.96, 8.25) | 8.20 (8.03, 8.36) | 8.49 (7.90, 9.07) | <0.0001 |

1. Unless otherwise specified, all estimates represent adjusted geometric means and 95% CIs, or if stated, adjusted arithmetic means and 95% CIs. Model 1 was adjusted for age (5 years categories) and fasting status (0-1, 2, 3, 4, 5, 6-7, ≥8 hours); model 2 was additionally adjusted for BMI (<20, 20.0-22.4, 22.5-24.9, 25.0-27.4, 27.5-29.9, 30.0-32.4, 32.5-34.9, ≥35.0 kg/m^2^, unknown), model 3 was additionally adjusted for alcohol consumption (<1, 1-7, 8-15, ≥16 g/d, unknown) and smoking status (never, previous, current <15 cigarettes/day, current ≥15 cigarettes/day, unknown), model 4 was additionally adjusted for physical activity (low <10 excess MET hours/week, moderate 10-49 excess MET hours/week, high ≥50 excess MET hours/week, unknown). Arithmetic means were based on model 3 unless otherwise specified.
2. Includes participants who consume any red or processed meat, regardless of whether they consume poultry, fish, or dairy. Cut-offs of regular and low consumption determined based on consumption of red and processed meat (beef, lamb, pork, processed meat) as reported on the touchscreen questionnaire.
3. Represents p for heterogeneity across the six diet groups based on Wald tests. ﻿ Groups that do not share a superscript letter (ordered alphabetically from left to right) were significantly different at the 5% level from post hoc Bonferroni-corrected pairwise comparisons after linear regression (model 3).
4. Expressed as per mmol/L of urinary sodium or potassium to per mmol/L of urinary creatinine.

**Supplemental Table 23**: **Renal-related** serum and urinary biomarker concentrations (numbers, adjusted means and 95% CIs)^1^ in **British Indian participants** by diet group in the UK Biobank.

| **Renal-related serum or urinary biomarker** | **All participants** | | |  | **Women** | | |  | **Men** | | |
| --- | --- | --- | --- | --- | --- | --- | --- | --- | --- | --- | --- |
|  | **Meat-eaters** | **Vegetarians** | **p-het^2^** |  | **Meat-eaters** | **Vegetarians** | **p-het^2^** |  | **Meat-eaters** | **Vegetarians** | **p-het^2^** |
| **Cystatin C, mg/L** |  |  |  |  |  |  |  |  |  |  |  |
| Participants, n | 3,823 | 1,343 |  |  | 1,603 | 869 |  |  | 2,220 | 474 |  |
| Model 1 | 0.93 (0.93, 0.94) | 0.98 (0.97, 0.99) | <0.0001 |  | 0.89 (0.88, 0.90) | 0.94 (0.93, 0.95) | <0.0001 |  | 0.97 (0.96, 0.98) | 1.02 (1.00, 1.04) | <0.0001 |
| Model 2 | 0.93 (0.92, 0.93) | 0.99 (0.98, 0.99) | <0.0001 |  | 0.89 (0.88, 0.90) | 0.94 (0.93, 0.95) | <0.0001 |  | 0.97 (0.96, 0.98) | 1.02 (1.01, 1.04) | <0.0001 |
| Model 3 | 0.93 (0.93, 0.94) | 0.98 (0.97, 0.99) | <0.0001 |  | 0.89 (0.89, 0.90) | 0.94 (0.93, 0.95) | <0.0001 |  | 0.97 (0.97, 0.98) | 1.01 (0.99, 1.02) | 0.001 |
| Model 4 | 0.93 (0.93, 0.94) | 0.98 (0.97, 0.98) | <0.0001 |  | 0.89 (0.89, 0.90) | 0.94 (0.93, 0.95) | <0.0001 |  | 0.97 (0.97, 0.98) | 1.00 (0.99, 1.02) | 0.001 |
| Arithmetic mean | 0.96 (0.95, 0.96) | 0.99 (0.98, 1.01) | <0.0001 |  | 0.91 (0.90, 0.92) | 0.96 (0.94, 0.97) | <0.0001 |  | 1.00 (0.99, 1.01) | 1.01 (0.99, 1.04) | 0.41 |
| **Serum creatinine, µmol/L** |  |  |  |  |  |  |  |  |  |  |  |
| Participants, n | 3,821 | 1,343 |  |  | 1,603 | 869 |  |  | 2,218 | 474 |  |
| Model 1 | 70.7 (70.2, 71.1) | 66.5 (65.8, 67.2) | <0.0001 |  | 61.1 (60.6, 61.6) | 58.6 (57.9, 59.3) | <0.0001 |  | 80.6 (79.9, 81.3) | 73.8 (72.5, 75.2) | <0.0001 |
| Model 2 | 70.6 (70.2, 71.1) | 66.5 (65.8, 67.2) | <0.0001 |  | 61.1 (60.6, 61.7) | 58.6 (57.9, 59.2) | <0.0001 |  | 80.6 (79.9, 81.3) | 73.9 (72.5, 75.3) | <0.0001 |
| Model 3 | 70.6 (70.2, 71.0) | 66.7 (65.9, 67.4) | <0.0001 |  | 61.0 (60.4, 61.5) | 58.9 (58.2, 59.6) | <0.0001 |  | 80.7 (80.0, 81.4) | 73.6 (72.1, 75.1) | <0.0001 |
| Model 4 | 70.6 (70.2, 71.0) | 66.6 (65.9, 67.4) | <0.0001 |  | 61.0 (60.5, 61.5) | 58.9 (58.2, 59.6) | <0.0001 |  | 80.7 (80.0, 81.4) | 73.6 (72.1, 75.0) | <0.0001 |
| Arithmetic mean | 75.1 (74.9, 75.2) | 74.4 (74.2, 74.7) | <0.0001 |  | 75.3 (75.0, 75.5) | 74.5 (74.2, 74.8) | 0.0003 |  | 74.9 (74.7, 75.1) | 74.4 (73.9, 74.8) | 0.03 |
| **Total protein, g/L** |  |  |  |  |  |  |  |  |  |  |  |
| Participants, n | 3,493 | 1,204 |  |  | 1,448 | 776 |  |  | 2,045 | 428 |  |
| Model 1 | 74.9 (74.7, 75.0) | 74.4 (74.2, 74.7) | 0.003 |  | 75.0 (74.8, 75.3) | 74.6 (74.3, 74.9) | 0.02 |  | 74.8 (74.6, 74.9) | 74.3 (73.9, 74.7) | 0.04 |
| Model 2 | 74.9 (74.7, 75.0) | 74.4 (74.2, 74.7) | 0.002 |  | 75.0 (74.8, 75.3) | 74.6 (74.2, 74.9) | 0.01 |  | 74.8 (74.6, 74.9) | 74.3 (73.9, 74.7) | 0.07 |
| Model 3 | 74.9 (74.8, 75.1) | 74.3 (74.0, 74.6) | <0.0001 |  | 75.1 (74.9, 75.4) | 74.4 (74.1, 74.7) | 0.0003 |  | 74.8 (74.6, 75.0) | 74.3 (73.8, 74.7) | 0.04 |
| Model 4 | 74.9 (74.8, 75.1) | 74.3 (74.0, 74.6) | <0.0001 |  | 75.1 (74.9, 75.4) | 74.4 (74.1, 74.7) | 0.0003 |  | 74.8 (74.6, 75.0) | 74.3 (73.8, 74.7) | 0.04 |
| Arithmetic mean | 75.1 (74.9, 75.2) | 74.4 (74.2, 74.7) | <0.0001 |  | 75.3 (75.0, 75.5) | 74.5 (74.2, 74.8) | 0.0003 |  | 74.9 (74.7, 75.1) | 74.4 (73.9, 74.8) | 0.03 |
| **Urea, mmol/L** |  |  |  |  |  |  |  |  |  |  |  |
| Participants, n | 3,821 | 1,343 |  |  | 1,603 | 869 |  |  | 2,218 | 474 |  |
| Model 1 | 4.91 (4.87, 4.95) | 4.32 (4.26, 4.38) | <0.0001 |  | 4.66 (4.60, 4.71) | 4.13 (4.06, 4.20) | <0.0001 |  | 5.16 (5.10, 5.21) | 4.47 (4.36, 4.57) | <0.0001 |
| Model 2 | 4.91 (4.87, 4.95) | 4.32 (4.26, 4.38) | <0.0001 |  | 4.66 (4.60, 4.71) | 4.13 (4.06, 4.20) | <0.0001 |  | 5.16 (5.10, 5.21) | 4.47 (4.36, 4.57) | <0.0001 |
| Model 3 | 4.91 (4.87, 4.95) | 4.31 (4.25, 4.38) | <0.0001 |  | 4.65 (4.59, 4.71) | 4.14 (4.07, 4.21) | <0.0001 |  | 5.16 (5.11, 5.22) | 4.44 (4.33, 4.55) | <0.0001 |
| Model 4 | 4.91 (4.87, 4.95) | 4.31 (4.25, 4.38) | <0.0001 |  | 4.65 (4.60, 4.71) | 4.14 (4.07, 4.21) | <0.0001 |  | 5.16 (5.10, 5.22) | 4.45 (4.34, 4.56) | <0.0001 |
| Arithmetic mean | 5.10 (5.05, 5.14) | 4.47 (4.39, 4.55) | <0.0001 |  | 4.80 (4.75, 4.86) | 4.28 (4.20, 4.36) | <0.0001 |  | 5.36 (5.30, 5.43) | 4.56 (4.42, 4.71) | <0.0001 |
| **Phosphate, mmol/L** |  |  |  |  |  |  |  |  |  |  |  |
| Participants, n | 3,486 | 1,205 |  |  | 1,444 | 776 |  |  | 2,042 | 429 |  |
| Model 1 | 1.19 (1.18, 1.19) | 1.19 (1.18, 1.20) | 0.12 |  | 1.22 (1.21, 1.23) | 1.23 (1.21, 1.24) | 0.48 |  | 1.16 (1.15, 1.16) | 1.17 (1.15, 1.18) | 0.21 |
| Model 2 | 1.19 (1.18, 1.19) | 1.19 (1.18, 1.20) | 0.12 |  | 1.22 (1.21, 1.23) | 1.23 (1.21, 1.24) | 0.53 |  | 1.16 (1.15, 1.16) | 1.17 (1.15, 1.18) | 0.18 |
| Model 3 | 1.19 (1.18, 1.19) | 1.19 (1.18, 1.20) | 0.17 |  | 1.22 (1.21, 1.23) | 1.23 (1.21, 1.24) | 0.46 |  | 1.16 (1.15, 1.16) | 1.17 (1.15, 1.18) | 0.24 |
| Model 4 | 1.19 (1.18, 1.19) | 1.19 (1.18, 1.20) | 0.17 |  | 1.22 (1.21, 1.23) | 1.23 (1.21, 1.24) | 0.50 |  | 1.16 (1.15, 1.16) | 1.17 (1.15, 1.18) | 0.19 |
| Arithmetic mean | 1.20 (1.19, 1.20) | 1.21 (1.20, 1.22) | 0.16 |  | 1.23 (1.22, 1.24) | 1.24 (1.23, 1.25) | 0.39 |  | 1.17 (1.16, 1.17) | 1.18 (1.16, 1.19) | 0.28 |
| **Urate, µmol/L** |  |  |  |  |  |  |  |  |  |  |  |
| Participants, n | 3,819 | 1,342 |  |  | 1,604 | 868 |  |  | 2,215 | 474 |  |
| Model 1 | 312 (310, 314) | 297 (293, 301) | <0.0001 |  | 274 (271, 277) | 264 (260, 269) | 0.0004 |  | 351 (348, 354) | 327 (321, 333) | <0.0001 |
| Model 2 | 311 (309, 314) | 298 (294, 301) | <0.0001 |  | 274 (271, 277) | 265 (261, 269) | 0.0004 |  | 351 (348, 354) | 328 (322, 334) | <0.0001 |
| Model 3 | 311 (308, 313) | 300 (297, 304) | <0.0001 |  | 273 (270, 276) | 266 (262, 270) | 0.01 |  | 349 (346, 352) | 334 (328, 341) | <0.0001 |
| Model 4 | 311 (308, 313) | 300 (297, 304) | <0.0001 |  | 273 (270, 276) | 266 (262, 270) | 0.01 |  | 349 (346, 352) | 334 (328, 341) | <0.0001 |
| Arithmetic mean | 321 (319, 323) | 310 (306, 314) | <0.0001 |  | 282 (279, 285) | 274 (270, 278) | 0.004 |  | 357 (354, 360) | 341 (334, 348) | <0.0001 |
| **Urinary creatinine, µmol/L** |  |  |  |  |  |  |  |  |  |  |  |
| Participants, n | 3,921 | 1,379 |  |  | 1,645 | 896 |  |  | 2,276 | 483 |  |
| Model 1 | 6,900 (6,760, 7,050) | 5,980 (5,770, 6,190) | <0.0001 |  | 5,450 (5,280, 5,640) | 4,720 (4,510, 4,930) | <0.0001 |  | 8,580 (8,350, 8,810) | 7,420 (7,010, 7,860) | <0.0001 |
| Model 2 | 6,900 (6,760, 7,040) | 5,990 (5,780, 6,210) | <0.0001 |  | 5,460 (5,280, 5,640) | 4,710 (4,510, 4,930) | <0.0001 |  | 8,560 (8,340, 8,780) | 7,510 (7,090, 7,940) | <0.0001 |
| Model 3 | 6,880 (6,740, 7,030) | 6,030 (5,810, 6,250) | <0.0001 |  | 5,430 (5,260, 5,620) | 4,750 (4,540, 4,970) | <0.0001 |  | 8,550 (8,320, 8,770) | 7,550 (7,110, 8,020) | 0.0003 |
| Model 4 | 6,890 (6,740, 7,030) | 6,020 (5,800, 6,240) | <0.0001 |  | 5,440 (5,260, 5,620) | 4,740 (4,530, 4,970) | <0.0001 |  | 8,550 (8,330, 8,780) | 7,540 (7,100, 8,010) | 0.0002 |
| Arithmetic mean | 8,660 (8,490, 8,840) | 7,780 (7,470, 8,090) | <0.0001 |  | 6,830 (6,600, 7,060) | 6,100 (5,770, 6,420) | 0.0004 |  | 10,300 (10,100, 10,600) | 9,290 (8,720, 9,870) | 0.002 |
| **Urinary sodium/ creatinine ratio^3^** |  |  |  |  |  |  |  |  |  |  |  |
| Participants, n | 3,914 | 1,376 |  |  | 1,644 | 893 |  |  | 2,270 | 483 |  |
| Arithmetic mean, model 1 | 12.8 (12.6, 13.1) | 15.7 (15.2, 16.1) | <0.0001 |  | 14.6 (14.2, 15.0) | 17.7 (17.1, 18.2) | <0.0001 |  | 11.2 (10.9, 11.5) | 13.7 (13.1, 14.3) | <0.0001 |
| Arithmetic mean, model 2 | 12.8 (12.6, 13.1) | 15.6 (15.2, 16.1) | <0.0001 |  | 14.6 (14.2, 15.0) | 17.7 (17.1, 18.2) | <0.0001 |  | 11.2 (10.9, 11.5) | 13.6 (13.0, 14.2) | <0.0001 |
| Arithmetic mean, model 3 | 12.9 (12.7, 13.2) | 15.4 (15.0, 15.9) | <0.0001 |  | 14.8 (14.3, 15.2) | 17.3 (16.7, 17.9) | <0.0001 |  | 11.2 (11.0, 11.5) | 13.5 (12.8, 14.1) | <0.0001 |
| Arithmetic mean, model 4 | 12.9 (12.7, 13.2) | 15.4 (15.0, 15.9) | <0.0001 |  | 14.8 (14.3, 15.2) | 17.3 (16.8, 17.9) | <0.0001 |  | 11.2 (11.0, 11.5) | 13.5 (12.9, 14.1) | <0.0001 |
| **Urinary potassium/ creatinine ratio^3^** |  |  |  |  |  |  |  |  |  |  |  |
| Participants, n | 3,916 | 1,376 |  |  | 1,644 | 895 |  |  | 2,272 | 481 |  |
| Arithmetic mean, model 1 | 7.47 (7.37, 7.57) | 8.44 (8.27, 8.61) | <0.0001 |  | 8.58 (8.40, 8.75) | 9.58 (9.34, 9.81) | <0.0001 |  | 6.45 (6.34, 6.56) | 7.37 (7.14, 7.60) | <0.0001 |
| Arithmetic mean, model 2 | 7.47 (7.38, 7.57) | 8.43 (8.26, 8.59) | <0.0001 |  | 8.58 (8.41, 8.75) | 9.57 (9.34, 9.80) | <0.0001 |  | 6.46 (6.35, 6.56) | 7.34 (7.11, 7.56) | <0.0001 |
| Arithmetic mean, model 3 | 7.42 (7.32, 7.52) | 8.58 (8.41, 8.76) | <0.0001 |  | 8.53 (8.36, 8.70) | 9.66 (9.42, 9.90) | <0.0001 |  | 6.41 (6.30, 6.51) | 7.58 (7.33, 7.82) | <0.0001 |
| Arithmetic mean, model 4 | 7.42 (7.32, 7.52) | 8.59 (8.42, 8.76) | <0.0001 |  | 8.53 (8.35, 8.70) | 9.67 (9.43, 9.91) | <0.0001 |  | 6.41 (6.30, 6.51) | 7.58 (7.33, 7.82) | <0.0001 |

1. Unless otherwise specified, all estimates represent adjusted geometric means and 95% CIs, or if stated, adjusted arithmetic means and 95% CIs. Model 1 was adjusted for sex (for analyses with all participants), age (5 years categories) and fasting status (0-1, 2, 3, 4, 5, 6-7, ≥8 hours); model 2 was additionally adjusted for BMI (<20, 20.0-22.4, 22.5-24.9, 25.0-27.4, 27.5-29.9, 30.0-32.4, 32.5-34.9, ≥35.0 kg/m^2^, unknown), model 3 was additionally adjusted for alcohol consumption (<1, 1-7, 8-15, ≥16 g/d, unknown) and smoking status (never, previous, current <15 cigarettes/day, current ≥15 cigarettes/day, unknown), model 4 was additionally adjusted for physical activity (low <10 excess MET hours/week, moderate 10-49 excess MET hours/week, high ≥50 excess MET hours/week, unknown). Arithmetic means were based on model 3 unless otherwise specified.
2. Represents p for heterogeneity across the two diet groups based on Wald tests.
3. Expressed as per mmol/L of urinary sodium or potassium to per mmol/L of urinary creatinine.

**Supplemental Table 24**: **Liver-related** serum biomarker concentrations (numbers, adjusted means and 95% CIs)^1^ in **white British participants** by diet group in the UK Biobank.

| **Liver-related serum biomarker** | **Regular meat eaters (>3 times/week)^2^** | **Low meat eaters (>3 times/week)^2^** | **Poultry eaters** | **Fish eaters** | **Vegetarians** | **Vegans** | **p-het^3^** |
| --- | --- | --- | --- | --- | --- | --- | --- |
| **Albumin, g/L** |  |  |  |  |  |  |  |
| Participants, n | 191,481 | 191,475 | 4,321 | 9,011 | 5,856 | 369 |  |
| Model 1 | 45.1 (45.1, 45.1) | 45.2 (45.2, 45.2) | 45.2 (45.1, 45.3) | 45.2 (45.2, 45.3) | 45.0 (44.9, 45.1) | 45.0 (44.7, 45.2) | <0.0001 |
| Model 2 | 45.1 (45.1, 45.2) | 45.2 (45.2, 45.2) | 45.1 (45.0, 45.2) | 45.1 (45.1, 45.2) | 44.9 (44.8, 44.9) | 44.8 (44.5, 45.0) | <0.0001 |
| Model 3 | 45.1 (45.1, 45.2)^a^ | 45.2 (45.2, 45.2)^a^ | 45.1 (45.1, 45.2)^a^ | 45.1 (45.1, 45.2)^a^ | 44.9 (44.8, 45.0)^b^ | 44.9 (44.6, 45.1)^ab^ | <0.0001 |
| Model 4 | 45.1 (45.1, 45.2) | 45.2 (45.2, 45.2) | 45.1 (45.1, 45.2) | 45.1 (45.1, 45.2) | 44.9 (44.8, 45.0) | 44.9 (44.6, 45.1) | <0.0001 |
| Arithmetic mean | 45.2 (45.2, 45.2) | 45.3 (45.2, 45.3) | 45.2 (45.1, 45.3) | 45.2 (45.1, 45.2) | 45.0 (44.9, 45.1) | 44.9 (44.7, 45.2) | <0.0001 |
| **Direct bilirubin, µmol/L** |  |  |  |  |  |  |  |
| Participants, n | 179,452 | 176,307 | 3,964 | 8,396 | 5,337 | 351 |  |
| Model 1 | 1.69 (1.69, 1.69) | 1.72 (1.72, 1.72) | 1.74 (1.72, 1.76) | 1.77 (1.76, 1.79) | 1.71 (1.70, 1.73) | 1.79 (1.72, 1.85) | <0.0001 |
| Model 2 | 1.69 (1.69, 1.70) | 1.72 (1.71, 1.72) | 1.72 (1.70, 1.74) | 1.75 (1.74, 1.76) | 1.69 (1.68, 1.71) | 1.75 (1.69, 1.82) | <0.0001 |
| Model 3 | 1.70 (1.69, 1.70)^a^ | 1.72 (1.71, 1.72)^b^ | 1.72 (1.71, 1.74)^bcd^ | 1.75 (1.74, 1.76)^c^ | 1.69 (1.68, 1.71)^ad^ | 1.76 (1.70, 1.82)^abcd^ | <0.0001 |
| Model 4 | 1.70 (1.69, 1.70) | 1.72 (1.71, 1.72) | 1.72 (1.71, 1.74) | 1.75 (1.73, 1.76) | 1.69 (1.68, 1.71) | 1.76 (1.70, 1.82) | <0.0001 |
| Arithmetic mean | 1.82 (1.82, 1.82) | 1.84 (1.84, 1.85) | 1.86 (1.83, 1.89) | 1.88 (1.86, 1.90) | 1.82 (1.80, 1.84) | 1.89 (1.81, 1.98) | <0.0001 |
| **Total bilirubin, µmol/L** |  |  |  |  |  |  |  |
| Participants, n | 207,772 | 208,454 | 4,751 | 9,884 | 6,420 | 398 |  |
| Model 1 | 8.27 (8.26, 8.29) | 8.50 (8.49, 8.51) | 8.65 (8.56, 8.74) | 8.81 (8.75, 8.88) | 8.37 (8.30, 8.45) | 8.57 (8.26, 8.89) | <0.0001 |
| Model 2 | 8.31 (8.29, 8.32) | 8.48 (8.47, 8.49) | 8.51 (8.42, 8.60) | 8.66 (8.60, 8.73) | 8.24 (8.17, 8.32) | 8.36 (8.06, 8.67) | <0.0001 |
| Model 3 | 8.31 (8.30, 8.33)^a^ | 8.47 (8.46, 8.49)^b^ | 8.55 (8.46, 8.64)^bc^ | 8.64 (8.58, 8.70)^c^ | 8.25 (8.18, 8.33)^a^ | 8.46 (8.16, 8.77)^abc^ | <0.0001 |
| Model 4 | 8.32 (8.30, 8.33) | 8.47 (8.46, 8.49) | 8.54 (8.45, 8.63) | 8.63 (8.57, 8.69) | 8.24 (8.17, 8.32) | 8.44 (8.15, 8.76) | <0.0001 |
| Arithmetic mean | 9.04 (9.02, 9.06) | 9.22 (9.20, 9.24) | 9.31 (9.19, 9.43) | 9.40 (9.32, 9.48) | 8.96 (8.85, 9.06) | 9.24 (8.83, 9.66) | <0.0001 |
| **Gamma glutamyltransferase, U/L** |  |  |  |  |  |  |  |
| Participants, n | 208,604 | 209,196 | 4,762 | 9,916 | 6,445 | 398 |  |
| Model 1 | 30.4 (30.3, 30.5) | 28.2 (28.1, 28.3) | 25.0 (24.6, 25.5) | 24.2 (23.9, 24.5) | 23.9 (23.5, 24.2) | 20.7 (19.6, 22.0) | <0.0001 |
| Model 2 | 29.9 (29.8, 30.0) | 28.5 (28.4, 28.5) | 26.5 (26.1, 27.0) | 25.8 (25.5, 26.1) | 25.3 (24.9, 25.6) | 22.5 (21.3, 23.8) | <0.0001 |
| Model 3 | 29.6 (29.6, 29.7)^a^ | 28.7 (28.6, 28.7)^b^ | 27.1 (26.6, 27.5)^c^ | 26.1 (25.8, 26.4)^d^ | 25.8 (25.4, 26.1)^d^ | 23.5 (22.2, 24.8)^e^ | <0.0001 |
| Model 4 | 29.6 (29.6, 29.7) | 28.7 (28.6, 28.8) | 27.2 (26.8, 27.6) | 26.2 (25.9, 26.5) | 25.8 (25.5, 26.2) | 23.6 (22.4, 25.0) | <0.0001 |
| Arithmetic mean | 38.4 (38.2, 38.6) | 36.6 (36.4, 36.8) | 34.5 (33.4, 35.7) | 33.4 (32.6, 34.2) | 32.7 (31.7, 33.7) | 29.8 (25.8, 33.8) | <0.0001 |
| **Alanine aminotransferase, U/L** |  |  |  |  |  |  |  |
| Participants, n | 208,628 | 209,244 | 4,767 | 9,918 | 6,441 | 399 |  |
| Model 1 | 21.5 (21.4, 21.5) | 20.7 (20.7, 20.7) | 19.9 (19.7, 20.2) | 19.2 (19.0, 19.3) | 18.4 (18.2, 18.6) | 17.9 (17.2, 18.7) | <0.0001 |
| Model 2 | 21.2 (21.2, 21.2) | 20.9 (20.8, 20.9) | 20.9 (20.7, 21.2) | 20.2 (20.1, 20.4) | 19.3 (19.1, 19.5) | 19.3 (18.6, 20.1) | <0.0001 |
| Model 3 | 21.2 (21.1, 21.2)^a^ | 20.9 (20.8, 20.9)^b^ | 21.0 (20.7, 21.2)^ab^ | 20.2 (20.1, 20.4)^c^ | 19.3 (19.1, 19.5)^d^ | 19.4 (18.6, 20.2)^cd^ | <0.0001 |
| Model 4 | 21.2 (21.1, 21.2) | 20.9 (20.8, 20.9) | 21.0 (20.8, 21.3) | 20.2 (20.1, 20.4) | 19.3 (19.1, 19.5) | 19.4 (18.7, 20.2) | <0.0001 |
| Arithmetic mean | 23.8 (23.8, 23.9) | 23.4 (23.3, 23.4) | 23.5 (23.1, 23.8) | 22.7 (22.4, 22.9) | 21.6 (21.3, 22.0) | 21.7 (20.4, 23.0) | <0.0001 |
| **Aspartate aminotransferase, U/L** |  |  |  |  |  |  |  |
| Participants, n | 207,861 | 208,566 | 4,755 | 9,895 | 6,422 | 398 |  |
| Model 1 | 25.1 (25.0, 25.1) | 25.0 (25.0, 25.1) | 25.7 (25.6, 25.9) | 25.2 (25.1, 25.4) | 24.8 (24.7, 25.0) | 25.4 (24.7, 26.0) | <0.0001 |
| Model 2 | 25.0 (25.0, 25.0) | 25.1 (25.1, 25.1) | 26.0 (25.8, 26.2) | 25.5 (25.3, 25.6) | 25.0 (24.9, 25.2) | 25.6 (25.0, 26.3) | <0.0001 |
| Model 3 | 25.0 (24.9, 25.0)^a^ | 25.1 (25.1, 25.1)^b^ | 26.0 (25.8, 26.2)^c^ | 25.5 (25.3, 25.6)^d^ | 25.1 (24.9, 25.2)^ab^ | 25.7 (25.1, 26.4)^abcd^ | <0.0001 |
| Model 4 | 25.0 (24.9, 25.0) | 25.1 (25.1, 25.1) | 26.0 (25.8, 26.2) | 25.4 (25.3, 25.6) | 25.0 (24.9, 25.2) | 25.7 (25.0, 26.4) | <0.0001 |
| Arithmetic mean | 26.1 (26.1, 26.2) | 26.2 (26.2, 26.3) | 27.1 (26.9, 27.4) | 26.5 (26.3, 26.7) | 26.2 (25.9, 26.4) | 26.8 (25.8, 27.8) | <0.0001 |

1. Unless otherwise specified, all estimates represent adjusted geometric means and 95% CIs, or if stated, adjusted arithmetic means and 95% CIs. Model 1 was adjusted for sex, age (5 years categories) and fasting status (0-1, 2, 3, 4, 5, 6-7, ≥8 hours); model 2 was additionally adjusted for BMI (<20, 20.0-22.4, 22.5-24.9, 25.0-27.4, 27.5-29.9, 30.0-32.4, 32.5-34.9, ≥35.0 kg/m^2^, unknown), model 3 was additionally adjusted for alcohol consumption (<1, 1-7, 8-15, ≥16 g/d, unknown) and smoking status (never, previous, current <15 cigarettes/day, current ≥15 cigarettes/day, unknown), model 4 was additionally adjusted for physical activity (low <10 excess MET hours/week, moderate 10-49 excess MET hours/week, high ≥50 excess MET hours/week, unknown). Arithmetic means were based on model 3 unless otherwise specified.
2. Includes participants who consume any red or processed meat, regardless of whether they consume poultry, fish, or dairy. Cut-offs of regular and low consumption determined based on consumption of red and processed meat (beef, lamb, pork, processed meat) as reported on the touchscreen questionnaire.
3. Represents p for heterogeneity across the six diet groups based on Wald tests. Groups that do not share a superscript letter (ordered alphabetically from left to right) were significantly different at the 5% level from post hoc Bonferroni-corrected pairwise comparisons after linear regression (model 3).

**Supplemental Table 25**: **Liver-related** serum biomarker concentrations (numbers, adjusted means and 95% CIs)^1^ in **white British women** by diet group in the UK Biobank.

| **Liver-related serum biomarker** | **Regular meat eaters (>3 times/week)^2^** | **Low meat eaters (>3 times/week)^2^** | **Poultry eaters** | **Fish eaters** | **Vegetarians** | **Vegans** | **p-het^3^** |
| --- | --- | --- | --- | --- | --- | --- | --- |
| **Albumin, g/L** |  |  |  |  |  |  |  |
| Participants, n | 81,540 | 121,357 | 3,348 | 6,465 | 3,922 | 216 |  |
| Model 1 | 44.9 (44.8, 44.9) | 44.9 (44.9, 45.0) | 44.9 (44.9, 45.0) | 45.0 (45.0, 45.1) | 44.8 (44.7, 44.9) | 44.8 (44.4, 45.1) | <0.0001 |
| Model 2 | 44.9 (44.9, 44.9) | 44.9 (44.9, 44.9) | 44.8 (44.7, 44.9) | 44.9 (44.8, 44.9) | 44.7 (44.6, 44.7) | 44.6 (44.2, 44.9) | <0.0001 |
| Model 3 | 44.9 (44.9, 44.9)^a^ | 44.9 (44.9, 44.9)^a^ | 44.8 (44.7, 44.9)^ab^ | 44.9 (44.8, 44.9)^a^ | 44.7 (44.6, 44.8)^b^ | 44.7 (44.3, 45.0)^ab^ | <0.0001 |
| Model 4 | 44.9 (44.9, 44.9) | 44.9 (44.9, 44.9) | 44.8 (44.7, 44.9) | 44.9 (44.8, 44.9) | 44.7 (44.6, 44.8) | 44.7 (44.3, 45.0) | <0.0001 |
| Arithmetic mean | 45.0 (45.0, 45.0) | 45.0 (45.0, 45.0) | 44.9 (44.8, 45.0) | 44.9 (44.9, 45.0) | 44.8 (44.7, 44.8) | 44.7 (44.4, 45.0) | <0.0001 |
| **Direct bilirubin, µmol/L** |  |  |  |  |  |  |  |
| Participants, n | 69,113 | 105,022 | 2,959 | 5,792 | 3,398 | 187 |  |
| Model 1 | 1.55 (1.54, 1.55) | 1.56 (1.56, 1.56) | 1.57 (1.55, 1.58) | 1.60 (1.59, 1.61) | 1.55 (1.53, 1.57) | 1.59 (1.52, 1.66) | <0.0001 |
| Model 2 | 1.55 (1.55, 1.56) | 1.56 (1.55, 1.56) | 1.55 (1.53, 1.56) | 1.58 (1.57, 1.59) | 1.53 (1.51, 1.55) | 1.56 (1.49, 1.63) | <0.0001 |
| Model 3 | 1.55 (1.55, 1.55)^ab^ | 1.56 (1.55, 1.56)^a^ | 1.55 (1.53, 1.57)^abc^ | 1.58 (1.56, 1.59)^c^ | 1.53 (1.51, 1.55)^b^ | 1.56 (1.49, 1.64)^abc^ | <0.0001 |
| Model 4 | 1.55 (1.55, 1.55) | 1.56 (1.55, 1.56) | 1.55 (1.53, 1.57) | 1.58 (1.56, 1.59) | 1.53 (1.51, 1.55) | 1.56 (1.49, 1.63) | 0.0001 |
| Arithmetic mean | 1.65 (1.64, 1.65) | 1.65 (1.65, 1.66) | 1.64 (1.62, 1.67) | 1.68 (1.66, 1.69) | 1.62 (1.60, 1.64) | 1.66 (1.56, 1.76) | 0.006 |
| **Total bilirubin, µmol/L** |  |  |  |  |  |  |  |
| Participants, n | 89,385 | 132,797 | 3,689 | 7,130 | 4,333 | 229 |  |
| Model 1 | 7.46 (7.44, 7.48) | 7.62 (7.60, 7.63) | 7.71 (7.62, 7.81) | 7.89 (7.82, 7.95) | 7.50 (7.42, 7.58) | 7.58 (7.23, 7.94) | <0.0001 |
| Model 2 | 7.51 (7.49, 7.53) | 7.60 (7.59, 7.62) | 7.57 (7.48, 7.66) | 7.73 (7.67, 7.80) | 7.37 (7.29, 7.45) | 7.37 (7.04, 7.73) | <0.0001 |
| Model 3 | 7.50 (7.48, 7.52)^ab^ | 7.61 (7.59, 7.62)^c^ | 7.61 (7.53, 7.70)^bcd^ | 7.72 (7.66, 7.79)^d^ | 7.39 (7.32, 7.47)^a^ | 7.46 (7.12, 7.81)^abcd^ | <0.0001 |
| Model 4 | 7.50 (7.48, 7.52) | 7.60 (7.59, 7.62) | 7.61 (7.52, 7.69) | 7.71 (7.65, 7.78) | 7.39 (7.31, 7.47) | 7.45 (7.11, 7.81) | <0.0001 |
| Arithmetic mean | 8.09 (8.07, 8.11) | 8.19 (8.17, 8.21) | 8.17 (8.05, 8.29) | 8.32 (8.23, 8.40) | 7.93 (7.82, 8.04) | 7.98 (7.51, 8.45) | <0.0001 |
| **Gamma glutamyltransferase, U/L** |  |  |  |  |  |  |  |
| Participants, n | 89,741 | 133,277 | 3,701 | 7,156 | 4,350 | 229 |  |
| Model 1 | 25.2 (25.1, 25.3) | 23.7 (23.6, 23.7) | 21.3 (20.9, 21.7) | 20.7 (20.4, 21.0) | 20.3 (20.0, 20.7) | 18.3 (17.0, 19.7) | <0.0001 |
| Model 2 | 24.7 (24.6, 24.8) | 23.8 (23.8, 23.9) | 22.3 (21.9, 22.7) | 21.8 (21.5, 22.0) | 21.2 (20.9, 21.6) | 19.4 (18.1, 20.8) | <0.0001 |
| Model 3 | 24.6 (24.5, 24.7)^a^ | 23.9 (23.8, 24.0)^b^ | 22.5 (22.1, 22.9)^c^ | 21.9 (21.6, 22.2)^cd^ | 21.5 (21.1, 21.8)^d^ | 19.9 (18.6, 21.4)^d^ | <0.0001 |
| Model 4 | 24.6 (24.5, 24.6) | 23.9 (23.8, 24.0) | 22.6 (22.2, 23.0) | 21.9 (21.7, 22.2) | 21.5 (21.2, 21.9) | 20.0 (18.6, 21.5) | <0.0001 |
| Arithmetic mean | 31.0 (30.8, 31.2) | 30.0 (29.8, 30.2) | 27.8 (26.7, 28.9) | 27.1 (26.3, 27.9) | 26.6 (25.6, 27.6) | 24.5 (20.2, 28.7) | <0.0001 |
| **Alanine aminotransferase, U/L** |  |  |  |  |  |  |  |
| Participants, n | 89,775 | 133,338 | 3,705 | 7,156 | 4,349 | 230 |  |
| Model 1 | 18.7 (18.7, 18.8) | 18.2 (18.1, 18.2) | 17.6 (17.3, 17.8) | 17.0 (16.9, 17.2) | 16.4 (16.2, 16.6) | 16.3 (15.5, 17.2) | <0.0001 |
| Model 2 | 18.5 (18.4, 18.5) | 18.2 (18.2, 18.3) | 18.2 (18.0, 18.5) | 17.7 (17.6, 17.9) | 17.0 (16.8, 17.2) | 17.2 (16.3, 18.1) | <0.0001 |
| Model 3 | 18.5 (18.4, 18.5)^a^ | 18.3 (18.2, 18.3)^b^ | 18.3 (18.0, 18.5)^ab^ | 17.7 (17.5, 17.9)^c^ | 17.0 (16.8, 17.2)^d^ | 17.2 (16.3, 18.1)^abcd^ | <0.0001 |
| Model 4 | 18.5 (18.4, 18.5) | 18.3 (18.2, 18.3) | 18.3 (18.0, 18.5) | 17.7 (17.6, 17.9) | 17.0 (16.8, 17.2) | 17.2 (16.3, 18.1) | <0.0001 |
| Arithmetic mean | 20.5 (20.4, 20.6) | 20.2 (20.2, 20.3) | 20.0 (19.7, 20.4) | 19.5 (19.3, 19.8) | 18.7 (18.4, 19.1) | 19.1 (17.6, 20.6) | <0.0001 |
| **Aspartate aminotransferase, U/L** |  |  |  |  |  |  |  |
| Participants, n | 89,435 | 132,887 | 3,693 | 7,140 | 4,335 | 229 |  |
| Model 1 | 23.5 (23.4, 23.5) | 23.6 (23.5, 23.6) | 24.2 (24.0, 24.4) | 23.9 (23.8, 24.1) | 23.6 (23.5, 23.8) | 24.5 (23.7, 25.3) | <0.0001 |
| Model 2 | 23.4 (23.4, 23.4) | 23.6 (23.6, 23.6) | 24.3 (24.1, 24.5) | 24.0 (23.9, 24.2) | 23.7 (23.5, 23.9) | 24.6 (23.8, 25.4) | <0.0001 |
| Model 3 | 23.4 (23.4, 23.4)^a^ | 23.6 (23.6, 23.6)^b^ | 24.3 (24.1, 24.5)^c^ | 24.0 (23.8, 24.1)^cd^ | 23.7 (23.5, 23.9)^abd^ | 24.5 (23.7, 25.4)^abcd^ | <0.0001 |
| Model 4 | 23.4 (23.4, 23.4) | 23.6 (23.6, 23.6) | 24.3 (24.1, 24.5) | 24.0 (23.8, 24.1) | 23.7 (23.5, 23.8) | 24.5 (23.7, 25.3) | <0.0001 |
| Arithmetic mean | 24.4 (24.3, 24.4) | 24.6 (24.5, 24.6) | 25.2 (24.9, 25.5) | 24.9 (24.7, 25.1) | 24.6 (24.3, 24.9) | 25.6 (24.4, 26.8) | <0.0001 |

1. Unless otherwise specified, all estimates represent adjusted geometric means and 95% CIs, or if stated, adjusted arithmetic means and 95% CIs. Model 1 was adjusted for age (5 years categories) and fasting status (0-1, 2, 3, 4, 5, 6-7, ≥8 hours); model 2 was additionally adjusted for BMI (<20, 20.0-22.4, 22.5-24.9, 25.0-27.4, 27.5-29.9, 30.0-32.4, 32.5-34.9, ≥35.0 kg/m^2^, unknown), model 3 was additionally adjusted for alcohol consumption (<1, 1-7, 8-15, ≥16 g/d, unknown) and smoking status (never, previous, current <15 cigarettes/day, current ≥15 cigarettes/day, unknown), model 4 was additionally adjusted for physical activity (low <10 excess MET hours/week, moderate 10-49 excess MET hours/week, high ≥50 excess MET hours/week, unknown). Arithmetic means were based on model 3 unless otherwise specified.
2. Includes participants who consume any red or processed meat, regardless of whether they consume poultry, fish, or dairy. Cut-offs of regular and low consumption determined based on consumption of red and processed meat (beef, lamb, pork, processed meat) as reported on the touchscreen questionnaire.
3. Represents p for heterogeneity across the six diet groups based on Wald tests. Groups that do not share a superscript letter (ordered alphabetically from left to right) were significantly different at the 5% level from post hoc Bonferroni-corrected pairwise comparisons after linear regression (model 3).

**Supplemental Table 26**: **Liver-related** serum biomarker concentrations (numbers, adjusted means and 95% CIs)^1^ in **white British men** by diet group in the UK Biobank.

| **Liver-related serum biomarker** | **Regular meat eaters (>3 times/week)^2^** | **Low meat eaters (>3 times/week)^2^** | **Poultry eaters** | **Fish eaters** | **Vegetarians** | **Vegans** | **p-het^3^** |
| --- | --- | --- | --- | --- | --- | --- | --- |
| **Albumin, g/L** |  |  |  |  |  |  |  |
| Participants, n | 109,941 | 70,118 | 973 | 2,546 | 1,934 | 153 |  |
| Model 1 | 45.4 (45.4, 45.4) | 45.5 (45.5, 45.5) | 45.5 (45.4, 45.7) | 45.5 (45.4, 45.6) | 45.3 (45.2, 45.4) | 45.1 (44.7, 45.5) | <0.0001 |
| Model 2 | 45.4 (45.4, 45.4) | 45.5 (45.5, 45.5) | 45.5 (45.3, 45.7) | 45.5 (45.4, 45.6) | 45.2 (45.1, 45.3) | 45.1 (44.7, 45.5) | <0.0001 |
| Model 3 | 45.4 (45.4, 45.4)^a^ | 45.5 (45.5, 45.5)^b^ | 45.5 (45.4, 45.7)^abc^ | 45.5 (45.4, 45.6)^ab^ | 45.2 (45.1, 45.4)^c^ | 45.2 (44.8, 45.6)^abc^ | <0.0001 |
| Model 4 | 45.4 (45.4, 45.4) | 45.5 (45.5, 45.5) | 45.5 (45.4, 45.7) | 45.5 (45.4, 45.6) | 45.2 (45.1, 45.3) | 45.2 (44.8, 45.6) | <0.0001 |
| Arithmetic mean | 45.5 (45.5, 45.5) | 45.6 (45.6, 45.6) | 45.6 (45.4, 45.8) | 45.5 (45.4, 45.6) | 45.3 (45.2, 45.4) | 45.2 (44.8, 45.6) | <0.0001 |
| **Direct bilirubin, µmol/L** |  |  |  |  |  |  |  |
| Participants, n | 110,339 | 71,285 | 1,005 | 2,604 | 1,939 | 164 |  |
| Model 1 | 1.85 (1.85, 1.86) | 1.90 (1.89, 1.90) | 1.98 (1.94, 2.03) | 1.97 (1.94, 1.99) | 1.89 (1.86, 1.92) | 2.01 (1.90, 2.13) | <0.0001 |
| Model 2 | 1.85 (1.85, 1.86) | 1.89 (1.89, 1.90) | 1.97 (1.92, 2.01) | 1.95 (1.92, 1.98) | 1.87 (1.84, 1.90) | 1.98 (1.88, 2.10) | <0.0001 |
| Model 3 | 1.86 (1.85, 1.86)^a^ | 1.89 (1.89, 1.90)^b^ | 1.96 (1.92, 2.01)^c^ | 1.94 (1.92, 1.97)^c^ | 1.87 (1.84, 1.90)^ab^ | 1.99 (1.88, 2.10)^abc^ | <0.0001 |
| Model 4 | 1.86 (1.85, 1.86) | 1.89 (1.89, 1.90) | 1.96 (1.92, 2.01) | 1.94 (1.92, 1.97) | 1.87 (1.84, 1.90) | 1.99 (1.88, 2.10) | <0.0001 |
| Arithmetic mean | 2.00 (1.99, 2.00) | 2.04 (2.03, 2.04) | 2.14 (2.08, 2.20) | 2.09 (2.06, 2.13) | 2.01 (1.97, 2.06) | 2.14 (2.00, 2.28) | <0.0001 |
| **Total bilirubin, µmol/L** |  |  |  |  |  |  |  |
| Participants, n | 118,387 | 75,657 | 1,062 | 2,754 | 2,087 | 169 |  |
| Model 1 | 9.38 (9.35, 9.40) | 9.69 (9.66, 9.72) | 10.08 (9.85, 10.31) | 10.09 (9.94, 10.23) | 9.54 (9.38, 9.70) | 9.95 (9.40, 10.54) | <0.0001 |
| Model 2 | 9.39 (9.37, 9.41) | 9.67 (9.64, 9.70) | 9.97 (9.75, 10.20) | 9.98 (9.84, 10.12) | 9.44 (9.29, 9.59) | 9.81 (9.26, 10.39) | <0.0001 |
| Model 3 | 9.41 (9.39, 9.43)^a^ | 9.64 (9.61, 9.66)^bc^ | 9.95 (9.73, 10.18)^cd^ | 9.92 (9.78, 10.06)^d^ | 9.42 (9.27, 9.58)^ab^ | 9.89(9.34, 10.47)^abcd^ | <0.0001 |
| Model 4 | 9.42 (9.40, 9.44) | 9.64 (9.61, 9.66) | 9.95 (9.72, 10.18) | 9.91 (9.77, 10.05) | 9.41 (9.26, 9.57) | 9.86 (9.32, 10.43) | <0.0001 |
| Arithmetic mean | 10.2 (10.2, 10.2) | 10.5 (10.4, 10.6) | 10.9 (10.6, 11.2) | 10.8 (10.6, 11.0) | 10.2 (9.97, 10.4) | 10.8 (10.1, 11.5) | <0.0001 |
| **Gamma glutamyltransferase, U/L** |  |  |  |  |  |  |  |
| Participants, n | 118,863 | 75,919 | 1,061 | 2,760 | 2,095 | 169 |  |
| Model 1 | 37.8 (37.7, 37.9) | 34.6 (34.5, 34.8) | 29.5 (28.4, 30.6) | 28.9 (28.2, 29.5) | 28.9 (28.2, 29.7) | 23.9 (21.8, 26.1) | <0.0001 |
| Model 2 | 37.3 (37.2, 37.5) | 35.1 (34.9, 35.2) | 31.9 (30.8, 33.0) | 31.4 (30.7, 32.1) | 31.4 (30.6, 32.2) | 26.9 (24.7, 29.4) | <0.0001 |
| Model 3 | 36.9 (36.8, 37.0)^a^ | 35.6 (35.4, 35.7)^b^ | 33.6 (32.4, 34.7)^c^ | 32.1 (31.5, 32.8)^cd^ | 32.4 (31.6, 33.2)^cd^ | 28.7 (26.3, 31.2)^d^ | <0.0001 |
| Model 4 | 36.9 (36.8, 37.0) | 35.6 (35.5, 35.7) | 33.8 (32.7, 35.0) | 32.3 (31.6, 33.0) | 32.5 (31.7, 33.3) | 28.8 (26.5, 31.4) | <0.0001 |
| Arithmetic mean | 46.9 (46.6, 47.1) | 44.4 (44.1, 44.8) | 41.9 (39.0, 44.8) | 40.4 (38.6, 42.2) | 39.7 (37.7, 41.8) | 36.1 (28.9, 43.3) | <0.0001 |
| **Alanine aminotransferase, U/L** |  |  |  |  |  |  |  |
| Participants, n | 118,853 | 75,906 | 1,062 | 2,762 | 2,092 | 169 |  |
| Model 1 | 25.2 (25.1, 25.2) | 24.2 (24.1, 24.3) | 22.8 (22.2, 23.4) | 22.1 (21.8, 22.5) | 21.1 (20.7, 21.5) | 19.9 (18.7, 21.3) | <0.0001 |
| Model 2 | 24.9 (24.8, 25.0) | 24.4 (24.4, 24.5) | 24.5 (23.9, 25.1) | 23.9 (23.5, 24.3) | 22.8 (22.4, 23.2) | 22.4 (21.1, 23.8) | <0.0001 |
| Model 3 | 24.9 (24.8, 25.0)^a^ | 24.5 (24.4, 24.5)^b^ | 24.6 (24.0, 25.2)^ab^ | 23.9 (23.5, 24.3)^b^ | 22.8 (22.5, 23.2)^c^ | 22.6 (21.2, 24.0)^bc^ | <0.0001 |
| Model 4 | 24.9 (24.8, 24.9) | 24.5 (24.4, 24.5) | 24.7 (24.1, 25.3) | 23.9 (23.6, 24.3) | 22.9 (22.5, 23.3) | 22.6 (21.3, 24.0) | <0.0001 |
| Arithmetic mean | 27.7 (27.6, 27.8) | 27.1 (27.0, 27.2) | 27.5 (26.6, 28.4) | 26.7 (26.1, 27.2) | 25.3 (24.7, 25.9) | 25.0 (22.8, 27.1) | <0.0001 |
| **Aspartate aminotransferase, U/L** |  |  |  |  |  |  |  |
| Participants, n | 118,426 | 75,679 | 1,062 | 2,755 | 2,087 | 169 |  |
| Model 1 | 27.0 (27.0, 27.1) | 26.9 (26.8, 26.9) | 27.6 (27.2, 28.1) | 26.8 (26.6, 27.1) | 26.3 (26.0, 26.7) | 26.3 (25.2, 27.4) | <0.0001 |
| Model 2 | 27.0 (26.9, 27.0) | 27.0 (26.9, 27.0) | 28.2 (27.7, 28.6) | 27.4 (27.1, 27.6) | 26.8 (26.5, 27.2) | 27.0 (25.9, 28.2) | <0.0001 |
| Model 3 | 26.9 (26.9, 27.0)^a^ | 27.0 (26.9, 27.1)^ab^ | 28.3 (27.9, 28.8)^c^ | 27.4 (27.1, 27.7)^b^ | 26.9 (26.6, 27.2)^ab^ | 27.3 (26.2, 28.4)^abc^ | <0.0001 |
| Model 4 | 26.9 (26.9, 27.0) | 27.0 (26.9, 27.1) | 28.3 (27.8, 28.8) | 27.4 (27.1, 27.6) | 26.9 (26.6, 27.2) | 27.2 (26.1, 28.4) | <0.0001 |
| Arithmetic mean | 28.2 (28.1, 28.3) | 28.2 (28.1, 28.3) | 29.8 (29.1, 30.5) | 28.6 (28.2, 29.0) | 28.2 (27.7, 28.7) | 28.1 (26.5, 29.8) | 0.0002 |

1. Unless otherwise specified, all estimates represent adjusted geometric means and 95% CIs, or if stated, adjusted arithmetic means and 95% CIs. Model 1 was adjusted for age (5 years categories) and fasting status (0-1, 2, 3, 4, 5, 6-7, ≥8 hours); model 2 was additionally adjusted for BMI (<20, 20.0-22.4, 22.5-24.9, 25.0-27.4, 27.5-29.9, 30.0-32.4, 32.5-34.9, ≥35.0 kg/m^2^, unknown), model 3 was additionally adjusted for alcohol consumption (<1, 1-7, 8-15, ≥16 g/d, unknown) and smoking status (never, previous, current <15 cigarettes/day, current ≥15 cigarettes/day, unknown), model 4 was additionally adjusted for physical activity (low <10 excess MET hours/week, moderate 10-49 excess MET hours/week, high ≥50 excess MET hours/week, unknown). Arithmetic means were based on model 3 unless otherwise specified.
2. Includes participants who consume any red or processed meat, regardless of whether they consume poultry, fish, or dairy. Cut-offs of regular and low consumption determined based on consumption of red and processed meat (beef, lamb, pork, processed meat) as reported on the touchscreen questionnaire.
3. Represents p for heterogeneity across the six diet groups based on Wald tests.﻿ Groups that do not share a superscript letter (ordered alphabetically from left to right) were significantly different at the 5% level from post hoc Bonferroni-corrected pairwise comparisons after linear regression (model 3).

**Supplemental Table 27**: **Liver-related** serum biomarker concentrations (numbers, adjusted means and 95% CIs)^1^ in **British Indian participants** by diet group in the UK Biobank.

| **Liver-related serum biomarker** | **All participants** | | |  | **Women** | | |  | **Men** | | |
| --- | --- | --- | --- | --- | --- | --- | --- | --- | --- | --- | --- |
|  | **Meat-eaters** | **Vegetarians** | **p-het^2^** |  | **Meat-eaters** | **Vegetarians** | **p-het^2^** |  | **Meat-eaters** | **Vegetarians** | **p-het^2^** |
| **Albumin, g/L** |  |  |  |  |  |  |  |  |  |  |  |
| Participants, n | 3,493 | 1,204 |  |  | 1,447 | 775 |  |  | 2,046 | 429 |  |
| Model 1 | 44.7 (44.6, 44.8) | 44.4 (44.3, 44.6) | 0.001 |  | 44.2 (44.0, 44.3) | 43.8 (43.6, 44.0) | 0.001 |  | 45.2 (45.1, 45.4) | 45.0 (44.8, 45.3) | 0.14 |
| Model 2 | 44.7 (44.6, 44.8) | 44.4 (44.3, 44.6) | 0.0004 |  | 44.2 (44.1, 44.3) | 43.8 (43.6, 43.9) | 0.0002 |  | 45.2 (45.1, 45.4) | 45.0 (44.8, 45.3) | 0.16 |
| Model 3 | 44.7 (44.6, 44.8) | 44.4 (44.3, 44.6) | 0.003 |  | 44.2 (44.0, 44.3) | 43.8 (43.6, 44.0) | 0.002 |  | 45.2 (45.1, 45.4) | 45.1 (44.8, 45.4) | 0.39 |
| Model 4 | 44.7 (44.6, 44.8) | 44.4 (44.3, 44.6) | 0.002 |  | 44.2 (44.0, 44.3) | 43.8 (43.6, 44.0) | 0.001 |  | 45.2 (45.1, 45.4) | 45.1 (44.8, 45.4) | 0.34 |
| Arithmetic mean | 44.8 (44.7, 44.9) | 44.5 (44.4, 44.7) | 0.003 |  | 44.3 (44.1, 44.4) | 43.9 (43.7, 44.1) | 0.002 |  | 45.3 (45.2, 45.4) | 45.2 (44.9, 45.4) | 0.32 |
| **Direct bilirubin, µmol/L** |  |  |  |  |  |  |  |  |  |  |  |
| Participants, n | 3,224 | 1,050 |  |  | 1,170 | 617 |  |  | 2,054 | 433 |  |
| Model 1 | 1.71 (1.69, 1.74) | 1.70 (1.66, 1.74) | 0.51 |  | 1.49 (1.46, 1.51) | 1.49 (1.46, 1.53) | 0.88 |  | 1.90 (1.87, 1.93) | 1.86 (1.80, 1.93) | 0.40 |
| Model 2 | 1.72 (1.69, 1.74) | 1.70 (1.66, 1.73) | 0.41 |  | 1.49 (1.46, 1.52) | 1.49 (1.45, 1.53) | 0.97 |  | 1.90 (1.87, 1.93) | 1.86 (1.80, 1.93) | 0.35 |
| Model 3 | 1.71 (1.69, 1.73) | 1.71 (1.68, 1.75) | 0.83 |  | 1.49 (1.46, 1.51) | 1.49 (1.46, 1.53) | 0.78 |  | 1.89 (1.86, 1.92) | 1.89 (1.82, 1.97) | 0.94 |
| Model 4 | 1.71 (1.69, 1.73) | 1.71 (1.68, 1.75) | 0.83 |  | 1.49 (1.46, 1.51) | 1.49 (1.46, 1.53) | 0.85 |  | 1.89 (1.86, 1.92) | 1.89 (1.82, 1.97) | 0.90 |
| Arithmetic mean | 1.84 (1.82, 1.87) | 1.84 (1.79, 1.89) | 0.90 |  | 1.57 (1.54, 1.61) | 1.57 (1.52, 1.62) | 0.97 |  | 2.04 (2.00, 2.08) | 2.04 (1.94, 2.13) | 0.93 |
| **Total bilirubin, µmol/L** |  |  |  |  |  |  |  |  |  |  |  |
| Participants, n | 3,808 | 1,338 |  |  | 1,595 | 866 |  |  | 2,213 | 472 |  |
| Model 1 | 8.19 (8.09, 8.29) | 8.03 (7.86, 8.19) | 0.10 |  | 6.99 (6.87, 7.12) | 6.89 (6.73, 7.06) | 0.35 |  | 9.47 (9.31, 9.63) | 9.20 (8.88, 9.54) | 0.16 |
| Model 2 | 8.20 (8.10, 8.30) | 8.02 (7.85, 8.18) | 0.07 |  | 7.00 (6.87, 7.12) | 6.89 (6.73, 7.05) | 0.31 |  | 9.47 (9.31, 9.63) | 9.20 (8.88, 9.54) | 0.16 |
| Model 3 | 8.15 (8.05, 8.25) | 8.14 (7.96, 8.31) | 0.88 |  | 6.97 (6.85, 7.10) | 6.93 (6.77, 7.11) | 0.74 |  | 9.41 (9.26, 9.57) | 9.45 (9.09, 9.82) | 0.87 |
| Model 4 | 8.15 (8.05, 8.25) | 8.14 (7.96, 8.31) | 0.88 |  | 6.97 (6.85, 7.10) | 6.93 (6.76, 7.10) | 0.72 |  | 9.41 (9.26, 9.57) | 9.45 (9.10, 9.82) | 0.85 |
| Arithmetic mean | 8.93 (8.80, 9.06) | 8.90 (8.67, 9.14) | 0.85 |  | 7.48 (7.33, 7.64) | 7.42 (7.20, 7.63) | 0.63 |  | 10.26 (10.05, 10.46) | 10.31 (9.84, 10.77) | 0.84 |
| **Gamma glutamyltransferase, U/L** |  |  |  |  |  |  |  |  |  |  |  |
| Participants, n | 3,821 | 1,343 |  |  | 1,604 | 869 |  |  | 2,217 | 474 |  |
| Model 1 | 30.5 (30.0, 31.1) | 24.9 (24.2, 25.7) | <0.0001 |  | 24.7 (24.1, 25.4) | 21.5 (20.7, 22.3) | <0.0001 |  | 36.9 (36.0, 37.8) | 27.5 (26.1, 29.0) | <0.0001 |
| Model 2 | 30.4 (29.9, 31.0) | 25.1 (24.3, 25.8) | <0.0001 |  | 24.7 (24.1, 25.3) | 21.5 (20.8, 22.3) | <0.0001 |  | 36.8 (35.9, 37.7) | 27.8 (26.4, 29.3) | <0.0001 |
| Model 3 | 30.0 (29.5, 30.5) | 26.2 (25.4, 27.0) | <0.0001 |  | 24.6 (24.0, 25.3) | 21.6 (20.9, 22.4) | <0.0001 |  | 36.0 (35.1, 36.8) | 30.9 (29.3, 32.7) | <0.0001 |
| Model 4 | 30.0 (29.5, 30.5) | 26.2 (25.4, 27.0) | <0.0001 |  | 24.6 (24.0, 25.3) | 21.6 (20.9, 22.4) | <0.0001 |  | 36.0 (35.1, 36.8) | 31.0 (29.3, 32.7) | <0.0001 |
| Arithmetic mean | 38.3 (37.0, 39.6) | 32.4 (30.1, 34.7) | <0.0001 |  | 30.1 (28.8, 31.3) | 25.3 (23.6, 27.0) | <0.0001 |  | 45.8 (43.7, 47.9) | 38.0 (33.1, 42.8) | 0.004 |
| **Alanine aminotransferase, U/L** |  |  |  |  |  |  |  |  |  |  |  |
| Participants, n | 3,817 | 1,341 |  |  | 1,603 | 868 |  |  | 2,214 | 473 |  |
| Model 1 | 21.6 (21.3, 21.9) | 19.8 (19.4, 20.3) | <0.0001 |  | 18.5 (18.1, 18.9) | 17.3 (16.8, 17.8) | 0.0001 |  | 24.8 (24.4, 25.3) | 22.2 (21.3, 23.0) | <0.0001 |
| Model 2 | 21.5 (21.2, 21.8) | 19.9 (19.5, 20.4) | <0.0001 |  | 18.5 (18.1, 18.9) | 17.3 (16.8, 17.8) | 0.0001 |  | 24.8 (24.3, 25.2) | 22.4 (21.6, 23.3) | <0.0001 |
| Model 3 | 21.5 (21.2, 21.8) | 20.1 (19.6, 20.6) | <0.0001 |  | 18.5 (18.1, 18.9) | 17.3 (16.8, 17.8) | 0.0005 |  | 24.7 (24.2, 25.1) | 23.0 (22.1, 23.9) | 0.003 |
| Model 4 | 21.5 (21.2, 21.8) | 20.1 (19.6, 20.6) | <0.0001 |  | 18.5 (18.1, 18.9) | 17.3 (16.8, 17.8) | 0.0004 |  | 24.7 (24.2, 25.1) | 22.9 (22.0, 23.9) | 0.002 |
| Arithmetic mean | 24.2 (23.8, 24.7) | 22.4 (21.6, 23.1) | <0.0001 |  | 20.6 (20.1, 21.2) | 18.9 (18.1, 19.7) | 0.001 |  | 27.5 (26.9, 28.1) | 25.6 (24.1, 27.0) | 0.02 |
| **Aspartate aminotransferase, U/L** |  |  |  |  |  |  |  |  |  |  |  |
| Participants, n | 3,810 | 1,338 |  |  | 1,597 | 866 |  |  | 2,213 | 472 |  |
| Model 1 | 25.4 (25.2, 25.6) | 24.4 (24.1, 24.8) | <0.0001 |  | 23.7 (23.4, 24.0) | 23.1 (22.7, 23.5) | 0.01 |  | 27.1 (26.7, 27.4) | 25.5 (24.9, 26.2) | <0.0001 |
| Model 2 | 25.4 (25.2, 25.6) | 24.5 (24.1, 24.8) | <0.0001 |  | 23.7 (23.4, 24.0) | 23.1 (22.7, 23.5) | 0.01 |  | 27.0 (26.7, 27.4) | 25.6 (25.0, 26.3) | 0.0001 |
| Model 3 | 25.3 (25.1, 25.5) | 24.7 (24.3, 25.1) | 0.008 |  | 23.7 (23.4, 24.0) | 23.1 (22.7, 23.6) | 0.04 |  | 26.9 (26.6, 27.2) | 26.2 (25.5, 26.9) | 0.07 |
| Model 4 | 25.3 (25.1, 25.5) | 24.7 (24.3, 25.1) | 0.01 |  | 23.7 (23.4, 24.0) | 23.2 (22.7, 23.6) | 0.05 |  | 26.9 (26.6, 27.2) | 26.2 (25.6, 26.9) | 0.10 |
| Arithmetic mean | 26.5 (26.2, 26.8) | 25.8 (25.2, 26.4) | 0.03 |  | 24.8 (24.3, 25.2) | 23.9 (23.3, 24.6) | 0.05 |  | 28.1 (27.7, 28.6) | 27.5 (26.5, 28.6) | 0.32 |

1. Unless otherwise specified, all estimates represent adjusted geometric means and 95% CIs, or if stated, adjusted arithmetic means and 95% CIs. Model 1 was adjusted for sex (for analyses with all participants), age (5 years categories) and fasting status (0-1, 2, 3, 4, 5, 6-7, ≥8 hours); model 2 was additionally adjusted for BMI (<20, 20.0-22.4, 22.5-24.9, 25.0-27.4, 27.5-29.9, 30.0-32.4, 32.5-34.9, ≥35.0 kg/m^2^, unknown), model 3 was additionally adjusted for alcohol consumption (<1, 1-7, 8-15, ≥16 g/d, unknown) and smoking status (never, previous, current <15 cigarettes/day, current ≥15 cigarettes/day, unknown), model 4 was additionally adjusted for physical activity (low <10 excess MET hours/week, moderate 10-49 excess MET hours/week, high ≥50 excess MET hours/week, unknown). Arithmetic means were based on model 3 unless otherwise specified.
2. Represents p for heterogeneity across the two diet groups based on Wald tests.

**Supplemental References**

1. Elliott P, Peakman TC. The UK Biobank sample handling and storage protocol for the collection, processing and archiving of human blood and urine. Int J Epidemiol. 2008;37:234–44.

2. UK Biobank Coordinating Centre. UK Biobank: Protocol for a large-scale prospective epidemiological resource [Internet]. 2007. Available from: http://www.ukbiobank.ac.uk/wp-content/uploads/2011/11/UK-Biobank-Protocol.pdf

3. UK Biobank, Fry D, Almond R, Moffat S, Gordon M, Singh P. UK Biobank Biomarker Project - Companion Document to Accompany Serum Biomarker Data Version 1.0 [Internet]. 2019. Available from: https://biobank.ndph.ox.ac.uk/showcase/showcase/docs/serum_biochemistry.pdf

4. UK Biobank, Tierney A, Fry D, Almond R, Gordon M, Moffat S. UK Biobank Biomarker Enhancement Project Companion Document to Accompany HbA1c Biomarker Data [Internet]. 2018. Available from: http://biobank.ctsu.ox.ac.uk/crystal/crystal/docs/serum_hb1ac.pdf

5. UK Biobank, Fry D, Almond R, Gordon M, Moffat S. UK Biobank Showcase. Details of assays and quality control information for the urinary biomarker data. v1.0. [Internet]. 2016. Available from: http://biobank.ndph.ox.ac.uk/showcase/showcase/docs/urine_assay.pdf

6. Dickerson RN, Alexander KH, Minard G, Croce MA, Brown RO. Accuracy of Methods to Estimate Ionized and “Corrected” Serum Calcium Concentrations in Critically Ill Multiple Trauma Patients Receiving Specialized Nutrition Support. J Parenter Enter Nutr. 2004;28:133–41.
